# Supplementary material for: On the usage of artificial intelligence in leprosy care: A systematic literature review
Source: PLoS Comput Biol. 2025 Jun 26;21(6):e1012550. doi: 10.1371/journal.pcbi.1012550 (PMC12225980; doi:10.1371/journal.pcbi.1012550)
Supplement: S1 Table — (PDF) [file pcbi.1012550.s001.pdf]

Id	title	data extractor	date of data extraction	author	journal	year	source	pages	volume	abstract	document_type	doi	url	affiliation	or_keywo	keywords	publisher	issn	nguag	note	lection_criter	Selection
1	Skin Diseases Detection Using LBP and WLD: An Ensembling Approach	Hilson Andrade	02/04/2025	Arnab BanerjeeSomenath SarkarMita Nasipu		2023	pringer Link					10.1007/s4	http://link.springer.com/article/10.1007/s429				SN Computer Science					Duplicated
2	Abstracts	Hilson Andrade	02/04/2025	unknown		2024	pringer Link					10.1007/s1	http://link.springer.com/article/10.1007/s120				Hepatology International					Duplicated
3	Prediction of rifampicin resistance beyond the RRDR using structure-based machine learning approaches	Hilson Andrade	02/04/2025	Portelli, Stephanie and My	Scientific Reports	2020	Scopus		10	Rifampicir	Article	10.1038/s4	https://www.scopus.com/inward/			Antitubercular Agents; Bacterial Proteins; I				Cited by: 34; All Open A		Duplicated
4	Deep Facial Diagnosis: Deep Transfer Learning from Face Recognition to Facial Diagnosis	Hilson Andrade	02/04/2025	Jin, Bo and Cruz, Leandro	IEEE Access	2020	Scopus	49 – 12	8	The relati	Article	10.1109/A	https://www.scopus.c		beta-thala	Computer aided instruction; Deep learning				Cited by: 175; All Open A		Duplicated
5	The COVID-19 Drug and Gene Set Library	Hilson Andrade	02/04/2025	Kuleshov, Maxim V. and S	Patterns	2020	Scopus		1	In a short	Article	10.1016/j.p	https://www.scopus.c		COVID-19	Diseases; Genes; HTTP; Machine learning				Cited by: 48; All Open A		Duplicated
6	Leprosy lesion recognition using convolutional neural networks	Hilson Andrade	02/04/2025	Baweja, Harjatin Singh an	Proceedings - Inte	2016	Scopus	41 – 14	1	Leprosy, a	Conference paper	10.1109/IC	https://www.scopus.c		Artificial ne	Computer vision; Convolution; Diagnosis; I				Cited by: 17		Duplicated
7	Hypersymbiotics™: An artistic reflection on the ethical and environmental implications of microbiome research and new technologies	Hilson Andrade	02/04/2025	Dumitriu, Anna	Endeavour	2022	Scopus		46	This essa	Article	10.1016/j.e	https://www.scopus.c		Antibiotic r	Art; Artificial Intelligence; Gene Editing; Hu				Cited by: 2		Duplicated
8	Fuzzy spectral clustering for automated delineation of chronic wound region using digital images	Hilson Andrade	02/04/2025	Dhiraj {Manohar Dhane} a	Computers in Biolo	2017	ence@Dir	551-560	89	Chronic wound is an abnormal c		https://doi.	https://www.sciencedirect.com/s			Chronic wound, Colo		0010-4825				Duplicated
9	Simulated multimodal deep facial diagnosis	Hilson Andrade	02/04/2025	Bo Jin and Nuno Gonçalv	Expert Systems wi	2024	ence@Dir	123881	252	Facial phenotypes are extensive		https://doi.	https://www.sciencedirect.com/s			Deep facial diagnosis		0957-4174				Duplicated
10	Ensemble optimization algorithm for the prediction of melanoma skin cancer	Hilson Andrade	02/04/2025	Sachin Gupta and Jayanth	Measurement: Ser	2023	ence@Dir	100887	29	One of the worst illnesses in the		https://doi.	https://www.sciencedirect.com/s			Machine learning, En		2665-9174				Duplicated
11	In vivo partial reprogramming by bacteria promotes adult liver organ growth without fibrosis and tumorigenesis	Hilson Andrade	02/04/2025	Samuel Hess and Timothy	Cell Reports Medi	2022	ence@Dir	100820	3	Summary		https://doi.	https://www.sciencedirect.com/s			organ growth, liver re		2666-3791				Duplicated
12	Mobile Phone-Based Microscopy, Sensing, and Diagnostics	Hilson Andrade	02/04/2025	Contreras-Naranjo, Jose (	IEEE Journal on S	2016	Scopus		22	Mass proc	Article	10.1109/JS	https://www.scopus.c		Diagnostic	Competition; Computer graphics; Consum				Cited by: 121; All Open A		Surveys
13	A structure-and chemical genomics-based approach for repositioning of drugs against VCP/p97 ATPase	Hilson Andrade	02/04/2025	Segura-Cabrera, Aldo and	Scientific Reports	2017	Scopus		7	Valosin-cc	Article	10.1038/sr	https://www.scopus.com/inward/			Allosteric Regulation; Binding Sites; Drug I				Cited by: 21; All Open A		Not related to AI
14	NGO–researcher partnerships in global health research: benefits, challenges, and approaches that promote success	Hilson Andrade	02/04/2025	Olivier, Catherine and Hur	Development in Pr	2016	Scopus	44 – 45	26	Partnersh	Article	10.1080/0	https://www.scopus.c		Aid – Deve	academic research; civil society; health ca				Cited by: 35; All Open A		Not related to AI
15	A Semantic Approach of Building Dynamic Learner Profile Model Using WordNet	Hilson Andrade	02/04/2025	Sheeba, T. and Krishnan,	Advances in Intelli	2020	Scopus	63 – 27	1082	The learn	Conference paper	10.1007/9	https://www.scopus.c		Dynamic le	Classification (of information); Intelligent c				Cited by: 2		Not related to leprosy
16	COVID-19 in LMICs: The Need to Place Stigma Front and Centre to Its Response	Hilson Andrade	02/04/2025	Roelen, Keetie and Ackley	European Journal	2020	Scopus	92 – 16	32	COVID-19	Article	10.1057/s4	https://www.scopus.c		Coronaviru	Coronavirus; COVID-19; disease control; I				Cited by: 42; All Open A		Not related to AI
17	Digital and experimental synergies to design high-heeled shoes	Hilson Andrade	02/04/2025	Milazzo, Mario and Spezz	International Journ	2020	Scopus	85 – 39	109	Digital tec	Article	10.1007/s0	https://www.scopus.c		Digital ma	Economic and social effects; Employment;				Cited by: 6		Not related to AI
18	Synovial tissue transcriptomes of long-standing rheumatoid arthritis are dominated by activated macrophages that reflect microbial stimulat	Hilson Andrade	02/04/2025	Smiljanovic, Biljana and G	Scientific Reports	2020	Scopus		10	Advances	Article	10.1038/s4	https://www.scopus.com/inward/			Adaptive Immunity; Arthritis, Rheumatoid;				Cited by: 25; All Open A		Not related to AI
19	On the ability to reconstruct ancestral genomes from Mycobacterium genus	Hilson Andrade	02/04/2025	Guyeux, Christophe and A	Lecture Notes in C	2017	Scopus	42 – 65	10208 LNCS	Technical	Conference paper	10.1007/9	https://www.scopus.c		Ancestral	Approximation algorithms; Bioinformatics;				Cited by: 3		Not related to leprosy
20	Positive deviance, big data, and development: A systematic literature review	Hilson Andrade	02/04/2025	Albanna, Basma and Hee	Electronic Journal	2019	Scopus		85	Positive d	Article	10.1002/is	https://www.scopus.c		big data; developing countries; machine learning; mo					Cited by: 29; All Open A		Not related to AI
21	A Spark-based workflow for probabilistic record linkage of healthcare data	Hilson Andrade	02/04/2025	Pita, Robespierre and Pin	CEUR Workshop P	2015	Scopus	17 – 26	1330	Several a	Conference paper		https://www.scopus.com/inward/			Application programming interfaces (API);				Cited by: 19		Not related to AI
22	Genomics and machine learning for taxonomy consensus: The mycobacterium tuberculosis complex paradigm	Hilson Andrade	02/04/2025	Azé, Jérôme and Sola, Ch	PLoS ONE	2015	Scopus		10	Infra-spec	Article	10.1371/jo	https://www.scopus.com/inward/			Algorithms; Bacterial Typing Techniques; C				Cited by: 23; All Open A		Not related to leprosy
23	Improving ancient DNA genome assembly	Hilson Andrade	02/04/2025	Seitz, Alexander and Nies	German Conferen	2016	Scopus			Most reco	Conference paper	10.7287/p	https://www.scopus.c		ancient DN	DNA; Genome; Ancient DNA; De novo ass				Cited by: 0; All Open Acc		Not related to AI
24	Improving efficiency of remote data audit for cloud storage	Hilson Andrade	02/04/2025	Fan, Kuan and Liu, Mingx	KSII Transactions	2019	Scopus	98 – 22	13	The cloud	Article	10.3837/tii	https://www.scopus.c		Auditing re	Efficiency; Polypropylenes; Cloud service				Cited by: 1; All Open Acc		Not related to leprosy
25	Windowing improvements towards more comprehensible models	Hilson Andrade	02/04/2025	Perez, Pedro Santoro and	Knowledge-Based	2016	Scopus	9 – 22	92	The induc	Article	10.1016/j.k	https://www.scopus.c		Decision tr	Clustering algorithms; Data mining; Decisi				Cited by: 2		Not related to leprosy
26	Blame-game politics: Re-evaluating incongruent leprosy and covid-19 policies in the old testament and nigerian societies	Hilson Andrade	02/04/2025	Agbo, Paulinus O. and Ok	Verbum et Ecclesia	2021	Scopus		42	History is	Article	10.4102/ve	https://www.scopus.c		Blame-game; COVID-19 policy; Leprosy; Nigeria; Old					Cited by: 0; All Open Acc		Not related to AI
27	Semantic Changepoint Detection for Finding Potentially Novel Research Publications	Hilson Andrade	02/04/2025	Dinakar, Bhavish and Bog	Pacific Symposium	2021	Scopus	07 – 118		How has t	Conference paper		https://www.scopus.c		Changepo	Computational Biology; COVID-19; Human				Cited by: 1		Not related to AI
28	The planning and design of road network structure in urban railway transit hub areas	Hilson Andrade	02/04/2025	Cai, Zhaoyang and Yan, J	International Journ	2018	Scopus	98 – 20	13	Urban rail	Article	10.1093/ijl	https://www.scopus.c		Design an	Highway planning; Railroads; Roads and s				Cited by: 3; All Open Acc		Not related to leprosy
29	Design model of public facilities topology based on manifold learning and big data optimization	Hilson Andrade	02/04/2025	Wang, Long	Boletin Tecnico/Te	2017	Scopus	04 – 71	55	Topology	Article		https://www.scopus.c		Data Optir	Behavioral research; Big data; Design; Pu				Cited by: 0		Not related to leprosy
30	Assessing the performance of genome-wide association studies for predicting disease risk	Hilson Andrade	02/04/2025	Patron, Jonas and Serra-(	PLoS ONE	2019	Scopus		14	To date m	Article	10.1371/jo	https://www.scopus.com/inward/			Databases, Genetic; Genetic Predispositic				Cited by: 38; All Open A		Not related to AI
31	Computational de novo discovery of distinguishing genes for biological processes and cell types in complex tissues	Hilson Andrade	02/04/2025	Newberg, Lee A. and Che	PLoS ONE	2018	Scopus		13	Bulk tissu	Article	10.1371/jo	https://www.scopus.com/inward/			Algorithms; Animals; B-Lymphocytes; Brai				Cited by: 8; All Open Acc		Not related to leprosy
32	Prediction of Plantar Shear Stress Distribution by Conditional GAN with Attention Mechanism	Hilson Andrade	02/04/2025	Guo, Jinghui and Ersen, A	Lecture Notes in C	2020	Scopus	70 – 78	12262 LNCS	Diabetic fo	Conference paper	10.1007/9	https://www.scopus.c		Conditiona	Diseases; Forecasting; Mean square error				Cited by: 1		Not related to AI
33	Characterization of the COVID-19 pandemic and the impact of uncertainties, mitigation strategies, and underreporting of cases in South Ko	Hilson Andrade	02/04/2025	Reis, Ruy Freitas and de I	Chaos, Solitons ar	2020	Scopus		136	By April 7t	Article	10.1016/j.c	https://www.scopus.c		COVID-19	Disease control; Estimation; Sensitivity an				Cited by: 92; All Open A		Not related to leprosy
34	AHP Model for Diagnosis of Tropical Confusable Diseases	Hilson Andrade	02/04/2025	Uzoka, Faith-Michael E. a	Proceedings - 201	2018	Scopus	58 – 1763		Access to	Conference paper	10.1109/C	https://www.scopus.c		Analytic Hi	Analytic hierarchy process; Artificial intellig				Cited by: 6		Not related to leprosy
35	"It could have been us in a different moment. It still is us in many ways": Community identification and the violence of archival representatio	Hilson Andrade	02/04/2025	Brilmyer, Gracen M.	Lecture Notes in C	2020	Scopus	80 – 48	12051 LNCS	Using data	Conference paper	10.1007/9	https://www.scopus.c		Archives; I	Computer science; Computers; Communit				Cited by: 5		Not related to AI
36	Prognosis model for gestational diabetes using machine learning techniques	Hilson Andrade	02/04/2025	Amarnath, Sumathi and S	Sensors and Mate	2021	Scopus	11 – 30	33	Gestation	Article	10.18494/	https://www.scopus.c		Classificat	Classification (of information); Data mining				Cited by: 5; All Open Acc		Not related to AI
37	Pilot deployment of a cloud-based universal medical image repository in a large public health system: A protocol study	Hilson Andrade	02/04/2025	Pacheco, Viviane Margari	PLoS ONE	2024	Scopus		19	This pape	Article	10.1371/jo	https://www.scopus.com/inward/			Artificial Intelligence; Cloud Computing; Co				Cited by: 0; All Open Acc		Not related to leprosy
38	Dysregulated Wnt and NFAT signaling in a Parkinson's disease LRRK2 G2019S knock-in model	Hilson Andrade	02/04/2025	Wetzel, Andrea and Lei, S	Scientific Reports	2024	Scopus		14	Parkinson	Article	10.1038/s4	https://www.scopus.c		Immune sy	Animals; Brain; Disease Models, Animal; F				Cited by: 1; All Open Acc		Not related to AI
39	A Combination of Data Mining Methods for Disease Classification Using Patient-Perceived Symptoms from Medical Records	Hilson Andrade	02/04/2025	Mumtazah, Nadya and Wa	Proceedings - Inte	2024	Scopus	26 – 431		One of the	Conference paper	10.1109/IC	https://www.scopus.c		data minin	Diseases; Hospital data processing; Neare				Cited by: 0		Not related to leprosy
40	Sentiment Analysis by Lexical Analysis Combined with Machine Learning	Hilson Andrade	02/04/2025	Ho, Van Lam and Minh, V	International Journ	2023	Scopus	07 – 32	15	This pape	Article	10.15849/I	https://www.scopus.c		classifying sentences; lexical analysis; machine learn					Cited by: 1		Not related to leprosy
41	A survey on classification of Geospatial Clustering	Hilson Andrade	02/04/2025	Kansakar, Aman Ratna ar	2023 International	2023	Scopus	41 – 446		Machine l	Conference paper	10.1109/C	https://www.scopus.com/inward/			Image segmentation; Machine learning; M				Cited by: 1		Not related to leprosy
42	The Effect of Heel Base Design on Plantar Pressure and Wear Comfort of Healthy Females	Hilson Andrade	02/04/2025	Luk, Sin-Hang and Yick, K	International Confe	2023	Scopus	87 – 93		As one of	Conference paper		https://www.scopus.c		comfort; heel base design; high heels; plantar pressu					Cited by: 0		Not related to leprosy
43	Geospatial Clustering: Methods, Applications and Future Directions	Hilson Andrade	02/04/2025	Sahay, Akshat and Pandit	2022 International	2022	Scopus			Geospatia	Conference paper	10.1109/IC	https://www.scopus.com/inward/			Cluster analysis; Land use; Clustering app				Cited by: 5		Not related to leprosy
44	Optimization of residents' dietary structure with consideration of greenhouse gas mitigation and nutritional requirements	Hilson Andrade	02/04/2025	Yue, Wencong and Tan, Z	Sustainable Produ	2022	Scopus	24 – 43	32	Food-rela	Article	10.1016/j.s	https://www.scopus.c		Food syste	Economic and social effects; Economics; E				Cited by: 8		Not related to AI
45	Optical Detection of Winged Insects Utilizing Wireless Sensor Network with Ant Colony Optimizer	Hilson Andrade	02/04/2025	Evangelista, Ivan Roy S. a	2023 8th Internatic	2023	Scopus	32 – 638		Flies and	Conference paper	10.1109/IC	https://www.scopus.c		ant colony	Ant colony optimization; Artificial intelligen				Cited by: 2		Not related to leprosy
46	Within-host mathematical modeling on crucial inflammatory mediators and drug interventions in COVID-19 identifies combination therapy t	Hilson Andrade	02/04/2025	Chhetri, Bishal and Bhaga	Alexandria Engine	2021	Scopus	91 – 25	60	The unpre	Article	10.1016/j.a	https://www.scopus.c		Covid-19;	Cell proliferation; Decision making; Epiden				Cited by: 26; All Open A		Not related to leprosy
47	Harvesting Hope: Empowering Self-Help Groups through AI-Driven Sentiment Analysis for Sustainable Futures	Hilson Andrade	02/04/2025	Ghosh, Sameek and Nair,	15th International	2024	Scopus	54 – 22	2	Self-help	Conference paper		https://www.scopus.com/inward/			Artificial intelligence techniques; Communi				Cited by: 0		Not related to leprosy
48	Social determinants in the access to health care for Chagas disease: A qualitative research on family life in the "Valle Alto" of Cochabamba	Hilson Andrade	02/04/2025	Jimeno, I. and Mendoza, I	PLoS ONE	2021	Scopus		16	Introducti	Article	10.1371/jo	https://www.scopus.com/inward/			Bolivia; Chagas Disease; Decision Making				Cited by: 7; All Open Acc		Not related to leprosy
49	Assessment of Ambient Air Pollution Levels: A Five-Year Analysis of PM2.5, NO, NO2, NOX, CO, and SO2	Hilson Andrade	02/04/2025	Srivastava, Rohan and Kh	Rocznik Ochrona S	2024	Scopus	66 – 37	26	The study	Article	10.54740/	https://www.scopus.c		Air Pollutants; Ambient air quality standards; CO; Har					Cited by: 0; All Open Acc		Not related to AI
50	Next-Generation Skin Disease Diagnosis: Deep Learning Solution	Hilson Andrade	02/04/2025	Bhatt, Chandradeep and S	Proceedings - 202	2024	Scopus	66 – 372		Skin cond	Conference paper	10.1109/TI	https://www.scopus.c		Alexnet; de	Deep learning; Dermatology; Alexnet; Bac				Cited by: 0		Not related to leprosy
51	In-shoe plantar shear stress sensor design, calibration and evaluation for the diabetic foot	Hilson Andrade	02/04/2025	Haron, Athia H. and Li, Lu	PLoS ONE	2024	Scopus		19	Plantar sh	Article	10.1371/jo	https://www.scopus.com/inward/			Adult; Biomechanical Phenomena; Calibra				Cited by: 1; All Open Acc		Not related to AI
52	Trends of unsuccessful treatment outcomes and associated factors among tuberculosis patients in public hospitals of Bale Zone, Southeas	Hilson Andrade	02/04/2025	Zenbaba, Demisu and Bo	Heliyon	2021	Scopus		7	Introducti	Article	10.1016/j.h	https://www.scopus.c		Bale zone; Southeast Ethiopia; Treatment outcome; T					Cited by: 5; All Open Acc		Not related to leprosy
53	Bioarcheology of bone remains from medieval burials from Armenia*	Hilson Andrade	02/04/2025	Khudaverdyan, Anahit Yu	Bulletin of the Inter	2022	Scopus	38 – 26	16	The aim o	Article		https://www.scopus.c		Armenia; artificial cranial modification; Early Middle A					Cited by: 0		Not related to leprosy
54	An Improved Deep Learning Framework Approach for Detecting the Psoriasis Disease	Hilson Andrade	02/04/2025	Singh, Anuj and Kumar, K	Proceedings of the	2023	Scopus			Normal sk	Conference paper	10.1109/IC	https://www.scopus.c		deep learn	Deep learning; Dermatology; Learning alg				Cited by: 2		Not related to leprosy
55	Bioarchaeological analyses reveal long-lasting continuity at the periphery of the Late Antique Roman Empire	Hilson Andrade	02/04/2025	Depaermentier, Margaux I	iScience	2023	Scopus		26	The Base	Article	10.1016/j.i	https://www.scopus.c		Anthropology; Archeology; Evolutionary biology; Hum					Cited by: 0; All Open Acc		Not related to leprosy
56	Tryp: a dataset of microscopy images of unstained thick blood smears for trypanosome detection	Hilson Andrade	02/04/2025	Anzaku, Esla Timothy and	Scientific Data	2023	Scopus		10	Trypanos	Data paper	10.1038/s4	https://www.scopus.com/inward/			Animals; Artificial Intelligence; Humans; Mi				Cited by: 0; All Open Acc		Not related to leprosy
57	CROSS-CULTURAL ADAPTATION OF THE HOSPITAL ETHICAL CLIMATE SURVEY TO BRAZIL	Hilson Andrade	02/04/2025	Lanes, Taís Carpes and D	Journal of Nursing	2023	Scopus	48 – 16	31	Backgrou	Article	10.1891/JI	https://www.scopus.c		ethics; hos	Brazil; Cross-Cultural Comparison; Hospit				Cited by: 5		Not related to leprosy
58	An Information Extraction Method for Industrial and Commercial Rooftop Photovoltaics Based on GaoFen-7 Remote Sensing Images	Hilson Andrade	02/04/2025	Tao, Haoxiang and He, Gu	Remote Sensing	2023	Scopus		15	With the i	Article	10.3390/rs	https://www.scopus.c		distributed	Antennas; Image segmentation; Industrial				Cited by: 0; All Open Acc		Not related to leprosy
59	Inferring household size distribution and its association with the built environment using massive mobile phone data	Hilson Andrade	02/04/2025	Lai, Jianhui and Luo, Tian	Cities	2023	Scopus		136	Househol	Article	10.1016/j.c	https://www.scopus.c		Built enviro	Beijing [Beijing (ADS)]; Beijing [China]; Ch				Cited by: 2		Not related to leprosy
60	Identification of host genomic biomarkers from multiple transcriptomics datasets for diagnosis and therapies of SARS-CoV-2 infections	Hilson Andrade	02/04/2025	Sarker, Bandhan and Rah	PLoS ONE	2023	Scopus		18	The pand	Article	10.1371/jo	https://www.scopus.com/inward/			Aurora Kinase A; Biomarkers; COVID-19;				Cited by: 6; All Open Acc		Not related to leprosy
61	A Novel Flexible Multidimensional Force Platform for Assessing Regional Plantar Pressure and Shear Stress Under the Foot During Gait	Hilson Andrade	02/04/2025	Luo, Hu and Zhang, Xingy	IEEE Sensors Jou	2024	Scopus	23 – 11	24	The multi	Article	10.1109/JS	https://www.scopus.c		Deep-lear	Calibration; Clinical research; Deep learnir				Cited by: 2		Not related to leprosy
62	Skin Disease Identification using online and Offline Data Prediction using CNN Classification	Hilson Andrade	02/04/2025	Sonawane, Minakshi M. a	14th International	2023	Scopus	63 – 24	2023-June	In this stu	Conference paper		https://www.scopus.c		CNN; Con	Classification (of information); Convolution				Cited by: 1		Not related to leprosy
63	Metagenomic analysis of Mesolithic chewed pitch reveals poor oral health among stone age individuals	Hilson Andrade	02/04/2025	Kırdök, Emrah and Kashu	Scientific Reports	2023	Scopus		13	Prehistori	Article	10.1038/s4	https://www.scopus.com/inward/			Animals; Deer; Dysbiosis; Humans; Metag				Cited by: 3; All Open Acc		Not related to leprosy
64	Pulmonary adiaspiromycosis in armadillos killed by motor vehicle collisions in Brazil	Hilson Andrade	02/04/2025	Navas-Suárez, Pedro Enr	Scientific Reports	2021	Scopus		11	Knowledg	Article	10.1038/s4	https://www.scopus.com/inward/			Animals; Armadillos; Brazil; Lung Diseases				Cited by: 4; All Open Acc		Not related to leprosy
65	A novel text analytic methodology for classification of product and service reviews	Hilson Andrade	02/04/2025	Yucel, Ahmet and Dag, Ali	Journal of Busines	2022	Scopus	87 – 29	151	Classifyin	Article	10.1016/j.j	https://www.scopus.c		Business analytics; Classification prediction; Data mi					Cited by: 9		Not related to leprosy
66	Bootstrapping of fine-tuned segmentation and classification network for epidermis disorder categorization	Hilson Andrade	02/04/2025	Kalaivani, A. and Karpaga	Multimedia Tools a	2024	Scopus	07 – 18	83	As all peo	Article	10.1007/s1	https://www.scopus.c		Bootstrapp	Classification (of information); Deep neura				Cited by: 0		Not related to leprosy
67	A qualitative exploration of tuberculosis patients who were lost to follow-up in Malaysia	Hilson Andrade	02/04/2025	Tok, Peter Seah Keng and	PLoS ONE	2023	Scopus		18	Backgrou	Article	10.1371/jo	https://www.scopus.com/inward/			Communication; Drug-Related Side Effects				Cited by: 1; All Open Acc		Not related to leprosy
68	Comprehensive Data Collection and Feedback Algorithm for Smart Music Online Guidance based on Immersive Virtual Media Environmen	Hilson Andrade	02/04/2025	Gong, Qinghua	5th International C	2022	Scopus	35 – 1339		Comprehe	Conference paper	10.1109/IC	https://www.scopus.c		data collec	Data acquisition; Data handling; E-learning				Cited by: 0		Not related to leprosy
69	Local Metric Learning for Off-Policy Evaluation in Contextual Bandits with Continuous Actions	Hilson Andrade	02/04/2025	Lee, Haanvid and Lee, Jo	Advances in Neura	2022	Scopus		35	We consi	Conference paper		https://www.scopus.com/inward/			Learning algorithms; Mean square error; V				Cited by: 3		Not related to leprosy
70	Recognizing Common Skin Diseases in the Philippines Using YOLOv5 Deep Learning	Hilson Andrade	02/04/2025	Tomas, Mary Christine A.	Proceedings of SP	2024	Scopus		13274	This study	Conference paper	10.1117/12	https://www.scopus.c		Dermatolo	Dermatology; Medical image processing; D				Cited by: 0		Not related to leprosy
71	Predictive model for converting optic neuritis to multiple sclerosis; decision tree in focus	Hilson Andrade	02/04/2025	Rasouli, Saeid and Dakka	PLoS ONE	2024	Scopus		19	Backgrou	Article	10.1371/jo	https://www.scopus.com/inward/			Adolescent; Adult; Decision Trees; Female				Cited by: 0; All Open Acc		Not related to leprosy
72	Detection of Skin Allergy Causing Chemicals in Textile Fibers using Principal Component Analysis and Support Vector Machine	Hilson Andrade	02/04/2025	Balbin, Jessie R. and Bau	ACM International	2021	Scopus	15 – 19		This pape	Conference paper	10.1145/3	https://www.scopus.c		Pattern Re	Chemical detection; Learning algorithms; F				Cited by: 0		Not related to leprosy
73	Using machine learning methods to detect physical conditions with postural balance	Hilson Andrade	02/04/2025	Chou, Chinmei and Chou,	Journal of Ambient	2023	Scopus	99 – 14	14	Previous	Article	10.1007/s1	https://www.scopus.c		Center of	Health; Machinery; Balance measurement				Cited by: 2		Not related to leprosy
74	Insights into Dermatological Disorders: Understanding Skin Diseases through Medical Image Analysis	Hilson Andrade	02/04/2025	Dash, Yajnaseni and Ram	International Journ	2024	Scopus	13 – 62	16	Skin disea	Article		https://www.scopus.c		Convolutional Neural Networks (CNNs); skin disease					Cited by: 0		Not related to leprosy
75	Experimental investigation on the thermal appraisal and sustainability indicator of heat pipe-based indirect solar dryer during Giloy (Tinospo	Hilson Andrade	02/04/2025	Dhiraj {Kumar Yadav} and	Thermal Science a	2024	ence@Dir	102335	47	Tinospora cordifolia (Giloy) is ar		https://doi.	https://www.sciencedirect.com/s			Giloy stem, Exergy, S		2451-9049				Not related to leprosy
76	Evolution of bioinformatics and its impact on modern bio-science in the twenty-first century: Special attention to pharmacology, plant scienc	Hilson Andrade	02/04/2025	Debasis Mitra and Debanj	Computational Tox	2022	ence@Dir	100248	24	Bioinformatics is inherently an ir		https://doi.	https://www.sciencedirect.com/s			Bioengineering, Bioin		2468-1113				Not related to leprosy
77	Infections and systemic inflammation are associated with lower plasma concentration of insulin-like growth factor I among Malawian childre	Hilson Andrade	02/04/2025	Kenneth Maleta and Yue-I	The American Jou	2021	ence@Dir	380-390	113	ABSTRACT		https://doi.	https://www.sciencedirect.com/s			childhood growth falte		0002-9165				Not related to AI
78	InDetector – Automatic detection of infected driveline regions	Hilson Andrade	02/04/2025	Shravan Aras and Thienne	Smart Health	2018	ence@Dir	170-178	9-10	Although there have been signif		https://doi.	https://www.sciencedirect.com/science/article/pii/S235					2352-6483		CHASE 2018 Special Is		Not related to leprosy
79	Differentiated impacts of short-term exposure to fine particulate constituents on infectious diseases in 507 cities of Chinese children and ad	Hilson Andrade	02/04/2025	Li Chen and Wen Yuan ar	Science of The Tot	2024	ence@Dir	172299	928	This study assesses the associa		https://doi.	https://www.sciencedirect.com/s			PM, PM constituents,		0048-9697				Not related to leprosy
80	Viral surveillance: Governing social isolation in São Paulo, Brazil, during the COVID-19 Pandemic	Hilson Andrade	02/04/2025	Alcides Eduardo dos Reis	Social Sciences &	2021	ence@Dir	100128	3	In the context of COVID-19 pand		https://doi.	https://www.sciencedirect.com/s			Surveillance, COVID-		2590-2911				Not related to leprosy
81	Reinforcement learning-based decision support system for COVID-19	Hilson Andrade	02/04/2025	Regina Padmanabhan an	Biomedical Signal	2021	ence@Dir	102676	68	Globally, informed decision on th		https://doi.	https://www.sciencedirect.com/s			COVID-19, Reinforce		1746-8094				Not related to leprosy
82	Innovation applications of MXenes in biomedicine	Hilson Andrade	02/04/2025	Ali Mohammad Amani anc	Materials Today Co	2024	ence@Dir	109929	40	MXenes, a class of two-dimensi		https://doi.	https://www.sciencedirect.com/s			MXenes, Surface mo		2352-4928				Not related to leprosy
83	The drug repurposing landscape from 2012 to 2017: evolution, challenges, and possible solutions	Hilson Andrade	02/04/2025	Prasanthi Polamreddy anc	Drug Discovery To	2019	ence@Dir	789-795	24	As the name suggests, drug rep		https://doi.	https://www.sciencedirect.com/science/article/pii/S135					1359-6446				Not related to leprosy
84	A computational study to disclose potential drugs and vaccine ensemble for COVID-19 conundrum	Hilson Andrade	02/04/2025	Sajjad Ahmad and Yasir W	Journal of Molecul	2021	ence@Dir	114734	324	The nucleocapsid (N) protein of		https://doi.	https://www.sciencedirect.com/s			SARS-COV-2, Nuclec		0167-7322				Not related to leprosy
85	Privacy-driven access control in social networks by means of automatic semantic annotation	Hilson Andrade	02/04/2025	Malik Imran-Daud and Da	Computer Commu	2016	ence@Dir	12-25	76	In online social networks (OSN)		https://doi.	https://www.sciencedirect.com/s			Social networks, Acce		0140-3664				Not related to leprosy
86	Molecularly imprinted polymer-based nanodiagnostics for clinically pertinent bacteria and virus detection for future pandemics	Hilson Andrade	02/04/2025	Amit K. Yadav and Damin	Biosensors and Bi	2022	ence@Dir	100257	12	There seems to be a growing cu		https://doi.	https://www.sciencedirect.com/s			Biosensors, Molecula		2590-1370				Not related to leprosy
87	Pathways to a healthy net-zero future: report of the Lancet Pathfinder Commission	Hilson Andrade	02/04/2025	Sarah Whitmee and Rose	The Lancet	2024	ence@Dir	67-110	403			https://doi.	https://www.sciencedirect.com/science/article/pii/S014					0140-6736				Not related to leprosy
88	Global, regional, and national age-specific progress towards the 2020 milestones of the WHO End TB Strategy: a systematic analysis for th	Hilson Andrade	02/04/2025	Jorge R Ledesma and Jia	The Lancet Infectio	2024	ence@Dir	698-725	24	Summary		https://doi.	https://www.sciencedirect.com/science/article/pii/S147					1473-3099				Not related to leprosy
89	Structural Mechanisms of Mitochondrial Quality Control Mediated by PINK1 and Parkin	Hilson Andrade	02/04/2025	Jean-François Trempe an	Journal of Molecul	2023	ence@Dir	168090	435	Parkinson's disease (PD) is the		https://doi.	https://www.sciencedirect.com/s			Parkinson's disease p		0022-2836		Molecular Mechanisms o		Not related to leprosy
90	A Showcase Presentation Honoring the Editorial Board of Clinics in Dermatology	Hilson Andrade	02/04/2025	Leonard J. Hoenig	Clinics in Dermato	2024	ence@Direct			For over forty years, the Editoria		https://doi.	https://www.sciencedirect.com/s			Editorial Board, Clinic		0738-081X				Not related to leprosy
91	Advances in drug metabolism and pharmacogenetics research in Australia	Hilson Andrade	02/04/2025	Peter I. Mackenzie and Ar	Pharmacological R	2017	ence@Dir	7-19	116	Metabolism facilitates the elimin		https://doi.	https://www.sciencedirect.com/s			Drug metabolism, Ph		1043-6618		Country in focus: Pharm		Not related to AI
92	Novel Assays/Applications for Patients Suspected of Mycobacterial Diseases	Hilson Andrade	02/04/2025	Niaz Banaei and Kimberle	Clinics in Laborato	2020	ence@Dir	535-552	40			https://doi.	https://www.sciencedirect.com/s			, Nontuberculous my		0272-2712		Current Issues in Clinica		Not related to leprosy
93	Targets of ubiquitin like system in mycobacteria and related actinobacterial species	Hilson Andrade	02/04/2025	Yusuf Akhter and Shweta	Microbiological Re	2017	ence@Dir	9-29	204	Protein turnover and recycling is		https://doi.	https://www.sciencedirect.com/s			Actinobacteria, Post-t		0944-5013				Not related to leprosy
94	Croton's therapeutic promise: A review of its phytochemistry and critical computational ADME/Tox analysis	Hilson Andrade	02/04/2025	Samphelix O. Obende anc	South African Jour	2024	ence@Dir	648-672	171	The genus Croton (Euphorbiace		https://doi.	https://www.sciencedirect.com/s			Croton, Diterpenoids,		0254-6299				Not related to AI
95	The inevitability of change	Hilson Andrade	02/04/2025	Rokea A. el-Azhary	Clinics in Dermato	2019	ence@Dir	4-11	37	Change is an absolute so long a		https://doi.	https://www.sciencedirect.com/science/article/pii/S073					0738-081X		De Profundis		Not related to leprosy
96	Smartphone Apps for Containing the COVID-19 Pandemic in Germany: Qualitative Interview Study With Experts Based on Grounded Theo	Hilson Andrade	02/04/2025	Dennis Krämer and Elisab	Journal of Medical	2023	ence@Direct		25	Background		https://doi.	https://www.sciencedirect.com/s			Corona-Warn-App, C		1438-8871				Not related to AI
97	Plantar pressure alterations associated with increased BMI in young adults	Hilson Andrade	02/04/2025	Kinda Khalaf and Dhanya	Gait & Posture	2022	ence@Dir	255-260	98	Background		https://doi.	https://www.sciencedirect.com/s			Increased BMI, Gait,		0966-6362				Not related to AI
98	Pharmacological profiles of neglected tropical disease drugs	Hilson Andrade	02/04/2025	Alessandro Greco and Re	Artificial Intelligenc	2024	ence@Dir	100116	6	According to the World health O		https://doi.	https://www.sciencedirect.com/s			Drug discovery, Drug		2667-3185				Not related to AI
99	Drug databases and their contributions to drug repurposing	Hilson Andrade	02/04/2025	Yosef Masoudi-Sobhanza	Genomics	2020	ence@Dir	087-109	112	Drug repurposing is an interestir		https://doi.	https://www.sciencedirect.com/s			Drug, Database, Drug		0888-7543				Not related to AI
100	A comprehensive analysis of contaminated groundwater: Special emphasis on nature-ecosystem and socio-economic impacts	Hilson Andrade	02/04/2025	Gaurav Chandnani and P	Groundwater for S	2022	ence@Dir	100813	19	Besides air, water is the most es		https://doi.	https://www.sciencedirect.com/s			Impacts, Groundwate		2352-801X				Not related to leprosy

Id	title	data extractor	date of data extraction	author	journal	year	source	pages	volume	abstract	document_type	doi	url	affiliation	or_keywo	keywords	publisher	issn	nguag	note	lection_criter	Selection
101	A proteomic road to acquire an accurate serological diagnosis for human tegumentary leishmaniasis	Hilson Andrade	02/04/2025	B.S.S. Lima and S.F. Pires	Journal of Proteon	2017	ence@Dir	174-181	151	Diagnostic tools are important fc		https://doi.	https://www.sciencedirect.com/s			Tegumentary leishma		1874-3919		Brazilian Proteomics		Not related to AI
102	Turning liabilities into opportunities: Off-target based drug repurposing in cancer	Hilson Andrade	02/04/2025	Vinayak Palve and Yi Liao	Seminars in Cance	2021	ence@Dir	209-229	68	Targeted drugs and precision m		https://doi.	https://www.sciencedirect.com/s			Targeted cancer thera		1044-579X		Drug Repurposing for Ca		Not related to AI
103	Privileged small molecules against neglected tropical diseases: A perspective from structure activity relationships	Hilson Andrade	02/04/2025	J. {Abbasi Shiran} and B.	European Journal	2024	ence@Dir	116396	271	Neglected tropical diseases (NT		https://doi.	https://www.sciencedirect.com/s			NTD, Parasite inhibiti		0223-5234				Surveys
104	The importance of iteration in creative conceptual combination	Hilson Andrade	02/04/2025	Joel Chan and Christian D	Cognition	2015	ence@Dir	104-115	145	Theories of creative conceptual		https://doi.	https://www.sciencedirect.com/s			Creativity, Problem sc		0010-0277				Not related to leprosy
105	Identification of Keratinocyte Cytoprotectants against Toxicity by the Multikinase Inhibitor Sorafenib Using Drug Repositioning	Hilson Andrade	02/04/2025	Yayoi Kamata and Rui Ka	JID Innovations	2024	ence@Dir	100271	4	Hand–foot skin reaction is the m		https://doi.	https://www.sciencedirect.com/s			Apoptosis, Drug deve		2667-0267				Not related to AI
106	The role of the methoxy group in approved drugs	Hilson Andrade	02/04/2025	Debora Chiodi and Yoshih	European Journal	2024	ence@Dir	116364	273	The methoxy substituent is prev		https://doi.	https://www.sciencedirect.com/science/article/pii/S022					0223-5234				Not related to AI
107	The History of Dermatology and Dermatologists at the US Food and Drug Administration	Hilson Andrade	02/04/2025	Vanessa L. Burrows and M	Dermatologic Clini	2022	ence@Dir	237-248	40			https://doi.	https://www.sciencedirect.com/s			Dermatology, History,		0733-8635		Food and Drug Administ		Not related to AI
108	Proteomics research in India: An update	Hilson Andrade	02/04/2025	Panga Jaipal Reddy and A	Journal of Proteon	2015	ence@Dir	7-17	127	After a successful completion of		https://doi.	https://www.sciencedirect.com/s			India, Proteomics, Dr		1874-3919		Special Issue "Proteomi		Not related to leprosy
109	Artificial neural network modeling of antimycobacterial chemical space to introduce efficient descriptors employed for drug design	Hilson Andrade	02/04/2025	Soroush Sardari and Hous	Chemometrics anc	2014	ence@Dir	151-158	130	Tuberculosis has become a seri		https://doi.	https://www.sciencedirect.com/s			Computational biolog		0169-7439				Not related to leprosy
110	Application of antimicrobial, potential hazard and mitigation plans	Hilson Andrade	02/04/2025	Shing Ching Khoo and Me	Environmental Res	2022	ence@Dir	114218	215	The tremendous rise in the cons		https://doi.	https://www.sciencedirect.com/s			Antimicrobial resistan		0013-9351				Not related to AI
111	New Coumarin-Pyrazole hybrids: Synthesis, Docking studies and Biological evaluation as potential cholinesterase inhibitors	Hilson Andrade	02/04/2025	Amina Benazzouz-Touam	Journal of Molecul	2022	ence@Dir	131591	1249	A new set of hybrid derivatives t		https://doi.	https://www.sciencedirect.com/s			Alzheimer's disease,		0022-2860				Not related to AI
112	Chapter Five - The Role of Spatial Statistics in the Control and Elimination of Neglected Tropical Diseases in Sub-Saharan Africa: A Focus	Hilson Andrade	02/04/2025	M.C. Stanton		2017	ence@Dir	187-241	97	Disease control and elimination		https://doi.	https://www.sciencedirect.com/s			Disease m	Academic	0065-308X				Not related to AI
113	Generating gene ontology-disease inferences to explore mechanisms of human disease at the comparative toxicogenomics database	Hilson Andrade	02/04/2025	Davis, Allan Peter and Wi	PLoS ONE	2016	Scopus		11	Strategies	Article	10.1371/jo	https://www.scopus.com/inward/			Computational Biology; Databases, Genet				Cited by: 27; All Open Ac		Not related to AI
114	Nociception, Pain, Negative Moods, and Behavior Selection	Hilson Andrade	02/04/2025	Marwan N. Baliki and A. V	Neuron	2015	ence@Dir	474-491	87	Recent neuroimaging studies su		https://doi.	https://www.sciencedirect.com/science/article/pii/S089					0896-6273				Not related to AI
115	The Role of Imaging for Disorders of Peripheral Nerve	Hilson Andrade	02/04/2025	Natalia L. Gonzalez and L	Clinics in Geriatric	2021	ence@Dir	223-239	37			https://doi.	https://www.sciencedirect.com/s			Nerve imaging, Nerve		0749-0690		Peripheral Nerve Diseas		Not related to AI
116	The epidemiology, pathogenesis, transmission, diagnosis, and management of multidrug-resistant, extensively drug-resistant, and incurabl	Hilson Andrade	02/04/2025	Keertan Dheda and Tawa	The Lancet Respir	2017	ence@Dir	291-360	5	Summary		https://doi.	https://www.sciencedirect.com/science/article/pii/S221					2213-2600				Not related to AI
117	Psoriasis skin biopsy image segmentation using Deep Convolutional Neural Network	Hilson Andrade	02/04/2025	Anabik Pal and Utpal Gar	Computer Methods	2018	ence@Dir	59-69	159	Background and objective		https://doi.	https://www.sciencedirect.com/s			Psoriasis Biopsy imag		0169-2607				Not related to leprosy
118	Crbn-based molecular Glues: Breakthroughs and perspectives	Hilson Andrade	02/04/2025	Juzeng An and Xiaojun Zh	Bioorganic & Medi	2024	ence@Dir	117683	104	CRBN is a substrate receptor fo		https://doi.	https://www.sciencedirect.com/s			CRBN, Molecular Glu		0968-0896				Not related to AI
119	Immunology, immunopathogenesis and immunotherapeutics of COVID-19; an overview	Hilson Andrade	02/04/2025	Leila {Mohamed Khosrosh	International Immu	2021	ence@Dir	107364	93	Coronavirus disease 2019 (CO\		https://doi.	https://www.sciencedirect.com/s			SARS-CoV-2, COVID		1567-5769				Not related to AI
120	Recent advances in developing small-molecule inhibitors against SARS-CoV-2	Hilson Andrade	02/04/2025	Rong Xiang and Zhengse	Acta Pharmaceutic	2022	ence@Dir	591-162	12	The COVID-19 pandemic cause		https://doi.	https://www.sciencedirect.com/s			SARS-CoV-2, COVID		2211-3835				Not related to AI
121	Pharmacovigilance of herbal medicines: Concerns and future prospects	Hilson Andrade	02/04/2025	Abinash Choudhury and F	Journal of Ethnoph	2023	ence@Dir	116383	309	Ethnopharmacological relevanc		https://doi.	https://www.sciencedirect.com/s			Adverse drug reactior		0378-8741				Not related to AI
122	Identification of decedents by restoring mummified fingerprints: Forensic dermatology in the investigation of mummy dermatoglyphics	Hilson Andrade	02/04/2025	Philip R. Cohen and Bora	Clinics in Dermato	2024	ence@Dir	602-615	42	Fingerprints are created by elev		https://doi.	https://www.sciencedirect.com/science/article/pii/S073					0738-081X				Not related to AI
123	Drug-repositioning opportunities for cancer therapy: novel molecular targets for known compounds	Hilson Andrade	02/04/2025	Roberto Würth and Stefar	Drug Discovery To	2016	ence@Dir	190-199	21	Drug repositioning is gaining inc		https://doi.	https://www.sciencedirect.com/science/article/pii/S135					1359-6446				Not related to AI
124	Multilayered rule-based expert system for diagnosing uveitis	Hilson Andrade	02/04/2025	A.M. Mutawa and Mariam	Artificial Intelligenc	2019	ence@Dir	101691	99	Uveitis is a condition caused by		https://doi.	https://www.sciencedirect.com/s			Rule-based system, U		0933-3657				Not related to AI
125	Biological effects of Bougainvillea glabra, Delonix regia, Lantana camara, and Platycladus orientalis extracts and their possible metabolomi	Hilson Andrade	02/04/2025	Reham M. Mostafa and M	Microbial Pathoger	2024	ence@Dir	106870	195	Plants are a treasure trove of bi		https://doi.	https://www.sciencedirect.com/s			Culex pipiens, Larvici		0882-4010				Not related to AI
126	G-quadruplex motifs are functionally conserved in cis-regulatory regions of pathogenic bacteria: An in-silico evaluation	Hilson Andrade	02/04/2025	Upalabdha Dey and Shar	Biochimie	2021	ence@Dir	40-51	184	The role of G-quadruplexes in th		https://doi.	https://www.sciencedirect.com/s			G-quadruplex, Regula		0300-9084				Not related to AI
127	Multidimensional futuristic approaches to address the pandemics beyond COVID-19	Hilson Andrade	02/04/2025	Shifa Bushra Kotwal and I	Heliyon	2023	ence@Dir	e17148	9	Globally, the impact of the coror		https://doi.	https://www.sciencedirect.com/s			SARS-CoV-2, COVID		2405-8440				Not related to AI
128	Inter-city movement pattern of notifiable infectious diseases in China: a social network analysis	Hilson Andrade	02/04/2025	Lin-Jie Yu and Peng-Sher	The Lancet Regior	2025	ence@Dir	101261	54	Summary		https://doi.	https://www.sciencedirect.com/s			Human mobility, Netw		2666-6065				Not related to AI
129	Biowarfare, bioterrorism and biocrime: A historical overview on microbial harmful applications	Hilson Andrade	02/04/2025	Manuela Oliveira and Gab	Forensic Science I	2020	ence@Dir	110366	314	Microbial Forensics is a field tha		https://doi.	https://www.sciencedirect.com/s			Biocrime, Bioterrorisn		0379-0738				Not related to AI
130	Spatial heterogeneity analysis for the transmission of syphilis disease in China via a data-validated reaction–diffusion model	Hilson Andrade	02/04/2025	Peng Wu and Xiunan War	Mathematical Bios	2024	ence@Dir	109243	375	Based on the distinctive spatial		https://doi.	https://www.sciencedirect.com/s			Syphilis, Spatial heter		0025-5564				Not related to AI
131	Gut microbiota in Alzheimer's disease: Understanding molecular pathways and potential therapeutic perspectives	Hilson Andrade	02/04/2025	Simone Lista and Antonio	Ageing Research I	2025	ence@Dir	102659	104	Accumulating evidence suggest		https://doi.	https://www.sciencedirect.com/s			Alzheimer's disease,		1568-1637				Not related to AI
132	Problems with the WHO TB model	Hilson Andrade	02/04/2025	Deborah Wallace and Roc	Mathematical Bios	2019	ence@Dir	71-80	313	WHO tuberculosis researchers r		https://doi.	https://www.sciencedirect.com/s			Tuberculosis, Endemi		0025-5564				Not related to AI
133	Identification of Berbamine, Oxyacanthine and Rutin from Berberis asiatica as anti-SARS-CoV-2 compounds: An in silico study	Hilson Andrade	02/04/2025	Tanuja Joshi and Sunaulla	Journal of Molecul	2021	ence@Dir	108028	109	Owing to the shortage of specifi		https://doi.	https://www.sciencedirect.com/s			SARS-CoV-2, , Mpro,		1093-3263				Surveys
134	Integration of network pharmacology, molecular docking, and simulations to evaluate phytochemicals from Drymaria cordata against cervic	Hilson Andrade	02/04/2025	Kunal Bhattacharya and E	RSC Advances	2024	ence@Dir	188-420	14	Introduction: Cervical cancer is		https://doi.	https://www.sciencedirect.com/science/article/pii/S204					2046-2069				Not related to AI
135	Natural bioactive products as promising therapeutics: A review of natural product-based drug development	Hilson Andrade	02/04/2025	Chetan B. Aware and Dev	South African Jour	2022	ence@Dir	512-528	151	Since ancient times, natural me		https://doi.	https://www.sciencedirect.com/s			Bioprospecting, Bioth		0254-6299		Biotechnological explora		Not related to AI
136	The Molecular Organization of Human cGMP Specific Phosphodiesterase 6 (PDE6): Structural Implications of Somatic Mutations in Cance	Hilson Andrade	02/04/2025	Arooma Maryam and Sun	Computational anc	2019	ence@Dir	378-389	17	In the cyclic guanosine monoph		https://doi.	https://www.sciencedirect.com/s			Somatic mutations, C		2001-0370				Not related to AI
137	Focused Cardiac Ultrasound to Guide the Diagnosis of Heart Failure in Pregnant Women in India	Hilson Andrade	02/04/2025	Maryam Alsharqi and Vija	Journal of the Ame	2022	ence@Dir	281-129	35	Background		https://doi.	https://www.sciencedirect.com/s			Focused cardiac ultra		0894-7317				Not related to AI
138	Animal models of pain: Diversity and benefits	Hilson Andrade	02/04/2025	Cynthia Abboud and Alexi	Journal of Neurosc	2021	ence@Dir	108997	348	Chronic pain is a maladaptive n		https://doi.	https://www.sciencedirect.com/s			Pain, Animal models,		0165-0270				Not related to AI
139	The oxidation of steroid derivatives by the CYP125A6 and CYP125A7 enzymes from Mycobacterium marinum	Hilson Andrade	02/04/2025	Amna Ghith and Stephen	The Journal of Ste	2023	ence@Dir	106406	235	The members of the bacterial cy		https://doi.	https://www.sciencedirect.com/s			Metalloenzymes, Ster		0960-0760				Not related to AI
140	Burden of disease scenarios for 204 countries and territories, 2022–2050: a forecasting analysis for the Global Burden of Disease Study 2(	Hilson Andrade	02/04/2025	Stein Emil Vollset and Ha	The Lancet	2024	ence@Dir	204-225	403	Summary		https://doi.	https://www.sciencedirect.com/science/article/pii/S014					0140-6736				Not related to AI
141	Genomic mapping of wastewater bacteriophage may predict potential bacterial pathogens infecting the community	Hilson Andrade	02/04/2025	Pankaj Bhatt and Yabing L	Science of The Tot	2024	ence@Dir	176834	955	Most existing wastewater survei		https://doi.	https://www.sciencedirect.com/s			Bacterial pathogen id		0048-9697				Not related to AI
142	Medicinal Chemistry Gone Wild	Hilson Andrade	02/04/2025	Tesko Chaganti and Chun	Journal of Medicin	2024	ence@Dir	899-690	67	Earth is currently experiencing a		https://doi.	https://www.sciencedirect.com/science/article/pii/S152					1520-4804				Not related to AI
143	Computational prediction of extracellular loops of the Por39 outer membrane porin of Rhodospirillum rubrum suitable for epitope surface di	Hilson Andrade	02/04/2025	Daniel Markthaler and Ro	Computational anc	2023	ence@Dir	483-249	21	Outer membrane porins from Gr		https://doi.	https://www.sciencedirect.com/s			Porins, Outer membra		2001-0370				Not related to AI
144	Platelet-derived bio-products: Classification update, applications, concerns and new perspectives	Hilson Andrade	02/04/2025	Andrea Acebes-Huerta an	Transfusion and Ap	2020	ence@Dir	102716	59	Platelet derived bio-products in		https://doi.	https://www.sciencedirect.com/s			Platelets, Platelet Ric		1473-0502				Not related to AI
145	Diagnosis and classification of optic neuritis	Hilson Andrade	02/04/2025	Axel Petzold and Clare L I	The Lancet Neurol	2022	ence@Dir	120-113	21	Summary		https://doi.	https://www.sciencedirect.com/science/article/pii/S147					1474-4422				Not related to AI
146	Drying kinetics, energy, statistical, economic, and proximate analysis of a greenhouse dryer using different glazing materials for Coccinia g	Hilson Andrade	02/04/2025	S. Rajesh and S. Sekar ar	Solar Energy	2024	ence@Dir	113047	284	In this study, ivy gourd (Coccinia		https://doi.	https://www.sciencedirect.com/s			Greenhouse dryer, D		0038-092X				Not related to leprosy
147	Molecular docking and simulation studies of Chloroquine, Rimantadine and CAP-1 as potential repurposed antivirals for decapod iridescen	Hilson Andrade	02/04/2025	Wan-Atirah Azemin and N	Fish and Shellfish	2023	ence@Dir	100120	5	Drug repurposing is a methodol		https://doi.	https://www.sciencedirect.com/s			Shrimp hemocyte irid		2667-0119				Not related to AI
148	Elevated neopterin in tuberculosis and co-infection with HIV and the effect of treatment: A systematic review, meta-analysis, and meta-regr	Hilson Andrade	02/04/2025	Amene Saghazadeh and I	International Immu	2022	ence@Dir	109147	111	Background		https://doi.	https://www.sciencedirect.com/s			Anti-tuberculosis trea		1567-5769				Not related to AI
149	Aquatic resource consumption at the Odense leprosarium: Advancing the limits of palaeodiet reconstruction with amino acid δ13C measure	Hilson Andrade	02/04/2025	Anastasia Brozou and Be	Journal of Archaec	2022	ence@Dir	105578	141	Dietary patterns of leprosy patie		https://doi.	https://www.sciencedirect.com/s			Amino acids, GC-IRM		0305-4403				Not related to AI
150	Protecting Human and Animal Health: The Road from Animal Models to New Approach Methods	Hilson Andrade	02/04/2025	Barbara L.F. Kaplan and A	Pharmacological F	2024	ence@Dir	251-266	76	Animals and animal models hav		https://doi.	https://www.sciencedirect.com/science/article/pii/S003					0031-6997				Not related to AI
151	Drug repurposing: From the discovery of a useful pharmacological effect to making the treatment available to the patient	Hilson Andrade	02/04/2025	Dominique Deplanque an	Therapies	2023	ence@Dir	10-18	78	Summary		https://doi.	https://www.sciencedirect.com/s			Repurposing, Marketi		0040-5957		GIENS WORKSHOPS (		Not related to leprosy
152	Health-adjusted life expectancy (HALE) in Chongqing, China, 2017: An artificial intelligence and big data method estimating the burden of c	Hilson Andrade	02/04/2025	Xiaowen Ruan and Yue Li	The Lancet Regior	2021	ence@Dir	100110	9	Background		https://doi.	https://www.sciencedirect.com/science/article/pii/S266					2666-6065				Not related to AI
153	Routes to molecular glue degrader discovery	Hilson Andrade	02/04/2025	Yanfen Liu and Jieyun Ba	Trends in Biochem	2025	ence@Direct			Molecular glue degraders (MGD		https://doi.	https://www.sciencedirect.com/s			targeted protein degra		0968-0004				Not related to AI
154	Cloud enabled data analytics and visualization framework for health-shocks prediction	Hilson Andrade	02/04/2025	Shahid Mahmud and Rah	Future Generation	2016	ence@Dir	169-181	65	In this paper, we present a data		https://doi.	https://www.sciencedirect.com/s			Technology integratio		0167-739X		Special Issue on Big Da		Not related to AI
155	Bi-PSSM: Position specific scoring matrix based intelligent computational model for identification of mycobacterial membrane proteins	Hilson Andrade	02/04/2025	Muslim Khan and Maqsoo	Journal of Theoret	2017	ence@Dir	116-124	435	Mycobacterium is a pathogenic		https://doi.	https://www.sciencedirect.com/s			SVM, PSSM, Mycoba		0022-5193				Not related to AI
156	OSADHI – An online structural and analytics based database for herbs of India	Hilson Andrade	02/04/2025	Kikrusenuo Kiewhuo and	Computational Bio	2023	ence@Dir	107799	102	The current study aims to devel		https://doi.	https://www.sciencedirect.com/s			Medicinal plants, Phy		1476-9271				Not related to leprosy
157	Exploration of innovative drug repurposing strategies for combating human protozoan diseases: Advances, challenges, and opportunities	Hilson Andrade	02/04/2025	ShanShan Hu and Zahra	Journal of Pharma	2025	ence@Dir	101084	15	Protozoan infections (e.g., mala		https://doi.	https://www.sciencedirect.com/s			Drug repurposing, Hu		2095-1779				Not related to AI
158	Machine Learning for Prediction of Tuberculosis Detection: Case Study of Trained African Giant Pouched Rats	Hilson Andrade	02/04/2025	Joan Jonathan and Alcard	Online Journal of F	2024	ence@Direct		16	Background		https://doi.	https://www.sciencedirect.com/s			machine learning, Afr		1947-2579				Not related to leprosy
159	A Systematic Review of Citrus Disease Perceptions and Fruit Grading Using Machine Vision	Hilson Andrade	02/04/2025	Shantilata Palei and Santi	Procedia Compute	2023	ence@Dir	504-251	218	Citrus is one of the most commc		https://doi.	https://www.sciencedirect.com/s			Citrus disease detecti		1877-0509		International Conference		Not related to AI
160	Geometrical and thermodynamic stability of govaniadine scaffold adducts with dopamine receptor D1	Hilson Andrade	02/04/2025	Ram {Lal Swagat Shresth	Results in Chemis	2024	ence@Dir	101363	7	Govaniadine, an isoquinoline all		https://doi.	https://www.sciencedirect.com/s			Free energy, MMGBS		2211-7156				Not related to leprosy
161	Global contrast-masked autoencoders are powerful pathological representation learners	Hilson Andrade	02/04/2025	Hao Quan and Xingyu Li a	Pattern Recognitio	2024	ence@Dir	110745	156	Using digital pathology slide sca		https://doi.	https://www.sciencedirect.com/s			Self-supervised learn		0031-3203				Not related to leprosy
162	High acknowledgement index: Characterizing research supporters with factors of acknowledgement affecting paper citation counts	Hilson Andrade	02/04/2025	Go Eun Heo and Young S	Journal of Informe	2023	ence@Dir	101447	17	The acknowledgments section c		https://doi.	https://www.sciencedirect.com/s			Acknowledgement typ		1751-1577				Not related to AI
163	Implementation of face recognition system using BioCryptosystem as template protection scheme	Hilson Andrade	02/04/2025	Alamgir Sardar and Saiye	Journal of Informa	2022	ence@Dir	103317	70	This paper has designed and im		https://doi.	https://www.sciencedirect.com/s			BioCryptosystem, Hu		2214-2126				Not related to AI
164	Back to the future: Old values for a new (more equal) world	Hilson Andrade	02/04/2025	Joel Millward-Hopkins	Futures	2021	ence@Dir	102727	128	Inequality has become a definin		https://doi.	https://www.sciencedirect.com/s			Anthropology, Egalita		0016-3287				Not related to AI
165	Diagnostic assessment of ulnar nerve thickness in leprosy using high-resolution ultrasonography versus magnetic resonance imaging	Hilson Andrade	02/04/2025	Bhavni Oberoi and Dev P	Medical Journal Ar	2024	ence@Direct			Background		https://doi.	https://www.sciencedirect.com/s			Leprosy, Ulnar nerve,		0377-1237				Not related to AI
166	Emerging Techniques in Spatial Multiomics: Fundamental Principles and Applications to Dermatology	Hilson Andrade	02/04/2025	Bojing B. Jia and Bryan K.	Journal of Investig	2024	ence@Direct			Molecular pathology, such as hi		https://doi.	https://www.sciencedirect.com/s			Dermatopathology, D		0022-202X				Not related to AI
167	Advancements of Raman spectroscopy in cosmetics and dermatology	Hilson Andrade	02/04/2025	Wilson Pearl Evangeline a	South African Jour	2024	ence@Dir	122-129	167	Raman spectroscopy, a powerfu		https://doi.	https://www.sciencedirect.com/s			Raman spectroscopy,		0254-6299				Not related to AI
168	Single-cell spatial transcriptomics in cardiovascular development, disease, and medicine	Hilson Andrade	02/04/2025	Songjie Han and Qianqiar	Genes & Diseases	2024	ence@Dir	101163	11	Cardiovascular diseases (CVDs		https://doi.	https://www.sciencedirect.com/s			Cardiovascular disea		2352-3042				Not related to AI
169	GCNGAT: Drug–disease association prediction based on graph convolution neural network and graph attention network	Hilson Andrade	02/04/2025	Runtao Yang and Yao Fu	Artificial Intelligenc	2024	ence@Dir	102805	150	Predicting drug–disease associa		https://doi.	https://www.sciencedirect.com/s			Drug–disease associ		0933-3657				Not related to leprosy
170	Engineering innovative interfaces for point-of-care diagnostics	Hilson Andrade	02/04/2025	Damon T. Burrow and Jac	Current Opinion in	2023	ence@Dir	101718	66	The ongoing Coronavirus diseas		https://doi.	https://www.sciencedirect.com/s			Point-of-care tests, d		1359-0294				Not related to leprosy
171	Retrosynthesis from transforms to predictive sustainable chemistry and nanotechnology: a brief tutorial review	Hilson Andrade	02/04/2025	Alicja Mikolajczyk and Ula	Green Chemistry	2023	ence@Dir	971-299	25	Retrosynthesis is a tool initially		https://doi.	https://www.sciencedirect.com/science/article/pii/S146					1463-9262				Not related to leprosy
172	Does your dermatology classifier know what it doesn't know? Detecting the long-tail of unseen conditions	Hilson Andrade	02/04/2025	Abhijit {Guha Roy} and Ji	Medical Image Ana	2022	ence@Dir	102274	75	Supervised deep learning mode		https://doi.	https://www.sciencedirect.com/s			Deep learning, Derm		1361-8415				Excluded by QA1
173	Automated chronic wounds medical assessment and tracking framework based on deep learning	Hilson Andrade	02/04/2025	Brayan Monroy and Karer	Computers in Biolc	2023	ence@Dir	107335	165	Chronic wounds are a latent hea		https://doi.	https://www.sciencedirect.com/s			Leprosy, Wound heali		0010-4825				Not related to leprosy
174	Emerging technologies for drug repurposing: Harnessing the potential of text and graph embedding approaches	Hilson Andrade	02/04/2025	Xialan Dong and Weifan Z	Artificial Intelligenc	2024	ence@Dir	100060	2	Drug repurposing is an approac		https://doi.	https://www.sciencedirect.com/s			Machine learning, Dr		2949-7477				Not related to leprosy
175	SLGCN: Structure-enhanced line graph convolutional network for predicting drug–disease associations	Hilson Andrade	02/04/2025	Bao-Min Liu and Ying-Liar	Knowledge-Based	2024	ence@Dir	111187	283	Drug repositioning is a rapidly g		https://doi.	https://www.sciencedirect.com/s			Drug–disease associ		0950-7051				Not related to AI
176	Applied Machine Learning Techniques to Diagnose Voice-Affecting Conditions and Disorders: Systematic Literature Review	Hilson Andrade	02/04/2025	Alper Idrisoglu and Ana L	Journal of Medical	2023	ence@Direct		25	Background		https://doi.	https://www.sciencedirect.com/s			diagnosis, digital bion		1438-8871				Not related to leprosy
177	Hypersymbiotics™: An artistic reflection on the ethical and environmental implications of microbiome research and new technologies	Hilson Andrade	02/04/2025	Anna Dumitriu	Endeavour	2022	ence@Dir	100820	46	This essay describes my ongoin		https://doi.	https://www.sciencedirect.com/s			BioArt, Art, Microbior		0160-9327		The Microbiome and its		Not related to AI
178	Semantic web-based diagnosis and treatment of vector-borne diseases using SWRL rules	Hilson Andrade	02/04/2025	Ritesh Chandra and Sadh	Knowledge-Based	2023	ence@Dir	110645	274	Vector-borne diseases (VBDs) a		https://doi.	https://www.sciencedirect.com/s			Semantic web, Decisi		0950-7051				Not related to AI
179	An ensemble approach for supporting the respiratory isolation of presumed tuberculosis inpatients	Hilson Andrade	02/04/2025	E.d.S. Alves and João B.C	Neurocomputing	2019	ence@Dir	289-300	331	Tuberculosis remains a global h		https://doi.	https://www.sciencedirect.com/s			Decision support syst		0925-2312				Not related to leprosy
180	M3S-ALG: Improved and robust prediction of allergenicity of chemical compounds by using a novel multi-step stacking strategy	Hilson Andrade	02/04/2025	Phasit Charoenkwan and	Future Generation	2025	ence@Dir	107455	162	A wide variety of chemicals can		https://doi.	https://www.sciencedirect.com/s			Chemical allergens, A		0167-739X				Not related to AI
181	Analysis of transcriptomic responses to SARS-CoV-2 reveals plausible defective pathways responsible for increased susceptibility to infect	Hilson Andrade	02/04/2025	Enrique J. deAndrés-Galia	Computers in Biolc	2022	ence@Dir	106029	149	Background		https://doi.	https://www.sciencedirect.com/s			, , , , ,		0010-4825				Not related to AI
182	Optimized real-time diagnosis of neglected tropical diseases by automatic recognition of skin lesions	Hilson Andrade	02/04/2025	Nyatte Steyve and Perabi	Informatics in Med	2022	ence@Dir	101078	33	Background		https://doi.	https://www.sciencedirect.com/s			Neglect tropical disea		2352-9148				Included
183	Metabolomics for the masses: The future of metabolomics in a personalized world	Hilson Andrade	02/04/2025	Drupad K. Trivedi and Kat	New Horizons in T	2017	ence@Dir	294-305	3	Current clinical practices focus		https://doi.	https://www.sciencedirect.com/science/article/pii/S230					2307-5023				Not related to AI
184	East-West beliefs challenged by North-South evidence about collectivism versus individualism	Hilson Andrade	02/04/2025	Evert {Van de Vliert} and E	Journal of Environ	2025	ence@Dir	102498	101	Psychologists inevitably study p		https://doi.	https://www.sciencedirect.com/s			Latitudinal psycholog		0272-4944				Not related to AI
185	Evaluation of 2 Artificial Intelligence Software for Chest X-Ray Screening and Pulmonary Tuberculosis Diagnosis: Protocol for a Retrospect	Hilson Andrade	02/04/2025	Muhammad Faiz {Mohd H	JMIR Research Pr	2023	ence@Direct		12	Background		https://doi.	https://www.sciencedirect.com/s			artificial intelligence, A		1929-0748				Not related to leprosy
186	Leprosy Screening Based on Artificial Intelligence: Development of a Cross-Platform App	Hilson Andrade	02/04/2025	Márcio Luís Moreira {De S	JMIR mHealth and	2021	ence@Direct		9	Background		https://doi.	https://www.sciencedirect.com/s			leprosy, artificial intell		2291-5222				Included
187	Contrastive knowledge integrated graph neural networks for Chinese medical text classification	Hilson Andrade	02/04/2025	Ge Lan and Mengting Hu	Engineering Applic	2023	ence@Dir	106057	122	This paper aims at medical text		https://doi.	https://www.sciencedirect.com/s			Knowledge graph, Gr		0952-1976				Not related to AI
188	Bioinformatics analysis characterizes immune infiltration landscape and identifies potential blood biomarkers for heart transplantation	Hilson Andrade	02/04/2025	Yujia Wang and Xiaoping	Transplant Immun	2024	ence@Dir	102036	84	Background: Cardiac allograft re		https://doi.	https://www.sciencedirect.com/s			Acute cardiac allogra		0966-3274				Not related to leprosy
189	Guideline for the diagnosis, treatment and long-term management of cutaneous lupus erythematosus	Hilson Andrade	02/04/2025	Qianjin Lu and Hai Long a	Journal of Autoimn	2021	ence@Dir	102707	123	Cutaneous lupus erythematosus		https://doi.	https://www.sciencedirect.com/s			Lupus erythematosus		0896-8411				Not related to AI
190	Ensemble and consensus approaches to prediction of recessive inheritance for missense variants in human disease	Hilson Andrade	02/04/2025	Ben O. Petrazzini and Da	Cell Reports Meth	2024	ence@Dir	100914	4	Summary		https://doi.	https://www.sciencedirect.com/s			mode of inheritance,		2667-2375				Not related to AI
191	Artificial intelligence in uveitis: A comprehensive review	Hilson Andrade	02/04/2025	Luis F. Nakayama and Lu	Survey of Ophthal	2023	ence@Dir	669-677	68	Uveitis is a disease complex ch		https://doi.	https://www.sciencedirect.com/s			Uveitis, Artificial intelli		0039-6257				Not related to leprosy
192	Analyzing the Predictability of an Artificial Intelligence App (Tibot) in the Diagnosis of Dermatological Conditions: A Cross-sectional Study	Hilson Andrade	02/04/2025	Shiva Shankar Marri and	JMIR Dermatology	2023	ence@Direct		6	Background		https://doi.	https://www.sciencedirect.com/s			artificial intelligence, A		2562-0959				Not related to leprosy
193	EfficientSkinDis: An EfficientNet-based classification model for a large manually curated dataset of 31 skin diseases	Hilson Andrade	02/04/2025	Abdul Rafay and Waqar H	Biomedical Signal	2023	ence@Dir	104869	85	Skin diseases are a common he		https://doi.	https://www.sciencedirect.com/s			Deep learning, Skin d		1746-8094				Included
194	Modern enabling techniques and adsorbents based dye removal with sustainability concerns in textile industrial sector -A comprehensive re	Hilson Andrade	02/04/2025	N. Rajesh Jesudoss Hyne	Journal of Cleaner	2020	ence@Dir	122636	272	Wastewaters from the textile ind		https://doi.	https://www.sciencedirect.com/s			Textile effluent, Dye a		0959-6526				Not related to AI
195	Novel Diagnostics for Kaposi Sarcoma and Other Skin Diseases in Resource-Limited Settings	Hilson Andrade	02/04/2025	Devon E. McMahon and L	Dermatologic Clini	2021	ence@Dir	83-90	39			https://doi.	https://www.sciencedirect.com/s			Point of care, Delaye		0733-8635		Global Dermatology and		Not related to AI
196	Eponyms that honor Jewish dermatologists: A celebration and a remembrance, Part three: Jewish physicians who practiced during the Hol	Hilson Andrade	02/04/2025	Leonard J. Hoenig and Da	Clinics in Dermato	2024	ence@Dir	299-312	42	Part III of this contribution contir		https://doi.	https://www.sciencedirect.com/science/article/pii/S073					0738-081X		Dermatology and Artifici		Not related to AI
197	Leveraging AI and Machine Learning to Develop and Evaluate a Contextualized User-Friendly Cough Audio Classifier for Detecting Respira	Hilson Andrade	02/04/2025	Kahabi Ganka Isangula ar	JMIR Research Pr	2024	ence@Direct		13	Background		https://doi.	https://www.sciencedirect.com/s			artificial intelligence, r		1929-0748				Not related to leprosy
198	Modeling the spread of infections during an epidemiological outbreak using an improved mathematical model	Hilson Andrade	02/04/2025	Nouf Abd Elmunim	Chaos, Solitons &	2024	ence@Dir	100111	12	Pandemics occur periodically w		https://doi.	https://www.sciencedirect.com/s			Pandemic, Covid-19,		2590-0544				Not related to leprosy
199	A step towards quantifying, modelling and exploring uncertainty in biomedical knowledge graphs	Hilson Andrade	02/04/2025	Adil Bahaj and Mounir Gh	Computers in Biolc	2025	ence@Dir	109355	184	Objective:		https://doi.	https://www.sciencedirect.com/s			Biomedical knowledg		0010-4825				Not related to leprosy
200	Seasonal variation of antibiotic resistance genes in activated sludge of a full-scale municipal wastewater treatment plant: Contribution of ac	Hilson Andrade	02/04/2025	Shashika Kumudumali Gu	Water Research	2025	ence@Dir	122598	268	It has been demonstrated that a		https://doi.	https://www.sciencedirect.com/s			Seasonal dynamic, M		0043-1354				Not related to leprosy

Id	title	data extractor	date of data extraction	author	journal	year	source	pages	volume	abstract	document_type	doi	url	affiliation	or_keywo	keywords	publisher	issn	nguag	note	lection_criter	Selection
201	What's new in IBD therapy: An "omics network" approach	Hilson Andrade	02/04/2025	Claudio Fiocchi and Dimit	Pharmacological F	2020	ence@Dir	104886	159	The industrial revolution that beg		https://doi.	https://www.sciencedirect.com/s			Inflammatory bowel d		1043-6618				Not related to leprosy
202	Associations between literacy and attitudes toward artificial intelligence–assisted medical consultations: The mediating role of perceived di	Hilson Andrade	02/04/2025	Enoch {Yi-No Kang} and D	Computers in Hum	2023	ence@Dir	107529	139	Artificial intelligence (AI) is incre		https://doi.	https://www.sciencedirect.com/s			Health literacy, Digita		0747-5632				Not related to leprosy
203	Structure-based drug repositioning: Potential and limits	Hilson Andrade	02/04/2025	Melissa F. Adasme and D	Seminars in Cance	2021	ence@Dir	192-198	68	Drug repositioning, the assignm		https://doi.	https://www.sciencedirect.com/s			Drug repositioning, S		1044-579X		Drug Repurposing for C		Not related to leprosy
204	The Many Hosts of Mycobacteria 9 (MHM9): A conference report	Hilson Andrade	02/04/2025	Abigail Marie Klever and H	Tuberculosis	2023	ence@Dir	102377	142	The Many Hosts of Mycobacteri		https://doi.	https://www.sciencedirect.com/science/article/pii/S147					1472-9792				Not related to AI
205	Insights into Computational Drug Repurposing for Neurodegenerative Disease	Hilson Andrade	02/04/2025	Manish D. Paranjpe and A	Trends in Pharmac	2019	ence@Dir	565-576	40	Computational drug repurposing		https://doi.	https://www.sciencedirect.com/s			artificial intelligence, r		0165-6147		Special Issue: Rise of M		Not related to leprosy
206	Potential Capacity of China's development assistance for health on neglected tropical diseases	Hilson Andrade	02/04/2025	Hong-Mei Li and Men-Bac	Acta Tropica	2022	ence@Dir	106245	226	Neglected tropical diseases (NT		https://doi.	https://www.sciencedirect.com/s			Neglected tropical dis		0001-706X				Not related to AI
207	Efficacy of an Artificial Intelligence App (Aysa) in Dermatological Diagnosis: Cross-Sectional Analysis	Hilson Andrade	02/04/2025	Shiva Shankar Marri and V	JMIR Dermatology	2024	ence@Direct		7	Background		https://doi.	https://www.sciencedirect.com/s			artificial intelligence, A		2562-0959				Not related to leprosy
208	Molecular modeling approaches for the discovery of adenosine A2B receptor antagonists: current status and future perspectives	Hilson Andrade	02/04/2025	Pran Kishore Deb and Bal	Drug Discovery To	2019	ence@Dir	354-186	24	Adenosine receptors (ARs) are		https://doi.	https://www.sciencedirect.com/science/article/pii/S135					1359-6446				Not related to AI
209	Utilizing mechatronic agilent gas chromatography to validate therapeutic efficacy of Combretum paniculatum against oxidative stress and ir	Hilson Andrade	02/04/2025	Ifeoma F. Chukwuma and	Heliyon	2024	ence@Dir	e36586	10	The quest for novel antioxidant a		https://doi.	https://www.sciencedirect.com/s			Artificial intelligence, I		2405-8440				Not related to leprosy
210	Bringing Lab to the Field: Exploring Innovations in Point-of-Care Diagnostics for the Rapid Detection and Management of Tropical Diseases	Hilson Andrade	02/04/2025	Abdullahi Tunde Aborode	Advances in Bioma	2025	ence@Direct			Tropical diseases present major		https://doi.	https://www.sciencedirect.com/s			Point-of-Care Diagno		2543-1064				Surveys
211	Drugs from drugs: New chemical insights into a mature concept	Hilson Andrade	02/04/2025	Eloy Lozano Baró and Fe	Drug Discovery To	2024	ence@Dir	104212	29	Developing new drugs from mar		https://doi.	https://www.sciencedirect.com/s			drug discovery, medic		1359-6446				Not related to leprosy
212	Big Data, Big Decisions for Science, Society, and Business: Report on a Research Agenda Setting Workshop	Hilson Andrade	02/04/2025	Markus, M. Lynne and Topi, Heikki		2015	l Digital Library			The report from the workshop, "Big Data, Big Decisions for Government, Business and							National Science Foundation					Not related to leprosy
213	A Survey on Contact Tracing: The Latest Advancements and Challenges	Hilson Andrade	02/04/2025	Jiang, Ting and Zhang, Ya	ACM Trans. Spatia	2022	l Digital Library		8	Infectious diseases are caused		10.1145/3	https://doi.org/10.1145/3494529			privacy iss	Associatio	2374-0353				Not related to AI
214	CHI '21: Proceedings of the 2021 CHI Conference on Human Factors in Computing Systems	Hilson Andrade	02/04/2025			2021	l Digital Library										Association for Computing Machinery					Not related to AI
215	WWW '19: Companion Proceedings of The 2019 World Wide Web Conference	Hilson Andrade	02/04/2025			2019	l Digital Library			It is our great pleasure to welcome you to <I>The Web Conference 2019</I>.							Association for Computing Machinery					Not related to leprosy
216	Let's agree to disagree: on the evaluation of vocabulary alignment	Hilson Andrade	02/04/2025	Tordai, Anna and van Ossenbruggen, Jacco a		2011	l Digital Lit	65–72		Gold standard mappings create		10.1145/1	https://doi.org/10.1145/1999676.			vocabulary	Association for Computing Machinery					Not related to leprosy
217	Compact Walks: Taming Knowledge-Graph Embeddings with Domain- and Task-Specific Pathways	Hilson Andrade	02/04/2025	Hou, Pei-Yu and Korn, Daniel R. and Melo-Fi		2022	l Digital Lit	458–469		Knowledge-graph (KG) embedd		10.1145/3	https://doi.org/10.1145/3514221.			KG embec	Association for Computing Machinery					Not related to leprosy
218	Formal Concept Analysis Applications in Bioinformatics	Hilson Andrade	02/04/2025	Roscoe, Sarah and Khatri	ACM Comput. Sur	2022	l Digital Library		55	The bioinformatics discipline see		10.1145/3	https://doi.org/10.1145/3554728			protein-prc	Associatio	0360-0300				Not related to leprosy
219	Detection of Acid-Fast Bacilli AFB in Slit-Skin Smear Microscopy using MASK R-CNN Algorithm	Hilson Andrade	02/04/2025	Cabunilas, Joshua John L. and Ponio, Kean		2023	l Digital Lit	30–36		This study focuses on the devel		10.1145/3	https://doi.org/10.1145/3620679.			Acid Fast I	Association for Computing Machinery					Excluded by QA3
220	onyxRay: A Mobile-Based Nail Diseases Detection Using Custom Vision Machine Learning	Hilson Andrade	02/04/2025	Pinoliad, Sholomon L. and Dichoso, Duanne		2020	l Digital Lit	126–133		Nails serves as a great way to d		10.1145/3	https://doi.org/10.1145/3411681.			Systemic I	Association for Computing Machinery					Not related to leprosy
221	Offspring GAN augments biased human genomic data	Hilson Andrade	02/04/2025	Das, Supratim and Shi, Xinghua		2022	l Digital Library			Genomic data have been used f		10.1145/3	https://doi.org/10.1145/3535508.			mode colla	Association for Computing Machinery					Not related to leprosy
222	Genetics as a novel tool in mining impact assessment and biomonitoring of critically endangered western chimpanzees in the Nimba Moun	Hilson Andrade	02/04/2025	Koops, Kathelijne and Hu	Conservation Scie	2023	Scopus		5	Western c	Article	10.1111/cs	https://www.scopus.c		conservation management; environmental impact ass					Cited by: 2; All Open Acc		Not related to AI
223	Recent advances in point-of-care biosensors for the diagnosis of neglected tropical diseases	Hilson Andrade	02/04/2025	Deroco, Patricia Batista a	Sensors and Actua	2021	Scopus		349	Consideri	Article	10.1016/j.s	https://www.scopus.c		Electroche	Biosensors; Developing countries; Disease				Cited by: 14		Not related to AI
224	Simulated multimodal deep facial diagnosis	Hilson Andrade	02/04/2025	Jin, Bo and Gonçalves, N	Expert Systems wi	2024	Scopus		252	Facial phe	Article	10.1016/j.e	https://www.scopus.c		Bilinear; C	Computer aided diagnosis; Deep learning;				Cited by: 8; All Open Acc		Not related to leprosy
225	Evolutionary game analysis of coal enterprise resource integration under government regulation	Hilson Andrade	02/04/2025	Sun, Ziyuan and Wang, W	Environmental Sci	2022	Scopus	27 – 71	29	Resource	Article	10.1007/s1	https://www.scopus.c		Enterprise	China; Coal; Environmental Pollution; Gan				Cited by: 11		Not related to leprosy
226	Are leprosy and Hansen's disease identical?	Hilson Andrade	02/04/2025	Guo, Jie and Dong, Xiaob	Nature	2023	Scopus	31	622		Letter	10.1038/d	https://www.scopus.c		Diseases;	Humans; Leprosy; artificial intelligence; dis				Cited by: 0		Not related to AI
227	Emotion AI Use in U.S. Mental Healthcare: Potentially Unjust and Techno-Solutionist	Hilson Andrade	02/04/2025	Roemmich, Kat and Corvi	Proceedings of the	2024	Scopus		8	Emotion A	Article	10.1145/3	https://www.scopus.c		data subje	Behavioral research; Emotion Recognition				Cited by: 1; All Open Acc		Not related to leprosy
228	A wearable, self-sustainable, and wireless plantar pressure and temperature monitoring system for foot ulceration prognosis and rehabilitat	Hilson Andrade	02/04/2025	Pradhan, Gagan Bahadur	Sensors and Actua	2024	Scopus		379	Continuou	Article	10.1016/j.s	https://www.scopus.c		Diabetic fo	Arthroplasty; Dermatology; Diseases; Hosi				Cited by: 1		Not related to AI
229	Detection and Visualization of Neglected Tropical Skin Diseases Using EfficientNet and Grad-CAM	Hilson Andrade	02/04/2025	Surasinghe, Pabasara and	ICAC 2023 - 5th Ir	2023	Scopus	72 – 477		Early skin	Conference paper	10.1109/IC	https://www.scopus.c		EfficientNe	Automation; Cams; Dermatology; Tropics;				Cited by: 0		Included
230	Proceedings from the CIHLMU occupational safety and health symposium 2019 "Protecting workers' health: global challenges and opportu	Hilson Andrade	02/04/2025	Netsanet Workneh GidiAnna SurayaBeatrice		2020	pringer Link					10.1186/s1	http://link.springer.com/article/10.1186/s129				BMC Proceedings					Duplicated
231	Abstracts	Hilson Andrade	02/04/2025	unknown		2019	pringer Link					10.1007/s1	http://link.springer.com/article/10.1007/s120				Hepatology International					Duplicated
232	Using machine learning methods to detect physical conditions with postural balance	Hilson Andrade	02/04/2025	Chinmei ChouShih-Hsiung ChouYu Che Che		2023	pringer Link					10.1007/s1	http://link.springer.com/article/10.1007/s126				Journal of Ambient Intelligence and Humanized Computi					Duplicated
233	Multimodal AI/ML for discovering novel biomarkers and predicting disease using multi-omics profiles of patients with cardiovascular disease	Hilson Andrade	02/04/2025	William DeGroatHabiba AbdelhalimElizabeth		2024	pringer Link					10.1038/s	http://link.springer.com/article/10.1038/s415				Scientific Reports					Duplicated
234	Study of machine learning techniques for outcome assessment of leptospirosis patients	Hilson Andrade	02/04/2025	Andreia Ferreira da SilvaKarla Figueiredolgo		2024	pringer Link					10.1038/s	http://link.springer.com/article/10.1038/s415				Scientific Reports					Duplicated
235	Prediction of rifampicin resistance beyond the RRDR using structure-based machine learning approaches	Hilson Andrade	02/04/2025	Stephanie PortelliYoochan MyungNicholas F		2020	pringer Link					10.1038/s	http://link.springer.com/article/10.1038/s415				Scientific Reports					Duplicated
236	Heuristic Segmentation Assisted Deep-Spatial Feature Learning Model for Leprosy Detection	Hilson Andrade	02/04/2025	Jitendra, R. and Simha, J	SN Computer Scie	2024	Scopus		5	The high	Article	10.1007/s	https://www.scopus.c		AlexNet; Ensemble classification; GLCM; Heuristic-ba					Cited by: 0		Duplicated
237	AI Driven Edge Device for Screening Skin Lesion and Its Severity in Peripheral Communities	Hilson Andrade	02/04/2025	Jaikishore, Chathura.N. a	Proceedings of the	2021	Scopus			It is vital t	Conference paper	10.1109/IN	https://www.scopus.c		AI driven e	Convolutional neural networks; Diagnosis;				Cited by: 6		Duplicated
238	Pharmacological profiles of neglected tropical disease drugs	Hilson Andrade	02/04/2025	Greco, Alessandro and Ka	Artificial Intelligenc	2024	Scopus		6	According	Article	10.1016/j.a	https://www.scopus.c		Drug disco	acoziborole; albendazole; amphotericin B;				Cited by: 0; All Open Acc		Duplicated
239	Aquatic resource consumption at the Odense leprosarium: Advancing the limits of palaeodiet reconstruction with amino acid δ13C measure	Hilson Andrade	02/04/2025	Brozou, Anastasia and Fu	Journal of Archaeo	2022	Scopus		141	Dietary pa	Article	10.1016/j.j	https://www.scopus.c		Amino aci	Denmark; amino acid; archaeology; bone;				Cited by: 5		Duplicated
240	Deep Semantic Segmentation Assisted Region-of-Interest Sensitive Deep Spatio-Textural Feature Learning Framework for Leprosy Detect	Hilson Andrade	02/04/2025	Jitendra, R. and Simha, J	SN Computer Scie	2024	Scopus		5	The last f	Article	10.1007/s	https://www.scopus.c		CAD; Deep spatio-textural feature; Ensemble learning					Cited by: 0		Duplicated
241	Reimagining leprosy elimination with AI analysis of a combination of skin lesion images with demographic and clinical data	Hilson Andrade	02/04/2025	Barbieri, Raquel R and Xu	The Lancet Regior	2022	PubMed		9								Elsevier					Duplicated
242	Efficacy of an artificial intelligence App (Aysa) in dermatological diagnosis: cross-sectional analysis	Hilson Andrade	02/04/2025	Marri, Shiva Shankar and	JMIR dermatology	2024	PubMed	e48811	7								JMIR Publications Inc., Toronto, Canada					Duplicated
243	Deep learning for AI-based diagnosis of skin-related neglected tropical diseases: A pilot study	Hilson Andrade	02/04/2025	Yotsu, Rie R and Ding, Zh	PLOS Neglected T	2023	PubMed	001123	17								Public Library of Science San Francisco, CA USA					Duplicated
244	Analyzing the Predictability of an artificial intelligence app (Tibot) in the diagnosis of dermatological conditions: A cross-sectional study	Hilson Andrade	02/04/2025	Marri, Shiva Shankar and	JMIR dermatology	2023	PubMed	e45529	6								JMIR Publications Inc., Toronto, Canada					Duplicated
245	Blood RNA signature RISK4LEP predicts leprosy years before clinical onset	Hilson Andrade	02/04/2025	Ti{\'o}-Coma, Maria and K	EBioMedicine	2021	PubMed		68								Elsevier					Duplicated
246	Evaluation of 2 Artificial Intelligence Software for Chest X-Ray Screening and Pulmonary Tuberculosis Diagnosis: Protocol for a Retrospect	Hilson Andrade	02/04/2025	Hisham, Muhammad Faiz	JMIR Research Pr	2023	PubMed	e36121	12								JMIR Publications Inc., Toronto, Canada					Duplicated
247	Automated chronic wounds medical assessment and tracking framework based on deep learning	Hilson Andrade	02/04/2025	Monroy, Brayan and Sanc	Computers in Biolo	2023	PubMed	107335	165								Elsevier					Duplicated
248	Insights into computational drug repurposing for neurodegenerative disease	Hilson Andrade	02/04/2025	Paranjpe, Manish D and T	Trends in pharmac	2019	PubMed	565--576	40								Elsevier					Duplicated
249	Leprosy screening based on artificial intelligence: Development of a cross-platform app	Hilson Andrade	02/04/2025	De Souza, M{\'a}rcio Lu{\'	JMIR mHealth and	2021	PubMed	e23718	9								JMIR Publications Inc., Toronto, Canada					Duplicated
250	A hand biomechanics workstation	Hilson Andrade	02/04/2025	Thompson, David E. and Buford, William L. a		1988	l Digital Lit	335–343		Interactive graphics for hand sur		10.1145/5	https://doi.org/10.1145/54852.37			orthopedic	Association for Computing Machinery					Duplicated
251	GC-MS profiling of Bauhinia variegata major phytoconstituents with computational identification of potential lead inhibitors of SARS-CoV-2	Hilson Andrade	02/04/2025	Pallavi More-Adate and Ki	Computers in Biolo	2022	ence@Dir	105679	147	Severe acute respiratory syndro		https://doi.	https://www.sciencedirect.com/s			, , , , , ,		0010-4825				Not related to leprosy
252	Identifying novel aryl hydrocarbon receptor (AhR) modulators from clinically approved drugs: In silico screening and In vitro validation	Hilson Andrade	02/04/2025	Farag E.S. Mosa and Mo	Archives of Bioche	2024	ence@Dir	109958	754	The aryl hydrocarbon receptor (		https://doi.	https://www.sciencedirect.com/s			Aryl hydrocarbon rec		0003-9861				Not related to AI
253	Mobile Health Strategies to Tackle Skin Neglected Tropical Diseases With Recommendations From Innovative Experiences: Systematic Re	Hilson Andrade	02/04/2025	Carme Carrion and Noem	JMIR mHealth and	2020	ence@Direct		8	Background		https://doi.	https://www.sciencedirect.com/s			mHealth, mobile heal		2291-5222				Surveys
254	Point-of-Care Ultrasound in Resource-Limited Settings	Hilson Andrade	02/04/2025	Michelle Beam and Shaik	Medical Clinics of	2025	ence@Dir	313-324	109			https://doi.	https://www.sciencedirect.com/s			Point-of-care ultrasou		0025-7125		Point-of-Care Ultrasoun		Not related to AI
255	Validation of a universal and highly sensitive two-dimensional liquid chromatography–tandem mass spectrometry methodology for the quar	Hilson Andrade	02/04/2025	Shengyuan Wu and Xiany	Journal of Chroma	2020	ence@Dir	122141	1151	A novel and potent anti-tubercul		https://doi.	https://www.sciencedirect.com/s			Anti-tuberculosis drug		1570-0232				Not related to AI
256	Diagnosis of Leprosy through AI-based Mobile Application	Hilson Andrade	02/04/2025	Nikam, Atharva and Rana	2022 OPJU Intern	2023	Scopus			No digital	Conference paper	10.1109/O	https://www.scopus.c		Artificial In	Dermatology; Diseases; Human computer				Cited by: 0		Duplicated
257	Anomaly detection of bridge health monitoring data based on KNN algorithm	Hilson Andrade	02/04/2025	Lei, Zhen and Zhu, Liang	Journal of Intellige	2020	Scopus	43 – 52	39	Pattern re	Article	10.3233/JI	https://www.scopus.c		Artificial in	Covariance matrix; Health; Monitoring; Ne				Cited by: 12		Not related to leprosy
258	Social marketing applications in public policy programs: key learnings and applications	Hilson Andrade	02/04/2025	Mishra, Satyam and Rishi	Social Responsibil	2021	Scopus	41 – 75	17	Purpose:	Article	10.1108/SI	https://www.scopus.c		Marketing programs; Public policy; Social impact; Soc					Cited by: 2		Not related to leprosy
259	Diversity of Mycobacterium tuberculosis in the Middle Fly District of Western Province, Papua New Guinea: microbead-based spoligotyping	Hilson Andrade	02/04/2025	Guernier-Cambert, Vanina	Scientific Reports	2019	Scopus		9	Tuberculo	Article	10.1038/s	https://www.scopus.com/inward/		Adult; DNA, Bacterial; Female; Genetic Va					Cited by: 5; All Open Acc		Not related to leprosy
260	Functional Annotation and Identification of Putative Drug Target in VV	Hilson Andrade	02/04/2025	Singh, Yashbir and Deepa	2020 3rd Internatio	2020	Scopus			Recent pr	Conference paper	10.1109/IC	https://www.scopus.c		Domain as	Computer viruses; Machinery; Proteins; Se				Cited by: 0		Not related to AI
261	COVID-19 Response and Preparedness in the Okayama Prefecture	Hilson Andrade	02/04/2025	Sodeno, Miho and Noriya	Journal of Disaster	2022	Scopus	87 – 92	17	In this arti	Article	10.20965/j	https://www.scopus.c		COVID-19	Artificial intelligence; Administration policy;				Cited by: 0; All Open Acc		Not related to leprosy
262	Ensemble optimization algorithm for the prediction of melanoma skin cancer	Hilson Andrade	02/04/2025	Gupta, Sachin and R, Jay	Measurement: Ser	2023	Scopus		29	One of the	Article	10.1016/j.r	https://www.scopus.c		Augmenta	Dermatology; Diagnosis; Diseases; Oncolo				Cited by: 10; All Open A		Not related to leprosy
263	Dynamical analysis and design of computational methods for nonlinear stochastic leprosy epidemic model	Hilson Andrade	02/04/2025	Raza, Ali and Awrejcewicz	Alexandria Engine	2022	Scopus	97 – 81	61	This articl	Article	10.1016/j.a	https://www.scopus.c		Computati	Computational methods; Cost effectivenes				Cited by: 13; All Open A		Not related to AI
264	Distribution and influencing factors of Mycobacterium in rail transit based on metagenomic	Hilson Andrade	02/04/2025	Liu, Yongping and Tong, L	Journal of Aerosol	2024	Scopus		179	With the i	Article	10.1016/j.j	https://www.scopus.c		Environme	Aerosols; Carbon dioxide; Pollution contro				Cited by: 0		Not related to AI
265	Integrated AI Medical Emergency Diagnostics Advising System	Hilson Andrade	02/04/2025	Aityan, Sergey K. and Mo	Electronics (Switze	2024	Scopus		13	The applic	Article	10.3390/el	https://www.scopus.c		AI; artificial intelligence; Internet of Things; IoT; large					Cited by: 0; All Open Acc		Not related to leprosy
266	Unified Spatial Clustering of Territory Risk to Uncover Impact of COVID-19 Pandemic on Major Coverages of Auto Insurance	Hilson Andrade	02/04/2025	Xie, Shengkun and Ho, N	Risks	2024	Scopus		12	This resea	Article	10.3390/ri	https://www.scopus.c		COVID-19 pandemic; insurance rate regulation; predi					Cited by: 0; All Open Acc		Not related to leprosy
267	Pathologic classification of a late-onset peripheral neuropathy in a spontaneous Labrador retriever dog model	Hilson Andrade	02/04/2025	Anderson, Ryan S. and C	Journal of Compar	2024	Scopus		532	Late-onse	Article	10.1002/c	https://www.scopus.c		axonopath	Animals; Axons; Dogs; Electromyography;				Cited by: 0; All Open Acc		Not related to leprosy
268	Bio-Inspired Modelling of Disease Through Delayed Strategies	Hilson Andrade	02/04/2025	Nasir, Arooj and Baleanu,	Computers, Materi	2022	Scopus	17 – 57	73	In 2020, t	Article	10.32604/	https://www.scopus.c		computer	Biomimetics; COVID-19; Lyapunov functio				Cited by: 0; All Open Acc		Excluded by QA1
269	Diagnosis of Ear Conditions Using Deep Learning Approach	Hilson Andrade	02/04/2025	Singh, Akriti and Dutta, M	ICCISc 2021 - 202	2021	Scopus			The lack c	Conference paper	10.1109/IC	https://www.scopus.c		Chronic O	Diagnosis; Medical imaging; Artificial intelli				Cited by: 4		Not related to leprosy
270	Influence of Embedded Microprocessor Wireless Communication in the Ankle Joint Proprioception Training on the Prevention of Football S	Hilson Andrade	02/04/2025	Li, Siwen	Mobile Information	2022	Scopus		2022	This study	Article	10.1155/2	https://www.scopus.com/inward/			Computation theory; Controllers; Infill drilli				Cited by: 1; All Open Acc		Not related to leprosy
271	Label-free macrophage phenotype classification using machine learning methods	Hilson Andrade	02/04/2025	Hourani, Tetiana and Pere	Scientific Reports	2023	Scopus		13	Macropha	Article	10.1038/s	https://www.scopus.com/inward/			Machine Learning; Macrophages; Phenoty				Cited by: 11; All Open A		Not related to leprosy
272	Human migration, infectious diseases, plague, global health crisis - historical evidence	Hilson Andrade	02/04/2025	Kinasih, Sri Endah and De	Cogent Arts and H	2024	Scopus		11	This study	Article	10.1080/2	https://www.scopus.c		Anthropology - Soc Sci; Disease Transmission; Healt					Cited by: 1; All Open Acc		Not related to AI
273	Improved predictive sparse decomposition method with densenet for prediction of lung cancer	Hilson Andrade	02/04/2025	Mienye, Ibomoiye Domor	International Jourr	2020	Scopus	33 – 54	19	Lung canc	Article	10.47839/i	https://www.scopus.c		convolutional neural network; deep learning; denseNe					Cited by: 24; All Open A		Not related to leprosy
274	Extraction of Relation Between Attributes and Class in Breast Cancer Data Using Rule Mining Techniques	Hilson Andrade	02/04/2025	Mohan, Krishna and Nair,	Advances in Intelli	2021	Scopus	42 – 35	1199	Breast ca	Conference paper	10.1007/9	https://www.scopus.c		Apriori alg	Data mining; Decision trees; Diagnosis; In				Cited by: 1		Not related to leprosy
275	Multi-objective periodic maintenance scheduling and optimization via krill herd algorithm	Hilson Andrade	02/04/2025	Aungkulanon, Pasura and	Songklanakarin Jo	2021	Scopus	42 – 13	43	This study	Article		https://www.scopus.c		Desirability function approaches; Modified krill herd al					Cited by: 3		Not related to leprosy
276	Analyzing and Visualizing Knowledge Structures of Research and Development Trends in Internet of Things for Smart Agriculture: A Decad	Hilson Andrade	02/04/2025	Maulana, Fairuz Iqbal and	E3S Web of Confe	2024	Scopus		501	Internet o	Conference paper	10.1051/e	https://www.scopus.com/inward/record.uri?eid=2-s2.0-85189247110&doi=1							Cited by: 3		Not related to leprosy
277	Effect of Jacobson's Progressive Muscle Relaxation on Anxiety and Happiness of Older Adults in the Nursing Home	Hilson Andrade	02/04/2025	Tapeh, Zahra Asgari and D	Iranian Journal of	2024	Scopus	78 – 84	29	Backgrou	Article	10.4103/ijr	https://www.scopus.c		Aged; anx	adult; aged; anxiety; article; autogenic trair				Cited by: 0; All Open Acc		Not related to leprosy
278	Skin Diseases Detection Using LBP and WLD: An Ensembling Approach	Hilson Andrade	02/04/2025	Banerjee, Arnab and Sark	SN Computer Scie	2024	Scopus		5	In all deve	Article	10.1007/s	https://www.scopus.c		LBP; Leprosy; Multi-SVM; Pattern recognition; STM;					Cited by: 1		Included
279	Perceived stigma and erotic technology: From sex toys to erobots	Hilson Andrade	02/04/2025	Dubé, S. and Santaguida,	Psychology and S	2023	Scopus	41 – 15	14	The inters	Article	10.1080/1	https://www.scopus.c		erobotics; erobots; Perceived stigma; sex toys; willing					Cited by: 10		Not related to leprosy
280	Study of machine learning techniques for outcome assessment of leptospirosis patients	Hilson Andrade	02/04/2025	Silva, Andreia Ferreira da	Scientific Reports	2024	Scopus		14	Leptospir	Article	10.1038/s	https://www.scopus.c		Data minir	Adult; Algorithms; Brazil; Decision Trees; F				Cited by: 0; All Open Acc		Not related to leprosy
281	Conflicts surrounding individual and collective aspects of ethics in health emergencies	Hilson Andrade	02/04/2025	Osorio-de-Castro, Claudia	Ethics and Behavi	2022	Scopus	18 – 63	32	Disasters	Article	10.1080/1	https://www.scopus.c		decision m	adult; article; controlled study; decision ma				Cited by: 0		Not related to AI
282	Single-nucleus cross-tissue molecular reference maps toward understanding disease gene function	Hilson Andrade	02/04/2025	Eraslan, Gökcen and Drok	Science	2022	Scopus		376	Understar	Article	10.1126/sc	https://www.scopus.com/inward/			Biomarkers; Cell Nucleus; Genome-Wide				Cited by: 166; All Open		Not related to AI
283	A Shared-Frailty Spatial Scan Statistic Model for Time-to-Event Data	Hilson Andrade	02/04/2025	Frévent, Camille and Ahm	Biometrical Journa	2024	Scopus		66	Spatial sc	Article	10.1002/b	https://www.scopus.c		conditiona	Biometry; Frailty; Humans; Kidney Failure,				Cited by: 0; All Open Acc		Not related to leprosy
284	Optimization of Coronavirus Pandemic Model Through Artificial Intelligence	Hilson Andrade	02/04/2025	Alqarni, Manal M. and Na	Computers, Materi	2023	Scopus	07 – 68	74	Artificial ir	Article	10.32604/	https://www.scopus.c		analysis; a	Errors; Intelligent agents; Mean square err				Cited by: 0; All Open Acc		Not related to leprosy
285	Performance Analysis for Trichoscopy and Trichogram Using Deep Learning and Image Processing—A Survey	Hilson Andrade	02/04/2025	Jain, Divyanshu and Mas	Lecture Notes in N	2024	Scopus	17 – 42	879	Hair-relate	Conference paper	10.1007/9	https://www.scopus.c		Deep lear	Diagnosis; Image analysis; Learning syste				Cited by: 0		Surveys
286	Explainable drug repurposing via path based knowledge graph completion	Hilson Andrade	02/04/2025	Jiménez, Ana and Merino,	Scientific Reports	2024	Scopus		14	Drug repu	Article	10.1038/s	https://www.scopus.c		Drug repu	Algorithms; Artificial Intelligence; Drug Rep				Cited by: 0; All Open Acc		Not related to leprosy
287	An Archaeology of Microbes	Hilson Andrade	02/04/2025	Warinner, Christina	Journal of Anthrop	2022	Scopus	20 – 45	78	Humans	Article	10.1086/7	https://www.scopus.c		ancient DNA; archaeological science; bacteria; ferme					Cited by: 2		Not related to leprosy
288	THE IMPACT OF PRIVACY CALCULUS AND TRUST ON USER INFORMATION PARTICIPATION BEHAVIOR IN AI-BASED MEDICAL CC	Hilson Andrade	02/04/2025	Luo, Yumei and Li, Xiaoqi	Journal of Electror	2023	Scopus	48 – 67	24	Artificial ir	Article		https://www.scopus.c		AI-based medical consultation; Gender; Information p					Cited by: 4		Not related to leprosy
289	How does energy utilization affect rural sustainability development in traditional villages? Re-examination from the coupling coordination de	Hilson Andrade	02/04/2025	Zhong, Qikang and Fu, H	Building and Envir	2024	Scopus		257	In the fac	Article	10.1016/j.b	https://www.scopus.c		Atmosphe	China; Hunan; Carbon; Ecology; Environm				Cited by: 20		Not related to leprosy
290	Leprosy Diagnosis using Explainable Artificial Intelligence Techniques	Hilson Andrade	02/04/2025	Baweja, Asis Kaur and Ad	2nd International C	2023	Scopus	51 – 556		LRprosy,	Conference paper	10.1109/IC	https://www.scopus.c		Convolutic	Chemical activation; Convolutional neural				Cited by: 3		Duplicated
291	Detection of Acid-Fast Bacilli AFB in Slit-Skin Smear Microscopy using MASK R-CNN Algorithm	Hilson Andrade	02/04/2025	Cabunilas, Joshua John L	ACM International	2023	Scopus	30 – 36		This study	Conference paper	10.1145/3	https://www.scopus.c		Acid Fast I	Convolutional neural networks; Diseases;				Cited by: 0		Duplicated
292	Raman spectroscopy and mass spectrometry identifies a unique group of epidermal lipids in active discoid lupus erythematosus	Hilson Andrade	02/04/2025	Holtkamp, Hannah U and	Scientific Reports	2023	Scopus		13	Discoid lu	Article	10.1038/s	https://www.scopus.com/inward/			Epidermis; Humans; Lipids; Lupus Eryther				Cited by: 1; All Open Acc		Not related to leprosy
293	Discrete time dynamics of a SIRD reinfection model	Hilson Andrade	02/04/2025	Eshmatov, F.F. and Jamilo	International Jourr	2023	Scopus		16	This pape	Article	10.1142/S	https://www.scopus.c		Modeling; quadratic stochastic operator; regular oper					Cited by: 4		Not related to AI
294	A BAYESIAN MODEL OF UNDERREPORTING FOR SEXUAL ASSAULT ON COLLEGE CAMPUSES	Hilson Andrade	02/04/2025	Bradshaw, By Casey and	Annals of Applied	2024	Scopus	46 – 31	18	In an effor	Article	10.1214/2	https://www.scopus.c		Bayesian hierarchical model; count data; Underreport					Cited by: 1		Not related to AI
295	Learning about COVID-19-related stigma, quarantine and isolation experiences in Finland	Hilson Andrade	02/04/2025	Lohiniva, Anna-Leena and	PLoS ONE	2021	Scopus		16	Backgrou	Article	10.1371/jo	https://www.scopus.com/inward/			Adult; Anxiety; Coronavirus Infections; CO				Cited by: 54; All Open A		Not related to leprosy
296	Research hotspots and trends on spinal cord stimulation for pain treatment: a two-decade bibliometric analysis	Hilson Andrade	02/04/2025	Yang, Sheng and Zhong,	Frontiers in Neuro	2023	Scopus		17	Backgrou	Article	10.3389/fr	https://www.scopus.c		bibliometri	Article; bibliometrics; chronic pain; clinical				Cited by: 3; All Open Acc		Surveys
297	Vision transformer based classification of gliomas from histopathological images	Hilson Andrade	02/04/2025	Goceri, Evgin	Expert Systems wi	2024	Scopus		241	Early and	Article	10.1016/j.e	https://www.scopus.c		Brain tumo	Convolution; Deep learning; Diagnosis; E-l				Cited by: 26		Not related to leprosy
298	Morphological cell profiling of SARS-CoV-2 infection identifies drug repurposing candidates for COVID-19	Hilson Andrade	02/04/2025	Mirabelli, Carmen and Wo	Proceedings of the	2021	Scopus		118	The globa	Article	10.1073/p	https://www.scopus.c		COVID-19	Animals; Antiviral Agents; Caco-2 Cells; C				Cited by: 107; All Open		Not related to leprosy
299	Wheat ergot fungus-derived and modified drug for inhibition of intracranial aneurysm rupture due to dysfunction of TLR-4 receptor in Alzhei	Hilson Andrade	02/04/2025	Debnath, Sandip and Sha	PLoS ONE	2023	Scopus		18	Backgrou	Article	10.1371/jo	https://www.scopus.com/inward/			Aged; Alzheimer Disease; Aneurysm, Rup				Cited by: 4; All Open Acc		Not related to leprosy
300	Global, regional, and national incidence and mortality burden of non-COVID-19 lower respiratory infections and aetiologies, 1990–2021: a s	Hilson Andrade	02/04/2025	Rose Grace Bender and S	The Lancet Infecti	2024	ence@Dir	74-1002	24	Summary		https://doi.	https://www.sciencedirect.com/science/article/pii/S147					1473-3099				Not related to leprosy

Id	title	data extractor	date of data extraction	author	journal	year	source	pages	volume	abstract	document_type	doi	url	affiliation	or_keywo	keywords	publisher	issn	nguag	note	lection_criter	Selection
301	Recent advances in point-of-care biosensors for the diagnosis of neglected tropical diseases	Hilson Andrade	02/04/2025	Patricia Batista Deroco an	Sensors and Actua	2021	ence@Dir	130821	349	Considering the progressive spr		https://doi.	https://www.sciencedirect.com/s			Point-of-care testing,		0925-4005				Duplicated
302	Novel compounds from endophytic fungi of Ceriops decandra inhibit breast cancer cell growth through estrogen receptor alpha in in-silico s	Hilson Andrade	02/04/2025	Mita Munshi and Md. Naz	Informatics in Med	2022	ence@Dir	101046	32	Endophytic fungi can thrive with		https://doi.	https://www.sciencedirect.com/s			Endophytic fungi, GC		2352-9148				Not related to AI
303	The Association Between Chronic Plaque Psoriasis and Nonalcoholic Fatty Liver Disease in Indian Patients: Results of a Pilot Study	Hilson Andrade	02/04/2025	Vikram K. Mahajan and N	Journal of Clinical	2022	ence@Dir	785-792	12	Background		https://doi.	https://www.sciencedirect.com/s			elastography, liver fib		0973-6883				Not related to leprosy
304	Quantitative and qualitative analysis of triterpenoid saponins in Achyranthes bidentata Blume via UHPLC-ELSD/UPLC-IM-Q-TOF-MS/MS a	Hilson Andrade	02/04/2025	Shivani Puri and Prateek	Microchemical Jou	2025	ence@Dir	112137	208	Achyranthes bidentata Blume is		https://doi.	https://www.sciencedirect.com/s			Blume, Ultra-high pe		0026-265X				Not related to AI
305	Are paid tools worth the cost? A prospective cross-over study to find the right tool for plagiarism detection	Hilson Andrade	02/04/2025	Abhishek Anil and Aswini	Heliyon	2023	ence@Dir	e19194	9	Background		https://doi.	https://www.sciencedirect.com/s			Plagiarism, iThenticat		2405-8440				Not related to leprosy
306	Targeting leucine-rich repeat serine/threonine-protein kinase 2 sensitizes pancreatic ductal adenocarcinoma to anti-PD-L1 immunotherapy	Hilson Andrade	02/04/2025	Kang Sun and Xiaozhen Z	Molecular Therapy	2023	ence@Dir	929-294	31	Pancreatic ductal adenocarcino		https://doi.	https://www.sciencedirect.com/s			LRRK2, PD-L1, PDA		1525-0016				Not related to leprosy
307	Structural basis and designing of peptide vaccine using PE-PGRS family protein of Mycobacterium ulcerans—An integrated vaccinomics a	Hilson Andrade	02/04/2025	Zulkar Nain and Mohamm	Molecular Immunc	2020	ence@Dir	146-163	120	Buruli ulcer is an emerging tissu		https://doi.	https://www.sciencedirect.com/s			Buruli ulcer, , PE-PG		0161-5890				Not related to leprosy
308	The Lancet Psychiatry Commission on youth mental health	Hilson Andrade	02/04/2025	Patrick D McGorry and Cr	The Lancet Psychi	2024	ence@Dir	731-774	11			https://doi.	https://www.sciencedirect.com/science/article/pii/S221					2215-0366				Not related to leprosy
309	Repurposing small-molecule drugs for modulating toxic protein aggregates in neurodegenerative diseases	Hilson Andrade	02/04/2025	Wei Liu and Gang Wang a	Drug Discovery To	2022	ence@Dir	994-200	27	Neurodegenerative diseases (N		https://doi.	https://www.sciencedirect.com/s			Drug repurposing, Sn		1359-6446				Not related to leprosy
310	Waste-to-value: Guidelines for the potential applications of Prosopis juliflora	Hilson Andrade	02/04/2025	Imane A. Saleh and Amina	Bioresource Techn	2023	ence@Dir	101678	24	This review describes the guidel		https://doi.	https://www.sciencedirect.com/s			, Invasion control, Wa		2589-014X				Not related to leprosy
311	Exploring the new horizons of drug repurposing: A vital tool for turning hard work into smart work	Hilson Andrade	02/04/2025	Rajesh Kumar and Seetha	European Journal	2019	ence@Dir	111602	182	Drug discovery and developmer		https://doi.	https://www.sciencedirect.com/s			Drug repurposing, Dr		0223-5234				Not related to AI
312	An alphavirus-derived self-amplifying mRNA encoding PpSP15-LmSTI1 fusion protein for the design of a vaccine against leishmaniasis	Hilson Andrade	02/04/2025	Nastaran Sadat Savar anc	Parasitology Interr	2022	ence@Dir	102577	89	The main aims of the present st		https://doi.	https://www.sciencedirect.com/s			Alphavirus, Self-ampl		1383-5769				Not related to leprosy
313	Research and analysis of regulatory framework and harmonisation of repurposing	Hilson Andrade	02/04/2025	Antonio Ivanov and Violet	PHARMACIA	2024	ence@Dir	1-7	71	Abstract		https://doi.	https://www.sciencedirect.com/s			drug repurposing, reg		0428-0296				Not related to leprosy
314	Global, regional, and national burden of disorders affecting the nervous system, 1990–2021: a systematic analysis for the Global Burden of	Hilson Andrade	02/04/2025	Jaimie D Steinmetz and K	The Lancet Neurol	2024	ence@Dir	344-381	23	Summary		https://doi.	https://www.sciencedirect.com/science/article/pii/S147					1474-4422				Surveys
315	Systematic identification and repurposing of FDA-approved drugs as antibacterial agents against Streptococcus pyogenes: In silico and in	Hilson Andrade	02/04/2025	Judith Gracia and Damodl	International Journ	2024	ence@Dir	128667	257	Streptococcus pyogenes (Group		https://doi.	https://www.sciencedirect.com/s			RpoC, RNA polymera		0141-8130				Not related to AI
316	Effects of a feedback intervention on antibiotic prescription control in primary care institutions based on a Health Information System: a clus	Hilson Andrade	02/04/2025	Junli Yang and Zhezhe Cu	Journal of Global A	2023	ence@Dir	51-60	33	ABSTRACT		https://doi.	https://www.sciencedirect.com/s			Antibiotics, Feedback		2213-7165				Not related to AI
317	Discovery of sulfone-resistant dihydropteroate synthase (DHPS) as a target enzyme for kaempferol, a natural flavanoid	Hilson Andrade	02/04/2025	Angamba Meetei Potshan	Heliyon	2020	ence@Dir	e03378	6	Kaempferol is a ubiquitous flavo		https://doi.	https://www.sciencedirect.com/s			Pharmaceutical scien		2405-8440				Not related to AI
318	Polypharmacology: The science of multi-targeting molecules	Hilson Andrade	02/04/2025	Abbas Kabir and Aaron M	Pharmacological F	2022	ence@Dir	106055	176	Polypharmacology is a concept		https://doi.	https://www.sciencedirect.com/s			Polypharmacology, M		1043-6618				Not related to AI
319	Ecopharmacognosy and the responsibilities of natural product research to sustainability	Hilson Andrade	02/04/2025	Geoffrey A. Cordell	Phytochemistry Le	2015	ence@Dir	332-346	11	The recently developed term “ec		https://doi.	https://www.sciencedirect.com/s			Ecopharmacognosy,		1874-3900				Not related to AI
320	Pathology Competencies in Medical Education and Educational Cases: Update 2023	Hilson Andrade	02/04/2025	Barbara E.C. Knollmann-F	Academic Patholo	2023	ence@Dir	100086	10	Pathology is a core component		https://doi.	https://www.sciencedirect.com/s			Pathology competenc		2374-2895				Not related to AI
321	Identification of LRRK2 Inhibitors through Computational Drug Repurposing	Hilson Andrade	02/04/2025	Shuoyan Tan and Ruiqian	ACS Chemical Ne	2023	ence@Dir	481-493	14	Parkinson’s disease (PD) is the		https://doi.	https://www.sciencedirect.com/s			leucine-rich repeat ki		1948-7193				Not related to leprosy
322	Multimodal AI/ML for discovering novel biomarkers and predicting disease using multi-omics profiles of patients with cardiovascular disease	Hilson Andrade	02/04/2025	DeGroat, William and Abd	Scientific Reports	2024	Scopus		14	Cardiovas	Article	10.1038/s	https://www.scopus.c		Artificial In	Artificial Intelligence; Biomarkers; Cardiova				Cited by: 1; All Open Acc		Not related to leprosy
323	Tsalli’s Entropy-based Segmentation Method for Accurate Pigmented Skin Lesion Identification	Hilson Andrade	02/04/2025	Bhakta, Ishita and Phadik	IETE Journal of Re	2022	Scopus	43 – 75	68	Skin disea	Article	10.1080/0	https://www.scopus.c		Computer-	Computer aided diagnosis; Dermatology;				Cited by: 2		Not related to AI
324	Prediction of Dynamic Plantar Pressure From Insole Intervention for Diabetic Patients Based on Patch-Based Multilayer Perceptron With L	Hilson Andrade	02/04/2025	Zhang, Li-Ying and Ma, Ze	IEEE Access	2024	Scopus	55 – 10	12	Assessing	Article	10.1109/A	https://www.scopus.c		Diabetes;	Medical imaging; Multilayer neural network				Cited by: 0; All Open Acc		Not related to AI
325	Physician preference for receiving machine learning predictive results: A cross-sectional multicentric study	Hilson Andrade	02/04/2025	Wichmann, Roberta More	PLoS ONE	2022	Scopus		17	Artificial i	Article	10.1371/jo	https://www.scopus.com/inward/			Artificial Intelligence; COVID-19; Cross-Se				Cited by: 1; All Open Acc		Not related to leprosy
326	Discovering interesting relationships in the factors relating to the elderly living alone in Thailand using association analysis	Hilson Andrade	02/04/2025	Kittiphattanabawon, Nichr	International Journ	2023	Scopus	68 – 18	15	Many cou	Article	10.1504/iji	https://www.scopus.c		aloneness;	association analysis; association rules; el				Cited by: 0		Not related to AI
327	Timely and effective media coverage's role in the spread of Corona Virus Disease 2019	Hilson Andrade	02/04/2025	Wang, Yan and Qing, Fen	Mathematical Meth	2024	Scopus	90 – 35	47	For all hu	Conference paper	10.1002/m	https://www.scopus.c		Corona Vi	Deep learning; Disease control; Sensitivity				Cited by: 1; All Open Acc		Not related to leprosy
328	Next-generation drug repurposing using human genetics and network biology	Hilson Andrade	02/04/2025	Serguei Nabirotchkin and	Current Opinion in	2020	ence@Dir	78-92	51	Drug repurposing has attracted		https://doi.	https://www.sciencedirect.com/science/article/pii/S147					1471-4892				Not related to AI
329	Blood RNA signature RISK4LEP predicts leprosy years before clinical onset	Hilson Andrade	02/04/2025	Maria Tió-Coma and Szyn	EBioMedicine	2021	ence@Dir	103379	68	Background		https://doi.	https://www.sciencedirect.com/s			Leprosy, Diagnostics,		2352-3964				Included
330	Using Big Data to Discover Diagnostics and Therapeutics for Gastrointestinal and Liver Diseases	Hilson Andrade	02/04/2025	Benjamin Wooden and Ni	Gastroenterology	2017	ence@Dir	53-67.e3	152	Technologies such as genome s		https://doi.	https://www.sciencedirect.com/s			Big Data, Translation		0016-5085				Not related to leprosy
331	Underserved populations and health equity in dermatology: Digital medicine and the role of artificial intelligence	Hilson Andrade	02/04/2025	Eran C. Gwillim and Soray	Clinics in Dermato	2024	ence@Dir	498-506	42	We have reviewed the current lit		https://doi.	https://www.sciencedirect.com/science/article/pii/S073					0738-081X		Artificial Intelligence II		Surveys
332	Contribution of machine learning approaches in response to SARS-CoV-2 infection	Hilson Andrade	02/04/2025	Mohammad Sadeq Mottac	Informatics in Med	2021	ence@Dir	100526	23	Problem		https://doi.	https://www.sciencedirect.com/s			SARS-CoV-2, COVID		2352-9148				Not related to leprosy
333	Reimagining leprosy elimination with AI analysis of a combination of skin lesion images with demographic and clinical data	Hilson Andrade	02/04/2025	Raquel R Barbieri and Yix	The Lancet Regior	2022	ence@Dir	100192	9	Summary		https://doi.	https://www.sciencedirect.com/s			Leprosy, Artificial inte		2667-193X				Included
334	ChatGPT-Generated Differential Diagnosis Lists for Complex Case–Derived Clinical Vignettes: Diagnostic Accuracy Evaluation	Hilson Andrade	02/04/2025	Takanobu Hirosawa and F	JMIR Medical Infor	2023	ence@Direct		11	Background		https://doi.	https://www.sciencedirect.com/s			artificial intelligence,		2291-9694				Not related to leprosy
335	Artificial intelligence approach fighting COVID-19 with repurposing drugs	Hilson Andrade	02/04/2025	Yi-Yu Ke and Tzu-Ting Pe	Biomedical Journa	2020	ence@Dir	355-362	43	Background		https://doi.	https://www.sciencedirect.com/s			AI, DNN, COVID-19,		2319-4170				Not related to leprosy
336	Dermatologic Fungal Neglected Tropical Diseases—Part II. Management and Morbidity	Hilson Andrade	02/04/2025	Kaya L. Curtis and Jeremy	Journal of the Ame	2024	ence@Direct			In this part 2 of a 2-part continui		https://doi.	https://www.sciencedirect.com/s			neglected tropical dis		0190-9622				Not related to leprosy
337	The COVID-19 Drug and Gene Set Library	Hilson Andrade	02/04/2025	Maxim V. Kuleshov and D	Patterns	2020	ence@Dir	100090	1	Summary		https://doi.	https://www.sciencedirect.com/s			DSML 3: Developmer		2666-3899				Not related to leprosy
338	Integrating bacterial molecular genetics with chemical biology for renewed antibacterial drug discovery	Hilson Andrade	02/04/2025	Susannah L. Parkhill and	Biochemical Journ	2024	ence@Dir	839-864	481	The application of dyes to under		https://doi.	https://www.sciencedirect.com/s			antimicrobial resistan		1470-8728				Not related to AI
339	Patient Education for Preventative Health Care	Hilson Andrade	02/04/2025	Sarah Schuur and Brittany	Physician Assistar	2024	ence@Dir	553-566	9			https://doi.	https://www.sciencedirect.com/s			Preventative medicin		2405-7991		Advances in Patient Edu		Not related to AI
340	Laying in silico pipelines for drug repositioning: a paradigm in ensemble analysis for neurodegenerative diseases	Hilson Andrade	02/04/2025	Nikolas Dovrolis and Geo	Drug Discovery To	2017	ence@Dir	805-813	22	When faced with time- and mon		https://doi.	https://www.sciencedirect.com/science/article/pii/S135					1359-6446				Not related to leprosy
341	SQEIR: An epidemic virus spread analysis and prediction model	Hilson Andrade	02/04/2025	Yichun Wu and Yaqi Sun a	Computers and El	2022	ence@Dir	108230	102	In 2019, a new strain of coronav		https://doi.	https://www.sciencedirect.com/s			Susceptible quarantir		0045-7906				Not related to leprosy
342	Analysis of heartbeat time series via machine learning for detection of illnesses	Hilson Andrade	02/04/2025	Sidney T. {da Silva} and M	Chaos, Solitons &	2023	ence@Dir	113388	171	The heart, a component of the c		https://doi.	https://www.sciencedirect.com/s			Machine learning, He		0960-0779				Not related to leprosy
343	Evaluating the World Health Organization’s SkinNTDs App as a Training Tool for Skin Neglected Tropical Diseases in Ghana and Kenya: C	Hilson Andrade	02/04/2025	Mireia Cano and José A R	Journal of Medical	2024	ence@Direct		26	Backgro		https://doi.	https://www.sciencedirect.com/s			SkinNTDs app, mHea		1438-8871				Excluded by QA3
344	Relevance-diversity algorithm for feature selection and modified Bayes for prediction	Hilson Andrade	02/04/2025	M. Shaheen and N. Nahe	Alexandria Engine	2023	ence@Dir	329-342	66	Big data analytics uncovers hid		https://doi.	https://www.sciencedirect.com/s			Naive Bayes, Feature		1110-0168				Not related to leprosy
345	Unb-DPC: Identify mycobacterial membrane protein types by incorporating un-biased dipeptide composition into Chou's general PseAAC	Hilson Andrade	02/04/2025	Muslim Khan and Maqsoo	Journal of Theoret	2017	ence@Dir	13-19	415	This study investigates an efficie		https://doi.	https://www.sciencedirect.com/s			Mycobacterium, Over		0022-5193				Included
346	Holistic Integrative Pharmacy and disease control	Hilson Andrade	02/04/2025	Jiao GUO and Gang CHE	Journal of Holistic	2020	ence@Dir	79-90	1	[Abstract]		https://doi.	https://www.sciencedirect.com/s			Holistic integrative ph		2707-3688				Not related to AI
347	Review on the evolving role of infrared thermography in oncological applications	Hilson Andrade	02/04/2025	Anwit Pokharel and Nisha	Infrared Physics &	2024	ence@Dir	105399	140	In recent years, researchers hav		https://doi.	https://www.sciencedirect.com/s			Infrared thermograph		1350-4495				Surveys
348	HARP: a database of structural impacts of systematic missense mutations in drug targets of Mycobacterium leprae	Hilson Andrade	02/04/2025	Sundeep Chaitanya Veditl	Computational and	2020	ence@Dir	692-370	18	Computational Saturation Mutag		https://doi.	https://www.sciencedirect.com/s			Drug resistance, Muta		2001-0370				Not related to AI
349	TN strain proteome mediated therapeutic target mapping and multi-epitopic peptide-based vaccine development for Mycobacterium leprae	Hilson Andrade	02/04/2025	Manojit Bhattacharya and	Infection, Genetics	2022	ence@Dir	105245	99	Leprosy is a significant universa		https://doi.	https://www.sciencedirect.com/s			Leprosy, Therapeutic		1567-1348				Not related to AI
350	Ethical Implications of the Use of Language Analysis Technologies for the Diagnosis and Prediction of Psychiatric Disorders	Hilson Andrade	02/04/2025	Alexandre Andrade Loch a	JMIR Mental Healt	2022	ence@Direct		9	Recent developments in artificia		https://doi.	https://www.sciencedirect.com/s			at-risk mental state, p		2368-7959				Not related to leprosy
351	FIAMol-AB: A feature fusion and attention-based deep learning method for enhanced antibiotic discovery	Hilson Andrade	02/04/2025	Shida He and Xiucai Ye a	Computers in Biolo	2024	ence@Dir	107762	168	Antibiotic resistance continues t		https://doi.	https://www.sciencedirect.com/s			Computational biolog		0010-4825				Not related to leprosy
352	Adaptive ranking of search results by considering user's comprehension	Hilson Andrade	02/04/2025	Nakatani, Makoto and Jatowt, Adam and Tan		2010	l Digital Library			Given a search query, conventic		10.1145/2	https://doi.org/10.1145/2108616.			wikipedia	Association for Computing Machinery					Not related to leprosy
353	The perception of system and the reduction of uncertainty	Hilson Andrade	02/04/2025	Flagle, C. D.		1987	l Digital Lit	137–142		Until the mid 1960's we had two		10.1145/4	https://doi.org/10.1145/41526.41539				Association for Computing Machinery					Not related to AI
354	Skin disorder diagnosis assisted by lesion color adaptation	Hilson Andrade	02/04/2025	Petrellis, Nikos		2018	l Digital Lit	208–212		A technique that counterbalance		10.1145/3	https://doi.org/10.1145/3291533.			smart pho	Association for Computing Machinery					Not related to leprosy
355	Using Energy-Minimization Profiles to Measure Protein Resistance to Drugs	Hilson Andrade	02/04/2025	Thompson, Edward and Thackray, Tess and		2020	l Digital Library			A mutation to the amino acid se		10.1145/3	https://doi.org/10.1145/3388440.			rigidity ana	Association for Computing Machinery					Not related to leprosy
356	Characterization of Local Attitudes Toward Immigration Using Social Media	Hilson Andrade	02/04/2025	Freire-Vidal, Yerka and Graells-Garrido, Edua		2019	l Digital Lit	783–790		Migration is a worldwide phenor		10.1145/3	https://doi.org/10.1145/3308560.			Twitter, Se	Association for Computing Machinery					Not related to leprosy
357	Weekends as social distancing and their effect on the spread of influenza	Hilson Andrade	02/04/2025	Philip C. CooleySarah M. BartschShawn T. B		2016	pringer Link					10.1007/s	http://link.springer.com/article/10.1007/s105				Computational and Mathematical Organization Theory					Not related to leprosy
358	Hair detection and lesion segmentation in dermoscopic images using domain knowledge	Hilson Andrade	02/04/2025	Sameena PathanK. Gopalakrishna PrabhuP.		2018	pringer Link					10.1007/s	http://link.springer.com/article/10.1007/s115				Medical & Biological Engineering & Computing					Not related to leprosy
359	Scaling up of parenting support to prevent violence against children in Tanzania: insights from policymakers and service providers	Hilson Andrade	02/04/2025	Sabine van Tuyll van Serooskereken Rakoto		2025	pringer Link					10.1186/s	http://link.springer.com/article/10.1186/s430				Implementation Science Communications					Not related to leprosy
360	Use of Adjectives in Abstracts when Reporting Results of Randomized, Controlled Trials from Industry and Academia	Hilson Andrade	02/04/2025	M. Soledad CepedaJesse A. BerlinSusan C.		2015	pringer Link					10.1007/s	http://link.springer.com/article/10.1007/s402				Drugs in R&D					Not related to leprosy
361	Engineering and design of promising T-cell-based multi-epitope vaccine candidates against leishmaniasis	Hilson Andrade	02/04/2025	Esmaeil Roohparvar BasmenjMahshid Arasto		2023	pringer Link					10.1038/s	http://link.springer.com/article/10.1038/s415				Scientific Reports					Not related to leprosy
362	Association of DNA repair and cell cycle gene variations with breast cancer risk in Northeast Indian population: a multiple interaction analys	Hilson Andrade	02/04/2025	Mishi Kaushal WassonPradeep Singh Chauh		2014	pringer Link					10.1007/s	http://link.springer.com/article/10.1007/s132				Tumor Biology					Not related to leprosy
363	Unveiling the anti-inflammatory potential of Acalypha indica L. and analyzing its research trend: digging deep to learn deep	Hilson Andrade	02/04/2025	Divya SharmaSupriya SharmaVivekananda M		2024	pringer Link					10.1007/s	http://link.springer.com/article/10.1007/s002				Naunyn-Schmiedeberg's Archives of Pharmacology					Not related to leprosy
364	The immunological and genetic aspects in psoriasis	Hilson Andrade	02/04/2025	Liangdan SunXuejun Zhang		2014	pringer Link					10.1186/s	http://link.springer.com/article/10.1186/s405				Applied Informatics					Not related to leprosy
365	On the Formation of Regular Patterns from Drying Droplets and Their Potential Use for Bio-Medical Applications	Hilson Andrade	02/04/2025	Khellil Sefiane		2010	pringer Link					10.1016/S	http://link.springer.com/article/10.1016/S16				Journal of Bionic Engineering					Not related to AI
366	Revisiting the ethnomedicinal, ethnopharmacological, phytoconstituents and phytoremediation of the plant Solanum viarum Dunal	Hilson Andrade	02/04/2025	Mehak ThakurRachna VermaDinesh KumarF		2024	pringer Link					10.1007/s	http://link.springer.com/article/10.1007/s002				Naunyn-Schmiedeberg's Archives of Pharmacology					Not related to leprosy
367	Impact of work experiences on perceived organizational support: a study among healthcare professionals	Hilson Andrade	02/04/2025	G. N. SumathiT. J. KamalanabhanM. Thenmo		2015	pringer Link					10.1007/s	http://link.springer.com/article/10.1007/s001				AI & SOCIETY					Not related to leprosy
368	A study of the velocity field during evaporation of sessile water and water/ethanol drops	Hilson Andrade	02/04/2025	John R. E. ChristyKhellil SefianeEuan Munro		2010	pringer Link					10.1016/S	http://link.springer.com/article/10.1016/S16				Journal of Bionic Engineering					Not related to leprosy
369	Biofilm-mediated infections by multidrug-resistant microbes: a comprehensive exploration and forward perspectives	Hilson Andrade	02/04/2025	Mai M. ZaferGamal A. MohamedSabrin R. M.		2024	pringer Link					10.1007/s	http://link.springer.com/article/10.1007/s002				Archives of Microbiology					Not related to AI
370	Discovery of novel DprE1 inhibitors via computational bioactivity fingerprints and structure-based virtual screening	Hilson Andrade	02/04/2025	Xue-ping HuLiu YangXin ChaiYi-xuan LeiMd		2022	pringer Link					10.1038/s	http://link.springer.com/article/10.1038/s414				Acta Pharmacologica Sinica					Not related to leprosy
371	Development, detection and decipherment of obfuscated fingerprints in humans: Implications for forensic casework	Hilson Andrade	02/04/2025	Tej KaurNandini ChitaraAnkita GuleriaRakes		2023	pringer Link					10.1007/s	http://link.springer.com/article/10.1007/s001				The Science of Nature					Not related to leprosy
372	An Integrated Mathematical Model of Thrombin-, Histamine-and VEGF-Mediated Signalling in Endothelial Permeability	Hilson Andrade	02/04/2025	XN WeiBC HanJX ZhangXH LiuCY TanYY Ji		2011	pringer Link					10.1186/1	http://link.springer.com/article/10.1186/1752				BMC Systems Biology					Not related to AI
373	Body temperature estimation of a moving subject from thermographic images	Hilson Andrade	02/04/2025	Guillaume-Alexandre BilodeauAtousa Torabi		2012	pringer Link					10.1007/s	http://link.springer.com/article/10.1007/s001				Machine Vision and Applications					Not related to leprosy
374	Antibiotic prescribing patterns at children’s outpatient departments of primary care institutions in Southwest China	Hilson Andrade	02/04/2025	Wenju WangShitao YuXunrong ZhouLei Wan		2022	pringer Link					10.1186/s	http://link.springer.com/article/10.1186/s128				BMC Primary Care					Not related to AI
375	Pathogen stress heightens sensorimotor dimensions in the human collective semantic space	Hilson Andrade	02/04/2025	Ze FuHuimin ChenZhan LiuMaosong SunZhi		2025	pringer Link					10.1038/s	http://link.springer.com/article/10.1038/s442				Communications Psychology					Not related to leprosy
376	Infectious keratitis: an update on epidemiology, causative microorganisms, risk factors, and antimicrobial resistance	Hilson Andrade	02/04/2025	Darren Shu Jeng TingCharlotte Shan HoRas		2021	pringer Link					10.1038/s	http://link.springer.com/article/10.1038/s414				Eye					Not related to AI
377	Specificity ratings for English data	Hilson Andrade	02/04/2025	Andrea Amelio RavelliMarianna Marcella Bol		2024	pringer Link					10.1007/s	http://link.springer.com/article/10.1007/s103				Cognitive Processing					Not related to leprosy
378	Labial opacity and roundness harmony in Nawuri	Hilson Andrade	02/04/2025	Roderic F. Casali		1995	pringer Link					10.1007/B	http://link.springer.com/article/10.1007/BF0				Natural Language & Linguistic Theory					Not related to leprosy
379	One Health tropical wetlands: a transdisciplinary framework for assessing the risks of emerging zoonotic diseases in the Brazilian Pantanal	Hilson Andrade	02/04/2025	Fabio de Oliveira RoqueHeitor Miraglia Herre		2025	pringer Link					10.1007/s	http://link.springer.com/article/10.1007/s112				Wetlands Ecology and Management					Not related to leprosy
380	The role of the adaptive immune system and T cell dysfunction in neurodegenerative diseases	Hilson Andrade	02/04/2025	Alexa DeMaioShikhar MehrotraKumar Samba		2022	pringer Link					10.1186/s	http://link.springer.com/article/10.1186/s129				Journal of Neuroinflammation					Not related to AI
381	Proceedings of the 26th European Paediatric Rheumatology Congress: part 1	Hilson Andrade	02/04/2025	unknown		2020	pringer Link					10.1186/s	http://link.springer.com/article/10.1186/s129				Pediatric Rheumatology					Not related to AI
382	Tuberculosis screening costs and cost-effectiveness in high-risk groups: a systematic review	Hilson Andrade	02/04/2025	H. AlsdurfB. EmpringhamC. MillerA. Zwerling		2021	pringer Link					10.1186/s	http://link.springer.com/article/10.1186/s128				BMC Infectious Diseases					Surveys
383	The inflammasome-activating poxvirus peptide IAMP29 promotes antimicrobial and anticancer responses	Hilson Andrade	02/04/2025	Taylor RohWonhyoung SeoMinho WonWoo S		2024	pringer Link					10.1038/s	http://link.springer.com/article/10.1038/s122				Experimental & Molecular Medicine					Not related to AI
384	From HIV to Mpox: Socio-discursive Analysis of Communicable Diseases, Stigma, and Pathologization in Sexual Minorities in Brazil	Hilson Andrade	02/04/2025	João Cruz NetoAmanda Cavalcante MaiaVio		2024	pringer Link					10.1007/s	http://link.springer.com/article/10.1007/s105				Archives of Sexual Behavior					Not related to leprosy
385	Clinical Presentation and Diagnosis of Drug Reaction with Eosinophilia and Systemic Symptoms (DReSS) in Children: A Scoping Review	Hilson Andrade	02/04/2025	Frances St George-HyslopNicole Cherepach		2024	pringer Link					10.1007/s	http://link.springer.com/article/10.1007/s120				Clinical Reviews in Allergy & Immunology					Surveys
386	Raman spectroscopy and mass spectrometry identifies a unique group of epidermal lipids in active discoid lupus erythematosus	Hilson Andrade	02/04/2025	Hannah U HoltkampClaude AguergarayKalita		2023	pringer Link					10.1038/s	http://link.springer.com/article/10.1038/s415				Scientific Reports					Duplicated
387	The glue degraders	Hilson Andrade	02/04/2025	Ken Garber		2024	pringer Link					10.1038/s	http://link.springer.com/article/10.1038/s415				Nature Biotechnology					Not related to leprosy
388	Getting the goods: an analysis of implications of the ‘education as delivery’ metaphor for the AI age	Hilson Andrade	02/04/2025	Lucinda McKnightAndy Morgan		2024	pringer Link					10.1007/s	http://link.springer.com/article/10.1007/s133				The Australian Educational Researcher					Not related to leprosy
389	Cycling back to folate metabolism in cancer	Hilson Andrade	02/04/2025	Younghwan LeeKaren H. VousdenMarc Henr		2024	pringer Link					10.1038/s	http://link.springer.com/article/10.1038/s430				Nature Cancer					Not related to leprosy
390	Mastering health: liberating beauty	Hilson Andrade	02/04/2025	Bernard PerbalSabine Gabaron		2021	pringer Link					10.1007/s	http://link.springer.com/article/10.1007/s120				Journal of Cell Communication and Signaling					Not related to AI
391	Empowering health: exploring the vital role of facilitator organisations in supporting chronic disease patients in Assam, India	Hilson Andrade	02/04/2025	Hiranmoyee BhuyanSeema S. Singha		2024	pringer Link					10.1007/s	http://link.springer.com/article/10.1007/s439				Research in Health Services & Regions					Not related to AI
392	Investigating the spatiotemporal characteristics and medical response during the initial COVID-19 epidemic in six Chinese cities	Hilson Andrade	02/04/2025	Li LanGang LiMuhammad Sajid MehmoodTir		2024	pringer Link					10.1038/s	http://link.springer.com/article/10.1038/s415				Scientific Reports					Not related to leprosy
393	A Clinical Decision Support System with an Integrated EMR for Diagnosis of Peripheral Neuropathy	Hilson Andrade	02/04/2025	Reeda KunhimangalamSujith OvallathPaul K		2014	pringer Link					10.1007/s	http://link.springer.com/article/10.1007/s109				Journal of Medical Systems					Not related to AI
394	COVID-19: Antiviral Agents, Antibody Development and Traditional Chinese Medicine	Hilson Andrade	02/04/2025	Wenyi GuanWendong LanJing ZhangShan Z		2020	pringer Link					10.1007/s	http://link.springer.com/article/10.1007/s122				Virologica Sinica					Not related to leprosy
395	Outcomes of the EMDataResource cryo-EM Ligand Modeling Challenge	Hilson Andrade	02/04/2025	Catherine L. LawsonAndriy KryshtafovychGri		2024	pringer Link					10.1038/s	http://link.springer.com/article/10.1038/s415				Nature Methods					Not related to leprosy
396	Synthetic biology and governance research in China: a 40-year evolution	Hilson Andrade	02/04/2025	Li TangJennifer KuzmaXi ZhangXinyu SongY		2023	pringer Link					10.1007/s	http://link.springer.com/article/10.1007/s111				Scientometrics					Not related to leprosy
397	Duckweeds: their utilization, metabolites and cultivation	Hilson Andrade	02/04/2025	GahYoung BaekMaham SaeedHyung-Kyoon		2021	pringer Link					10.1186/s	http://link.springer.com/article/10.1186/s137				Applied Biological Chemistry					Not related to leprosy
398	COVID-19 drug discovery and treatment options	Hilson Andrade	02/04/2025	Jasper Fuk-Woo ChanShuofeng YuanHin Ch		2024	pringer Link					10.1038/s	http://link.springer.com/article/10.1038/s415				Nature Reviews Microbiology					Not related to leprosy
399	Fungal coexistence in the skin mycobiome: a study involving Malassezia, Candida, and Rhodotorula	Hilson Andrade	02/04/2025	Bharati NaikJayaprakash SasikumarVishal B		2024	pringer Link					10.1186/s	http://link.springer.com/article/10.1186/s135				AMB Express					Not related to leprosy

Id	title	data extractor	date of data extraction	author	journal	year	source	pages	volume	abstract	document_type	doi	url	affiliation	ıor_keywc	keywords	publisher	issn	ınguag	note	lection_criter	Selection
400	Abstracts from the 5th International Conference on Prevention & Infection Control (ICPIC 2019)	Hilson Andrade	02/04/2025	unknown		2019	pringer Link					10.1186/s	http://link.springer.com/article/10.1186/s137				Antimicrobial Resistance & Infection Control					Not related to AI
401	Morphological and clinical study of lateral malleolus fractures based on fracture mapping: a retrospective study	Hilson Andrade	02/04/2025	Ruizhi CaiShuo FengChuhan ChenHao LuHa		2024	pringer Link					10.1186/s	http://link.springer.com/article/10.1186/s130				Journal of Orthopaedic Surgery and Research					Not related to leprosy
402	Biomechanics of Musculoskeletal System and Its Biomimetic Implications: A Review	Hilson Andrade	02/04/2025	Lei RenZhihui QianLuquan Ren		2014	pringer Link					10.1016/S	http://link.springer.com/article/10.1016/S167				Journal of Bionic Engineering					Surveys
403	Mycobacterium tuberculosis as teacher	Hilson Andrade	02/04/2025	Carl Nathan		2023	pringer Link					10.1038/s	http://link.springer.com/article/10.1038/s415				Nature Microbiology					Not related to leprosy
404	Evidence-based universal health coverage interventions delivery in infectious disease of poverty elimination and eradication	Hilson Andrade	02/04/2025	Ernest TamboChidiebere E. UgwuAmberbir A		2024	pringer Link					10.1186/s	http://link.springer.com/article/10.1186/s402				Infectious Diseases of Poverty					Not related to AI
405	Transforming clinical microbiology with bacterial genome sequencing	Hilson Andrade	02/04/2025	Xavier DidelotRory BowdenDaniel J. WilsonT		2012	pringer Link					10.1038/n	http://link.springer.com/article/10.1038/nrg3				Nature Reviews Genetics					Not related to AI
406	Ecological Threats and Cultural Systems	Hilson Andrade	02/04/2025	Soheil ShapouriYasaman Rafiee		2024	pringer Link					10.1007/s	http://link.springer.com/article/10.1007/s121				Human Nature					Not related to leprosy
407	Construction and effectiveness evaluation of a knowledge-based infectious disease monitoring and decision support system	Hilson Andrade	02/04/2025	Mengying WangMo JiaZhenhao WeiWei War		2023	pringer Link					10.1038/s	http://link.springer.com/article/10.1038/s415				Scientific Reports					Not related to AI
408	Associative patterns in health data: exploring new techniques	Hilson Andrade	02/04/2025	Diego RodriguesGuilherme RibeiroVilson Siq		2022	pringer Link					10.1007/s	http://link.springer.com/article/10.1007/s125				Health and Technology					Not related to AI
409	ECR 2016 Book of Abstracts - E - Authors' Index	Hilson Andrade	02/04/2025	unknown		2016	pringer Link					10.1007/s	http://link.springer.com/article/10.1007/s132				Insights into Imaging					Not related to leprosy
410	Network pharmacology, molecular docking, and molecular dynamics simulation to elucidate the mechanism of anti-aging action of Tinospor	Hilson Andrade	02/04/2025	Amisha BishtDisha TewariSanjay KumarSubł		2024	pringer Link					10.1007/s	http://link.springer.com/article/10.1007/s110				Molecular Diversity					Not related to leprosy
411	Integrated bioinformatics analysis reveals marker genes and immune infiltration for pulmonary arterial hypertension	Hilson Andrade	02/04/2025	Shengxin TangYue LiuBin Liu		2022	pringer Link					10.1038/s	http://link.springer.com/article/10.1038/s415				Scientific Reports					Not related to leprosy
412	Potential anti-TB investigational compounds and drugs with repurposing potential in TB therapy: a conspectus	Hilson Andrade	02/04/2025	Adetomiwa A. AdenijiKirsten E. KnollDu Toit L		2020	pringer Link					10.1007/s	http://link.springer.com/article/10.1007/s002				Applied Microbiology and Biotechnology					Not related to leprosy
413	Nearest neighbour classification of Indian sign language gestures using kinect camera	Hilson Andrade	02/04/2025	ZAFAR AHMED ANSARIGAURAV HARIT		2016	pringer Link					10.1007/s	http://link.springer.com/article/10.1007/s120				Sadhana					Not related to leprosy
414	MycoBASE: expanding the functional annotation coverage of mycobacterial genomes	Hilson Andrade	02/04/2025	Benjamin J. GarciaGargi DattaRebecca M. D		2015	pringer Link					10.1186/s	http://link.springer.com/article/10.1186/s128				BMC Genomics					Duplicated
415	Exploring Medicinal Plant Legacy for Drug Discovery in Post-genomic Era	Hilson Andrade	02/04/2025	Satendra SinghDev Bukhsh SinghShivani Sir		2019	pringer Link					10.1007/s	http://link.springer.com/article/10.1007/s400				Proceedings of the National Academy of Sciences, India					Not related to leprosy
416	Nature Biotechnology's academic spinouts of 2016	Hilson Andrade	02/04/2025	Aaron BouchieLaura DeFrancescoCormac S		2017	pringer Link					10.1038/n	http://link.springer.com/article/10.1038/nbt.3				Nature Biotechnology					Not related to AI
417	Positive selection in admixed populations from Ethiopia	Hilson Andrade	02/04/2025	Sandra WalshLuca PaganiYali XueHafid Laa		2020	pringer Link					10.1186/s	http://link.springer.com/article/10.1186/s128				BMC Genetics					Not related to leprosy
418	"World in motion" – emulsion adjuvants rising to meet the pandemic challenges	Hilson Andrade	02/04/2025	Derek T. O'HaganRobbert van der MostRush		2021	pringer Link					10.1038/s	http://link.springer.com/article/10.1038/s415				npj Vaccines					Not related to leprosy
419	Quantification and clustering of phenotypic screening data using time-series analysis for chemotherapy of schistosomiasis	Hilson Andrade	02/04/2025	Hyokyeong LeeAsher Moody-DavisUtsab Sa		2012	pringer Link					10.1186/1	http://link.springer.com/article/10.1186/1471				BMC Genomics					Not related to leprosy
420	A novel model for prediction of stability constants of the thiosemicarbazone ligands with different types of toxic heavy metal ions using struc	Hilson Andrade	02/04/2025	Mohammad Hossein KeshavarzZeinab Shira		2022	pringer Link					10.1007/s	http://link.springer.com/article/10.1007/s113				Environmental Science and Pollution Research					Not related to leprosy
421	The experience of albinism in France: a qualitative study on dyads of parents and their adult child with albinism	Hilson Andrade	02/04/2025	Hugo FournierMarie HasdenteufelConstance		2024	pringer Link					10.1186/s	http://link.springer.com/article/10.1186/s129				BMC Medicine					Not related to leprosy
422	MicroRNA Guided In Silico Drug Repositioning for Malaria	Hilson Andrade	02/04/2025	Sowmya R. PrabhuAkshay Pramod WareKap		2024	pringer Link					10.1007/s	http://link.springer.com/article/10.1007/s116				Acta Parasitologica					Not related to leprosy
423	Positive rate of wheat allergens in the Chinese allergic population: a systematic review and meta-analysis	Hilson Andrade	02/04/2025	Fengmei YangXinyi ZhaoWenfeng LiuBo Zho		2023	pringer Link					10.1038/s	http://link.springer.com/article/10.1038/s415				Scientific Reports					Surveys
424	The Hmong Medical Corpus: a biomedical corpus for a minority language	Hilson Andrade	02/04/2025	Nathan M. White		2022	pringer Link					10.1007/s	http://link.springer.com/article/10.1007/s105				Language Resources and Evaluation					Not related to leprosy
425	Identifying corridors for landscape connectivity using species distribution modeling of Hydnocarpus kurzii (King) Warb., a threatened specie	Hilson Andrade	02/04/2025	Koushik MajumdarDibyendu AdhikariBadal K		2019	pringer Link					10.1007/s	http://link.springer.com/article/10.1007/s113				Landscape and Ecological Engineering					Not related to leprosy
426	XXIV World Allergy Congress 2015	Hilson Andrade	02/04/2025	Heung-Man LeeIl-Ho ParkJae-Min ShinHyun		2016	pringer Link					10.1186/s	http://link.springer.com/article/10.1186/s404				World Allergy Organization Journal					Not related to leprosy
427	Novel genome polymorphisms in BCG vaccine strains and impact on efficacy	Hilson Andrade	02/04/2025	Andrea S LeungVanessa TranZuowei WuXup		2008	pringer Link					10.1186/1	http://link.springer.com/article/10.1186/1471				BMC Genomics					Not related to AI
428	A data model and algebra for probabilistic complex values	Hilson Andrade	02/04/2025	Thomas EiterThomas LukasiewiczMichael W		2001	pringer Link					10.1023/A	http://link.springer.com/article/10.1023/A%3				Annals of Mathematics and Artificial Intelligence					Not related to leprosy
429	DrugRep: an automatic virtual screening server for drug repurposing	Hilson Andrade	02/04/2025	Jian-hong GanJi-xiang LiuYang LiuShu-wen		2023	pringer Link					10.1038/s	http://link.springer.com/article/10.1038/s414				Acta Pharmacologica Sinica					Not related to leprosy
430	Abstracts Accepted for American Conference on Pharmacometrics 2013	Hilson Andrade	02/04/2025	unknown		2013	pringer Link					10.1007/s	http://link.springer.com/article/10.1007/s109				Journal of Pharmacokinetics and Pharmacodynamics					Not related to leprosy
431	Eudemonia of a machine	Hilson Andrade	02/04/2025	Mois Navon		2024	pringer Link					10.1007/s	http://link.springer.com/article/10.1007/s436				AI and Ethics					Not related to leprosy
432	Cancer metastasis chemoprevention prevents circulating tumour cells from germination	Hilson Andrade	02/04/2025	Xiaodong XieYumei LiShu LianYusheng LuLe		2022	pringer Link					10.1038/s	http://link.springer.com/article/10.1038/s413				Signal Transduction and Targeted Therapy					Not related to leprosy
433	Sondra Fraleigh: Butoh: Metamorphic Dance and Global Alchemy	Hilson Andrade	02/04/2025	Corinna Brown		2012	pringer Link					10.1007/s	http://link.springer.com/article/10.1007/s104				American Journal of Dance Therapy					Not related to leprosy
434	drones, swarms and becoming-insect: feminist utopias and posthuman politics	Hilson Andrade	02/04/2025	Lauren Wilcox		2017	pringer Link					10.1057/s	http://link.springer.com/article/10.1057/s413				Feminist Review					Not related to leprosy
435	COVID-19, Personal Data Protection and Privacy in India	Hilson Andrade	02/04/2025	Mohamad Ayub DarShahnawaz Ahmad Wani		2023	pringer Link					10.1007/s	http://link.springer.com/article/10.1007/s416				Asian Bioethics Review					Not related to leprosy
436	Foreword	Hilson Andrade	02/04/2025	V V S SarmaB Yegnanarayana		1994	pringer Link					10.1007/B	http://link.springer.com/article/10.1007/BF0				Sadhana					Not related to leprosy
437	Society of General Internal Medicine	Hilson Andrade	02/04/2025	unknown		2011	pringer Link					10.1007/s	http://link.springer.com/article/10.1007/s116				Journal of General Internal Medicine					Not related to leprosy
438	Modified adaptive neuro-fuzzy inference system (M-ANFIS) based multi-disease analysis of healthcare Big Data	Hilson Andrade	02/04/2025	K. VidhyaR. Shanmugalakshmi		2020	pringer Link					10.1007/s	http://link.springer.com/article/10.1007/s112				The Journal of Supercomputing					Not related to leprosy
439	A Study on Transitions to Knowledge-Based Service in China's Publishing Industry	Hilson Andrade	02/04/2025	Li ZhangSuping WuDan Zhou		2020	pringer Link					10.1007/s	http://link.springer.com/article/10.1007/s121				Publishing Research Quarterly					Not related to leprosy
440	Abstracts of 52nd EASD Annual Meeting	Hilson Andrade	02/04/2025	unknown		2016	pringer Link					10.1007/s	http://link.springer.com/article/10.1007/s001				Diabetologia					Not related to leprosy
441	62ND ASMS Conference on Mass Spectrometry and Allied Topics	Hilson Andrade	02/04/2025	Judith Sjoberg		2014	pringer Link					10.1007/s	http://link.springer.com/article/10.1007/s133				Journal of The American Society for Mass Spectrometry					Not related to leprosy
442	Drug repurposing for cancer therapy	Hilson Andrade	02/04/2025	Ying XiaMing SunHai HuangWei-Lin Jin		2024	pringer Link					10.1038/s	http://link.springer.com/article/10.1038/s413				Signal Transduction and Targeted Therapy					Not related to leprosy
443	63RD ASMS Conference on Mass Spectrometry and Allied Topics	Hilson Andrade	02/04/2025	unknown		2015	pringer Link					10.1007/s	http://link.springer.com/article/10.1007/s133				Journal of The American Society for Mass Spectrometry					Not related to leprosy
444	Human and translational immunology in the third millennium: progress, challenges and opportunities	Hilson Andrade	02/04/2025	Ioanna E. GalaniEynav KlechevskyEvangelo		2019	pringer Link					10.1038/s	http://link.springer.com/article/10.1038/s415				Nature Immunology					Not related to AI
445	Abstracts 18th European Congress of Pathology Berlin, Germany, September 8–13, 2001	Hilson Andrade	02/04/2025	unknown		2001	pringer Link					10.1007/B	http://link.springer.com/article/10.1007/BF0				Virchows Archiv					Not related to AI
446	De novo genome assembly and annotation of the medicinal plant Tinospora cordifolia (Willd.) Miers ex Hook. f. & Thom's	Hilson Andrade	02/04/2025	Namitha RManasa KHSanthosh N HegdeNo		2023	pringer Link					10.1007/s	http://link.springer.com/article/10.1007/s101				Functional & Integrative Genomics					Not related to AI
447	Looking back and moving forward in medicinal chemistry	Hilson Andrade	02/04/2025	unknown		2023	pringer Link					10.1038/s	http://link.springer.com/article/10.1038/s414				Nature Communications					Not related to AI
448	Abstracts Scientific Papers Honorary Lectures Categorical Courses Workshops State-of-the-Art Symposia	Hilson Andrade	02/04/2025	unknown		1999	pringer Link					10.1007/B	http://link.springer.com/article/10.1007/BF0				European Radiology					Not related to leprosy
449	The interpretation of uncertainty in ecological rationality	Hilson Andrade	02/04/2025	Anastasia KozyrevaRalph Hertwig		2021	pringer Link					10.1007/s	http://link.springer.com/article/10.1007/s112				Synthese					Not related to leprosy
450	Editorial: The Real Technology Revolution: Technology Justice	Hilson Andrade	02/04/2025	Neth DañoStefano Prato		2019	pringer Link					10.1057/s	http://link.springer.com/article/10.1057/s413				Development					Not related to leprosy
451	Recycling side-effects into clinical markers for drug repositioning	Hilson Andrade	02/04/2025	Miquel Duran-FrigolaPatrick Aloy		2012	pringer Link					10.1186/g	http://link.springer.com/article/10.1186/gm3				Genome Medicine					Not related to leprosy
452	Abstracts of the papers presented in the international conference of Indian Virological Society, VIROCON 2022 on "Emerging and re-emerg	Hilson Andrade	02/04/2025	unknown		2023	pringer Link					10.1007/s	http://link.springer.com/article/10.1007/s133				VirusDisease					Not related to AI
453	Genetic pain loss disorders	Hilson Andrade	02/04/2025	Annette LischkaPetra LassuthovaArman Çak		2022	pringer Link					10.1038/s	http://link.springer.com/article/10.1038/s415				Nature Reviews Disease Primers					Not related to AI
454	Antimicrobial Resistance: A Cause for Global Concern	Hilson Andrade	02/04/2025	Rubina LawrenceEbenezer Jeyakumar		2013	pringer Link					10.1186/1	http://link.springer.com/article/10.1186/1753				BMC Proceedings					Not related to AI
455	SaVanT: a web-based tool for the sample-level visualization of molecular signatures in gene expression profiles	Hilson Andrade	02/04/2025	David LopezDennis MontoyaMichael Ambros		2017	pringer Link					10.1186/s	http://link.springer.com/article/10.1186/s128				BMC Genomics					Not related to AI
456	Meeting the challenges of NTM-PD from the perspective of the organism and the disease process: innovations in drug development and de	Hilson Andrade	02/04/2025	Roald van der LaanAndy SnabiliéMarko Obra		2022	pringer Link					10.1186/s	http://link.springer.com/article/10.1186/s129				Respiratory Research					Not related to leprosy
457	Inflammation and immune dysfunction in Parkinson disease	Hilson Andrade	02/04/2025	Malú Gámez TanseyRebecca L. WallingsMac		2022	pringer Link					10.1038/s	http://link.springer.com/article/10.1038/s415				Nature Reviews Immunology					Not related to leprosy
458	What is the relative impact of primary health care quality and conditional cash transfer program in child mortality?	Hilson Andrade	02/04/2025	Anya Pimentel Gomes Fernandes Vieira-Mey		2019	pringer Link					10.17269/	http://link.springer.com/article/10.17269/s41				Canadian Journal of Public Health					Not related to leprosy
459	Laboratory diagnostics for human Leishmania infections: a polymerase chain reaction-focussed review of detection and identification meth	Hilson Andrade	02/04/2025	Ineka GowNicholas C. SmithDamien StarkJo		2022	pringer Link					10.1186/s	http://link.springer.com/article/10.1186/s130				Parasites & Vectors					Surveys
460	Host–parasite co-evolution and its genomic signature	Hilson Andrade	02/04/2025	Dieter EbertPeter D. Fields		2020	pringer Link					10.1038/s	http://link.springer.com/article/10.1038/s415				Nature Reviews Genetics					Not related to leprosy
461	Drug Repurposing: An Effective Tool in Modern Drug Discovery	Hilson Andrade	02/04/2025	V. S. KulkarniV. AlagarsamyV. R. SolomonP.		2023	pringer Link					10.1134/S	http://link.springer.com/article/10.1134/S10				Russian Journal of Bioorganic Chemistry					Not related to leprosy
462	Saussurea costus (Falc.) Lipsch.: a comprehensive review of its pharmacology, phytochemicals, ethnobotanical uses, and therapeutic pote	Hilson Andrade	02/04/2025	Ruchika KumariMadhvi NegiPalak ThakurHir		2024	pringer Link					10.1007/s	http://link.springer.com/article/10.1007/s002				Naunyn-Schmiedeberg's Archives of Pharmacology					Surveys
463	Drug Repurposing of Generic Drugs: Challenges and the Potential Role for Government	Hilson Andrade	02/04/2025	Karel H. van der PolMohamad AljofanOlivier		2023	pringer Link					10.1007/s	http://link.springer.com/article/10.1007/s402				Applied Health Economics and Health Policy					Not related to leprosy
464	Exploiting large-scale drug-protein interaction information for computational drug repurposing	Hilson Andrade	02/04/2025	Ruifeng LiuNarender SinghGregory J TawaA		2014	pringer Link					10.1186/1	http://link.springer.com/article/10.1186/1471				BMC Bioinformatics					Not related to leprosy
465	Abstracts Scientific Exhibition CompuRAD Golden Mile Audiovisual	Hilson Andrade	02/04/2025	unknown		1999	pringer Link					10.1007/B	http://link.springer.com/article/10.1007/BF0				European Radiology					Not related to leprosy
466	Differential transcriptomic and metabolic profiles of M. africanum- and M. tuberculosis-infected patients after, but not before, drug treatment	Hilson Andrade	02/04/2025	L D TientcheuJ MaertzdorfJ WeinerI M Adetif		2015	pringer Link					10.1038/g	http://link.springer.com/article/10.1038/gene				Genes & Immunity					Not related to leprosy
467	Tuberculosis: current scenario, drug targets, and future prospects	Hilson Andrade	02/04/2025	Priyanka BoseAmit K. HaritRatnesh DasSam		2021	pringer Link					10.1007/s	http://link.springer.com/article/10.1007/s000				Medicinal Chemistry Research					Not related to leprosy
468	Reviews and bibliographical notices	Hilson Andrade	02/04/2025	unknown		1847	pringer Link					10.1007/B	http://link.springer.com/article/10.1007/BF0				Dublin Quarterly Journal of Medical Science					Surveys
469	Abstracts from the 4th Asian Conference in Pharmaceutical Sciences (Asia Pharm IV)	Hilson Andrade	02/04/2025	unknown		2019	pringer Link					10.1186/s	http://link.springer.com/article/10.1186/s129				BMC Proceedings					Not related to leprosy
470	Functional Diversity as a New Framework for Understanding the Ecology of an Emerging Generalist Pathogen	Hilson Andrade	02/04/2025	Aaron MorrisJean-François GuéganM. Eric B		2016	pringer Link					10.1007/s	http://link.springer.com/article/10.1007/s103				EcoHealth					Not related to leprosy
471	Drug repurposing: a systematic review on root causes, barriers and facilitators	Hilson Andrade	02/04/2025	Nithya KrishnamurthyAlyssa A. GrimshawSy		2022	pringer Link					10.1186/s	http://link.springer.com/article/10.1186/s129				BMC Health Services Research					Surveys
472	Multiscale modeling of mucosal immune responses	Hilson Andrade	02/04/2025	Yongguo MeiVida AbediAdria CarboXiaoying		2015	pringer Link					10.1186/1	http://link.springer.com/article/10.1186/1471				BMC Bioinformatics					Not related to leprosy
473	Infectious eye disease in the 21st century—an overview	Hilson Andrade	02/04/2025	Gerry ClareJohn H. KempenCarlos Pavésio		2024	pringer Link					10.1038/s	http://link.springer.com/article/10.1038/s414				Eye					Surveys
474	An esteemed maximum utility pattern mining: special children assessment analysis	Hilson Andrade	02/04/2025	R. DhanalakshmiB. MuthukumarR. Aroulcane		2023	pringer Link					10.1007/s	http://link.springer.com/article/10.1007/s137				Progress in Artificial Intelligence					Not related to leprosy
475	Deciphering drug discovery and microbial pathogenesis research in tuberculosis during the two decades of postgenomic era using entity m	Hilson Andrade	02/04/2025	Vinit KumarGauri ShankarYusuf Akhter		2023	pringer Link					10.1007/s	http://link.springer.com/article/10.1007/s002				Archives of Microbiology					Not related to leprosy
476	Title Conversational Learning in the Age of ChatGPT	Hilson Andrade	02/04/2025	Fiachra Long		2024	pringer Link					10.1007/s	http://link.springer.com/article/10.1007/s112				Studies in Philosophy and Education					Not related to leprosy
477	An aligned corpus of Spanish bibles	Hilson Andrade	02/04/2025	Gerardo SierraGemma Bel-EnguixAmeyali D		2024	pringer Link					10.1007/s	http://link.springer.com/article/10.1007/s105				Language Resources and Evaluation					Not related to leprosy
478	A systematic scoping review of the Social Vulnerability Index as applied to natural hazards	Hilson Andrade	02/04/2025	Mary Angelica PainterSameer H. ShahGwen		2024	pringer Link					10.1007/s	http://link.springer.com/article/10.1007/s110				Natural Hazards					Surveys
479	Abstracts of the XX World Allergy Congress™ 2007 December 2-6, 2007, Bangkok, Thailand	Hilson Andrade	02/04/2025	unknown		2007	pringer Link					10.1186/1	http://link.springer.com/article/10.1186/1939				World Allergy Organization Journal					Not related to leprosy
480	Drug Repurposing: a Shortcut to New Biological Entities	Hilson Andrade	02/04/2025	Nutan RaoTushar PoojariCharvi PoojaryRuks		2022	pringer Link					10.1007/s	http://link.springer.com/article/10.1007/s110				Pharmaceutical Chemistry Journal					Not related to leprosy
481	Interactions of urbanisation, climate variability, and infectious disease dynamics: insights from the Coimbatore district of Tamil Nadu	Hilson Andrade	02/04/2025	Sudha SureshGowhar MerajPankaj KumarDe		2023	pringer Link					10.1007/s	http://link.springer.com/article/10.1007/s106				Environmental Monitoring and Assessment					Not related to AI
482	Automatic classification of registered clinical trials towards the Global Burden of Diseases taxonomy of diseases and injuries	Hilson Andrade	02/04/2025	Ignacio AtalJean-David ZeitounAurélie Névé		2016	pringer Link					10.1186/s	http://link.springer.com/article/10.1186/s128				BMC Bioinformatics					Not related to AI
483	Respiratory tract infections: an update on the complexity of bacterial diversity, therapeutic interventions and breakthroughs	Hilson Andrade	02/04/2025	Avani PanickarAnand ManoharanAnand Anb		2024	pringer Link					10.1007/s	http://link.springer.com/article/10.1007/s002				Archives of Microbiology					Not related to leprosy
484	Asclepiadoideae subfamily (Apocynaceae): ethnopharmacology, biological activities and chemophenetics based on pregnane glycosides	Hilson Andrade	02/04/2025	Thalisson Amorim de SouzaFrancisca Sabrin		2024	pringer Link					10.1007/s	http://link.springer.com/article/10.1007/s111				Phytochemistry Reviews					Not related to leprosy
485	Botany, traditional usages, phytochemistry, pharmacology, and toxicology of Guilandina bonduc L.: a systematic review	Hilson Andrade	02/04/2025	Prabhu SrinivasanKalaimathi KarunanithiAyy		2024	pringer Link					10.1007/s	http://link.springer.com/article/10.1007/s002				Naunyn-Schmiedeberg's Archives of Pharmacology					Surveys
486	Development, Pharmacokinetics and Antimalarial Evaluation of Dose Flexible 3D Printlets of Dapsone for Pediatric Patients	Hilson Andrade	02/04/2025	Adnan A. KhanTahir KhurooEman M Moham		2024	pringer Link					10.1208/s	http://link.springer.com/article/10.1208/s122				AAPS PharmSciTech					Not related to leprosy
487	Measuring the complexity of the law: the United States Code	Hilson Andrade	02/04/2025	Daniel Martin KatzM. J. Bommarito II		2014	pringer Link					10.1007/s	http://link.springer.com/article/10.1007/s105				Artificial Intelligence and Law					Not related to leprosy
488	Abstracts from the 36th Annual Meeting of the Society of General Internal Medicine	Hilson Andrade	02/04/2025	unknown		2013	pringer Link					10.1007/s	http://link.springer.com/article/10.1007/s116				Journal of General Internal Medicine					Not related to AI
489	DrugRep-HeSiaGraph: when heterogenous siamese neural network meets knowledge graphs for drug repurposing	Hilson Andrade	02/04/2025	Zahra GhorbanaliFatemeh Zare-MirakabadN		2023	pringer Link					10.1186/s	http://link.springer.com/article/10.1186/s128				BMC Bioinformatics					Not related to leprosy
490	2020 CIS Annual Meeting: Immune Deficiency & Dysregulation North American Conference	Hilson Andrade	02/04/2025	unknown		2020	pringer Link					10.1007/s	http://link.springer.com/article/10.1007/s108				Journal of Clinical Immunology					Not related to AI
491	International EcoHealth One Health Congress 2016	Hilson Andrade	02/04/2025	unknown		2016	pringer Link					10.1007/s	http://link.springer.com/article/10.1007/s103				EcoHealth					Not related to AI
492	Report on "The Second United Nations World Geospatial Information Congress (UN-WGIC) Pre-Event"	Hilson Andrade	02/04/2025	Sneha KourArpan DawnSmariddha SahaBipl		2023	pringer Link					10.1007/s	http://link.springer.com/article/10.1007/s400				National Academy Science Letters					Not related to AI
493	Thiosemicarbazone Derivatives in Search of Potent Medicinal Agents: QSAR Approach (A Review)	Hilson Andrade	02/04/2025	M. I. AhmadE. VegS. JoshiA. R. KhanT. Khar		2024	pringer Link					10.1134/S	http://link.springer.com/article/10.1134/S107				Russian Journal of General Chemistry					Surveys
494	Nineteenth Meeting of the European Neurological Society 20–24 June 2009, Milan, Italy	Hilson Andrade	02/04/2025	unknown		2009	pringer Link					10.1007/s	http://link.springer.com/article/10.1007/s004				Journal of Neurology					Not related to AI
495	NERO: a biomedical named-entity (recognition) ontology with a large, annotated corpus reveals meaningful associations through text embe	Hilson Andrade	02/04/2025	Kanix WangRobert StevensHalima Alachram		2021	pringer Link					10.1038/s	http://link.springer.com/article/10.1038/s415				npj Systems Biology and Applications					Not related to AI
496	Oral Presentation Abstracts, 25th Annual Meeting of the International Association of Medical Science Educators, June 12–17, 2021	Hilson Andrade	02/04/2025	unknown		2021	pringer Link					10.1007/s	http://link.springer.com/article/10.1007/s406				Medical Science Educator					Not related to AI
497	Regulatory genome variants in human susceptibility to infection	Hilson Andrade	02/04/2025	Amalio TelentiJulia di Iulio		2020	pringer Link					10.1007/s	http://link.springer.com/article/10.1007/s004				Human Genetics					Not related to AI
498	Targeted protein degradation: advances in drug discovery and clinical practice	Hilson Andrade	02/04/2025	Guangcai ZhongXiaoyu ChangWeilin XieXiar		2024	pringer Link					10.1038/s	http://link.springer.com/article/10.1038/s413				Signal Transduction and Targeted Therapy					Not related to AI
499	2020 Joint conference of the Italian Association of Miology (AIM) and the Italian Association for the study of the Peripheral Nervous Systen	Hilson Andrade	02/04/2025	unknown		2022	pringer Link					10.1007/s	http://link.springer.com/article/10.1007/s100				Neurological Sciences					Not related to AI

Id	title	data extractor	date of data extraction	author	journal	year	source	pages	volume	abstract	document_type	doi	url	affiliation	ıor_keywo	keywords	publisher	issn	ınguag	note	lection_criteı	Selection
500	Developing a Nomogram for Risk Prediction of Severe Hand-Foot-and-Mouth Disease in Children	Hilson Andrade	02/04/2025	Bin WangHuifen FengPing HuangDejian Dan		2019	pringer Link					10.1007/s	http://link.springer.com/article/10.1007/s120				The Indian Journal of Pediatrics					Not related to leprosy
501	AI-based healthcare: a new dawn or apartheid revisited?	Hilson Andrade	02/04/2025	Alice ParfettStuart TownleyKristofer Allerfeldt		2021	pringer Link					10.1007/s	http://link.springer.com/article/10.1007/s001				AI & SOCIETY					Not related to leprosy
502	Drug combinations: a strategy to extend the life of antibiotics in the 21st century	Hilson Andrade	02/04/2025	Mike TyersGerard D. Wright		2019	pringer Link					10.1038/s	http://link.springer.com/article/10.1038/s415				Nature Reviews Microbiology					Not related to AI
503	Scientific Programme — Abstracts	Hilson Andrade	02/04/2025	unknown		2003	pringer Link					10.1007/B	http://link.springer.com/article/10.1007/BF0				European Radiology					Not related to AI
504	Host-microbe interactions in the pathogenesis and clinical course of sarcoidosis	Hilson Andrade	02/04/2025	Pleiades T. InaokaMasato ShonoMishio Kam		2019	pringer Link					10.1186/s	http://link.springer.com/article/10.1186/s129				Journal of Biomedical Science					Not related to AI
505	ECR 2005 – Scientific Programme – Abstracts	Hilson Andrade	02/04/2025	unknown		2005	pringer Link					10.1007/s	http://link.springer.com/article/10.1007/s104				European Radiology Supplements					Not related to AI
506	Spatial transcriptomics in human biomedical research and clinical application	Hilson Andrade	02/04/2025	Weining HuYin ZhangJunpu MeiXiaodong Fa		2023	pringer Link					10.1007/s	http://link.springer.com/article/10.1007/s441				Current Medicine					Not related to AI
507	Abstracts from the 2019 Annual Meeting of the Society of General Internal Medicine	Hilson Andrade	02/04/2025	unknown		2019	pringer Link					10.1007/s	http://link.springer.com/article/10.1007/s116				Journal of General Internal Medicine					Not related to AI
508	Artificial Intelligence Based Skin Classification Using GMM	Hilson Andrade	02/04/2025	M. MonishaA. SureshM. R. Rashmi		2018	pringer Link					10.1007/s	http://link.springer.com/article/10.1007/s109				Journal of Medical Systems					Included
509	Computational drug repositioning with attention walking	Hilson Andrade	02/04/2025	Jong-Hoon ParkYoung-Rae Cho		2024	pringer Link					10.1038/s	http://link.springer.com/article/10.1038/s415				Scientific Reports					Not related to leprosy
510	Integrating single-cell multi-omics and prior biological knowledge for a functional characterization of the immune system	Hilson Andrade	02/04/2025	Philipp Sven Lars SchäferDaniel DimitrovEdu		2024	pringer Link					10.1038/s	http://link.springer.com/article/10.1038/s415				Nature Immunology					Not related to AI
511	Weber local descriptor for image analysis and recognition: a survey	Hilson Andrade	02/04/2025	Arnab BanerjeeNibaran DasK. C. Santosh		2022	pringer Link					10.1007/s	http://link.springer.com/article/10.1007/s003				The Visual Computer					Surveys
512	Abstracts from the 2017 Society of General Internal Medicine Annual Meeting	Hilson Andrade	02/04/2025	unknown		2017	pringer Link					10.1007/s	http://link.springer.com/article/10.1007/s116				Journal of General Internal Medicine					Not related to AI
513	Tryp: a dataset of microscopy images of unstained thick blood smears for trypanosome detection	Hilson Andrade	02/04/2025	Esla Timothy AnzakuMohammed Aliy Moham		2023	pringer Link					10.1038/s	http://link.springer.com/article/10.1038/s415				Scientific Data					Duplicated
514	Abstracts of the 9th Tanzania Health Summit	Hilson Andrade	02/04/2025	unknown		2023	pringer Link					10.1186/s	http://link.springer.com/article/10.1186/s129				BMC Proceedings					Not related to AI
515	Prognostic factors in the prediction of chronic wound healing by electrical stimulation	Hilson Andrade	02/04/2025	Dr D. CukjatiM. Robnik-ŠikonjaS. ReberšekI.		2001	pringer Link					10.1007/B	http://link.springer.com/article/10.1007/BF0				Medical and Biological Engineering and Computing					Not related to AI
516	Macrophage polarization and future perspectives: a comprehensive review	Hilson Andrade	02/04/2025	Yashwanth C. YellankiMallanagouda M. Patil		2024	pringer Link					10.1007/s	http://link.springer.com/article/10.1007/s439				Discover Materials					Surveys
517	Advancing against drug-resistant tuberculosis: an extensive review, novel strategies and patent landscape	Hilson Andrade	02/04/2025	Meghana N. PatelArchita J. PatelManish N. N		2024	pringer Link					10.1007/s	http://link.springer.com/article/10.1007/s002				Naunyn-Schmiedeberg's Archives of Pharmacology					Surveys
518	Additional Neural Matrix Factorization model for computational drug repositioning	Hilson Andrade	02/04/2025	Xinxing YangIbrahim ZamitYu LiuJieyue He		2019	pringer Link					10.1186/s	http://link.springer.com/article/10.1186/s128				BMC Bioinformatics					Not related to leprosy
519	Abstracts of the 8th Tanzania Health Summit, October 2021	Hilson Andrade	02/04/2025	unknown		2022	pringer Link					10.1186/s	http://link.springer.com/article/10.1186/s129				BMC Proceedings					Not related to AI
520	Exploring tissue architecture using spatial transcriptomics	Hilson Andrade	02/04/2025	Anjali RaoDalia BarkleyGustavo S. FrançaIta		2021	pringer Link					10.1038/s	http://link.springer.com/article/10.1038/s415				Nature					Not related to leprosy
521	Metabolite changes in blood predict the onset of tuberculosis	Hilson Andrade	02/04/2025	January Weiner 3rdJeroen MaertzdorfJayne		2018	pringer Link					10.1038/s	http://link.springer.com/article/10.1038/s414				Nature Communications					Not related to leprosy
522	Explainable drug repurposing via path based knowledge graph completion	Hilson Andrade	02/04/2025	Ana JiménezMaría José MerinoJuan ParrasS		2024	pringer Link					10.1038/s	http://link.springer.com/article/10.1038/s415				Scientific Reports					Duplicated
523	A novel Markov Blanket-based repeated-fishing strategy for capturing phenotype-related biomarkers in big omics data	Hilson Andrade	02/04/2025	Hongkai LiZhongshang YuanJiadong JiJing X		2016	pringer Link					10.1186/s	http://link.springer.com/article/10.1186/s128				BMC Genetics					Not related to AI
524	Abstracts from the 35th Annual Meeting of the Society of General Internal Medicine	Hilson Andrade	02/04/2025	unknown		2012	pringer Link					10.1007/s	http://link.springer.com/article/10.1007/s116				Journal of General Internal Medicine					Not related to AI
525	Comparing Short-Term Univariate and Multivariate Time-Series Forecasting Models in Infectious Disease Outbreak	Hilson Andrade	02/04/2025	Daniel Bouzon Nagem AssadJavier CaraMig		2022	pringer Link					10.1007/s	http://link.springer.com/article/10.1007/s115				Bulletin of Mathematical Biology					Not related to AI
526	Network or regression-based methods for disease discrimination: a comparison study	Hilson Andrade	02/04/2025	Xiaoshuai ZhangZhongshang YuanJiadong J		2016	pringer Link					10.1186/s	http://link.springer.com/article/10.1186/s128				BMC Medical Research Methodology					Duplicated
527	ECR 2016 Book of Abstracts - B - Scientific Sessions and Clinical Trials in Radiology	Hilson Andrade	02/04/2025	unknown		2016	pringer Link					10.1007/s	http://link.springer.com/article/10.1007/s132				Insights into Imaging					Not related to AI
528	Skin melanoma classification using ROI and data augmentation with deep convolutional neural networks	Hilson Andrade	02/04/2025	Khalid M. HosnyMohamed A. KassemMoham		2020	pringer Link					10.1007/s	http://link.springer.com/article/10.1007/s110				Multimedia Tools and Applications					Not related to leprosy
529	A fuzzy expert system approach using multiple experts for dynamic follow-up of endemic diseases	Hilson Andrade	02/04/2025	Apurba BanerjeeArun Kumar MajumderAnup		1994	pringer Link					10.1007/B	http://link.springer.com/article/10.1007/BF0				Sadhana					Excluded by QA1
530	Identification of Early Warning Signals of Infectious Diseases in Hospitals by Integrating Clinical Treatment and Disease Prevention	Hilson Andrade	02/04/2025	Lei ZhangMin-ye LiChen ZhiMin ZhuHui Ma		2024	pringer Link					10.1007/s	http://link.springer.com/article/10.1007/s115				Current Medical Science					Not related to AI
531	Healthy Living: The European Congress of Epidemiology, 2015	Hilson Andrade	02/04/2025	Esther BolsLuc SmitsMatty Weijenberg		2015	pringer Link					10.1007/s	http://link.springer.com/article/10.1007/s106				European Journal of Epidemiology					Not related to AI
532	Skin image analysis for detection and quantitative assessment of dermatitis, vitiligo and alopecia areata lesions: a systematic literature revi	Hilson Andrade	02/04/2025	Athanasios KallipolitisKonstantinos Moutselo		2025	pringer Link					10.1186/s	http://link.springer.com/article/10.1186/s129				BMC Medical Informatics and Decision Making					Surveys
533	Structural Implications of Mutations Conferring Rifampin Resistance in Mycobacterium leprae	Hilson Andrade	02/04/2025	Sundeep Chaitanya VedithiSony MalhotraMa		2018	pringer Link					10.1038/s	http://link.springer.com/article/10.1038/s415				Scientific Reports					Not related to AI
534	A review of the current trends in computational approaches in drug design and metabolism	Hilson Andrade	02/04/2025	Russell B. O. OumaSilas M. NgariJoshua K.		2024	pringer Link					10.1186/s	http://link.springer.com/article/10.1186/s129				Discover Public Health					Surveys
535	20th IUPAB International Congress, 45th SBBf Congress and 50th Annual Meeting of SBBq	Hilson Andrade	02/04/2025	unknown		2021	pringer Link					10.1007/s	http://link.springer.com/article/10.1007/s125				Biophysical Reviews					Not related to AI
536	20th ISoP Annual Meeting "Integrated pharmacovigilance for safer patients" 8–10 November 2021 Muscat, Oman (Hybrid meeting)	Hilson Andrade	02/04/2025	unknown		2021	pringer Link					10.1007/s	http://link.springer.com/article/10.1007/s402				Drug Safety					Not related to AI
537	Prediction of comorbid diseases using weighted geometric embedding of human interactome	Hilson Andrade	02/04/2025	Pakeeza AkramLi Liao		2019	pringer Link					10.1186/s	http://link.springer.com/article/10.1186/s129				BMC Medical Genomics					Not related to AI
538	Emerging infectious disease surveillance using a hierarchical diagnosis model and the Knox algorithm	Hilson Andrade	02/04/2025	Mengying WangBingqing YangYunpeng LiuY		2023	pringer Link					10.1038/s	http://link.springer.com/article/10.1038/s415				Scientific Reports					Not related to leprosy
539	Data Mining and in Silico Analysis of Ethiopian Traditional Medicine: Unveiling the Therapeutic Potential of Rumex abyssinicus Jacq.	Hilson Andrade	02/04/2025	Lemessa Etana BultumGwangmin KimSeon-		2024	pringer Link					10.1007/s	http://link.springer.com/article/10.1007/s120				Cell Biochemistry and Biophysics					Not related to AI
540	High Throughput and Computational Repurposing for Neglected Diseases	Hilson Andrade	02/04/2025	Helen W. HernandezMelinda SoeungKimberl		2018	pringer Link					10.1007/s	http://link.springer.com/article/10.1007/s110				Pharmaceutical Research					Not related to AI
541	Clinical and translational values of spatial transcriptomics	Hilson Andrade	02/04/2025	Linlin ZhangDongsheng ChenDongli SongXia		2022	pringer Link					10.1038/s	http://link.springer.com/article/10.1038/s413				Signal Transduction and Targeted Therapy					Not related to leprosy
542	Abstracts of 45th National Conference of Association of Clinical Biochemists of India (ACBICON 2018)	Hilson Andrade	02/04/2025	unknown		2018	pringer Link					10.1007/s	http://link.springer.com/article/10.1007/s122				Indian Journal of Clinical Biochemistry					Not related to AI
543	Skin cancer diagnosis based on deep transfer learning and sparrow search algorithm	Hilson Andrade	02/04/2025	Hossam Magdy BalahaAsmaa El-Sayed Has		2023	pringer Link					10.1007/s	http://link.springer.com/article/10.1007/s005				Neural Computing and Applications					Not related to leprosy
544	Abstracts of the 10th Tanzania Health Summit	Hilson Andrade	02/04/2025	unknown		2024	pringer Link					10.1186/s	http://link.springer.com/article/10.1186/s129				BMC Proceedings					Not related to AI
545	Performance evaluation of drug synergy datasets using computational intelligence approaches	Hilson Andrade	02/04/2025	Pooja RaniKamlesh DuttaVijay Kumar		2024	pringer Link					10.1007/s	http://link.springer.com/article/10.1007/s110				Multimedia Tools and Applications					Not related to leprosy
546	Predicting the effect of climate change on the spatiotemporal distribution of two endangered plant species, Silene leucophylla Boiss. and S	Hilson Andrade	02/04/2025	Aliaa Muhammad RefaatAshraf Mohamed Yo		2024	pringer Link					10.1186/s	http://link.springer.com/article/10.1186/s430				Beni-Suef University Journal of Basic and Applied Scien					Not related to leprosy
547	Impact of industry 4.0 on healthcare systems of low- and middle- income countries: a systematic review	Hilson Andrade	02/04/2025	Joseph MwanzaArnesh TelukdarieTak Igusa		2023	pringer Link					10.1007/s	http://link.springer.com/article/10.1007/s125				Health and Technology					Surveys
548	A foundation model for clinician-centered drug repurposing	Hilson Andrade	02/04/2025	Kexin HuangPayal ChandakQianwen WangS		2024	pringer Link					10.1038/s	http://link.springer.com/article/10.1038/s415				Nature Medicine					Not related to leprosy
549	Deep migration learning-based recognition of diseases and insect pests in Yunnan tea under complex environments	Hilson Andrade	02/04/2025	Zhaowen LiJihong SunYingming ShenYing Ya		2024	pringer Link					10.1186/s	http://link.springer.com/article/10.1186/s130				Plant Methods					Not related to leprosy
550	Innovative technologies to address neglected tropical diseases in African settings with persistent sociopolitical instability	Hilson Andrade	02/04/2025	Tsegahun ManyazewalGail DaveyCharlotte H		2024	pringer Link					10.1038/s	http://link.springer.com/article/10.1038/s414				Nature Communications					Surveys
551	The transcriptional legacy of developmental stochasticity	Hilson Andrade	02/04/2025	Sara BallouzRisa Karakida KawaguchiMaria		2023	pringer Link					10.1038/s	http://link.springer.com/article/10.1038/s414				Nature Communications					Not related to AI
552	A review of computational drug repositioning: strategies, approaches, opportunities, challenges, and directions	Hilson Andrade	02/04/2025	Tamer N. JaradaJon G. RokneReda Alhajj		2020	pringer Link					10.1186/s	http://link.springer.com/article/10.1186/s133				Journal of Cheminformatics					Surveys
553	Abstracts from the 57th European Society of Human Genetics (ESHG) Conference: Oral Presentations	Hilson Andrade	02/04/2025	unknown		2024	pringer Link					10.1038/s	http://link.springer.com/article/10.1038/s414				European Journal of Human Genetics					Not related to AI
554	In search of better science: on the epistemic costs of systematic reviews and the need for a pluralistic stance to literature search	Hilson Andrade	02/04/2025	Andrea Polonioli		2020	pringer Link					10.1007/s	http://link.springer.com/article/10.1007/s111				Scientometrics					Surveys
555	Machine learning arbitrated prediction of disease prevalence due to air pollution over United Arab Emirates	Hilson Andrade	02/04/2025	Jagadish Kumar Mogaraju		2023	pringer Link					10.1007/s	http://link.springer.com/article/10.1007/s118				Air Quality, Atmosphere & Health					Not related to leprosy
556	Machine learning approaches to identify systemic lupus erythematosus in anti-nuclear antibody-positive patients using genomic data and e	Hilson Andrade	02/04/2025	Chih-Wei ChungSeng-Cho ChouTzu-Hung H		2024	pringer Link					10.1186/s	http://link.springer.com/article/10.1186/s130				BioData Mining					Not related to leprosy
557	Phenotypic drug discovery: recent successes, lessons learned and new directions	Hilson Andrade	02/04/2025	Fabien VincentArsenio NuedaJonathan LeeM		2022	pringer Link					10.1038/s	http://link.springer.com/article/10.1038/s415				Nature Reviews Drug Discovery					Not related to leprosy
558	Introducing a novel deep convolutional neural network to detect skin cancer in thermographic images	Hilson Andrade	02/04/2025	Junfeng Ma		2024	pringer Link					10.1007/s	http://link.springer.com/article/10.1007/s125				Journal of Optics					Not related to leprosy
559	Proceedings of the 3rd IPLeiria's International Health Congress	Hilson Andrade	02/04/2025	Catarina Cardoso TomásEmanuel OliveiraD.		2016	pringer Link					10.1186/s	http://link.springer.com/article/10.1186/s129				BMC Health Services Research					Not related to AI
560	World Congress on Osteoporosis, Osteoarthritis and Musculoskeletal Diseases (WCO-IOF-ESCEO 2020): Poster Abstracts	Hilson Andrade	02/04/2025	unknown		2020	pringer Link					10.1007/s	http://link.springer.com/article/10.1007/s001				Osteoporosis International					Not related to leprosy
561	ESHNR 2023 Book of Abstracts	Hilson Andrade	02/04/2025	unknown		2023	pringer Link					10.1186/s	http://link.springer.com/article/10.1186/s132				Insights into Imaging					Not related to AI
562	Proceedings: 15th Asia-Pacific Federation for Clinical Biochemistry and Laboratory Medicine (APFCB) Congress 2019 from 17-20 Novemb	Hilson Andrade	02/04/2025	unknown		2019	pringer Link					10.1007/s	http://link.springer.com/article/10.1007/s122				Indian Journal of Clinical Biochemistry					Not related to AI
563	Abstracts from the International Science Symposium on HIV and Infectious Diseases (ISSHID 2019): Infectious diseases	Hilson Andrade	02/04/2025	unknown		2020	pringer Link					10.1186/s	http://link.springer.com/article/10.1186/s128				BMC Infectious Diseases					Not related to AI
564	Precision information extraction for rare disease epidemiology at scale	Hilson Andrade	02/04/2025	William Z. KariampuzhaGioconda AlyeaSue		2023	pringer Link					10.1186/s	http://link.springer.com/article/10.1186/s129				Journal of Translational Medicine					Not related to AI
565	Towards next-generation diagnostic pathology: AI-empowered label-free multiphoton microscopy	Hilson Andrade	02/04/2025	Shu WangJunlin PanXiao ZhangYueying LiW		2024	pringer Link					10.1038/s	http://link.springer.com/article/10.1038/s413				Light: Science & Applications					Not related to leprosy
566	Leprosy surveillance study in a highly endemic Brazilian area using leprosy specific serologic tests and IFNγ whole blood assay	Hilson Andrade	02/04/2025	Aline do Carmo GonçalvesEmerith Mayra Hu		2020	pringer Link					10.1007/s	http://link.springer.com/article/10.1007/s100				European Journal of Clinical Microbiology & Infectious D					Not related to AI
567	33	Hilson Andrade	02/04/2025	unknown		2021	pringer Link					10.1007/s	http://link.springer.com/article/10.1007/s004				Virchows Archiv					Not related to AI
568	Abstracts	Hilson Andrade	02/04/2025	unknown		2018	pringer Link					10.1007/s	http://link.springer.com/article/10.1007/s004				Virchows Archiv					Duplicated
569	Decoding Drug Discovery: Exploring A-to-Z In Silico Methods for Beginners	Hilson Andrade	02/04/2025	Hezha O. RasulDlzar D. GhafourBakhtyar K.		2024	pringer Link					10.1007/s	http://link.springer.com/article/10.1007/s120				Applied Biochemistry and Biotechnology					Not related to AI
570	Deep ensemble learning for automatic medicinal leaf identification	Hilson Andrade	02/04/2025	Silky SacharAnuj Kumar		2022	pringer Link					10.1007/s	http://link.springer.com/article/10.1007/s418				International Journal of Information Technology					Not related to leprosy
571	Abstracts of the 19th Congress of the European Geriatric Medicine Society	Hilson Andrade	02/04/2025	unknown		2023	pringer Link					10.1007/s	http://link.springer.com/article/10.1007/s419				European Geriatric Medicine					Not available
572	Abstracts from the 2020 Annual Meeting of the Society of General Internal Medicine	Hilson Andrade	02/04/2025	unknown		2020	pringer Link					10.1007/s	http://link.springer.com/article/10.1007/s116				Journal of General Internal Medicine					Not related to AI
573	Abstracts of the 82nd Annual Meeting of the German Society for Experimental and Clinical Pharmacology and Toxicology (DGPT) and the	Hilson Andrade	02/04/2025	unknown		2016	pringer Link					10.1007/s	http://link.springer.com/article/10.1007/s002				Naunyn-Schmiedeberg's Archives of Pharmacology					Not related to AI
574	Abstracts of the 51st Annual Conference of the Italian Society of Neurology	Hilson Andrade	02/04/2025	unknown		2020	pringer Link					10.1007/s	http://link.springer.com/article/10.1007/s100				Neurological Sciences					Not related to AI
575	Towards Diagnosis of Autoimmune Blistering Skin Diseases Using Deep Neural Network	Hilson Andrade	02/04/2025	Manbir SinghManinder SinghDipankar DeSa		2023	pringer Link					10.1007/s	http://link.springer.com/article/10.1007/s118				Archives of Computational Methods in Engineering					Surveys
576	Deep learning-based sign language recognition system for static signs	Hilson Andrade	02/04/2025	Ankita WadhawanParteek Kumar		2020	pringer Link					10.1007/s	http://link.springer.com/article/10.1007/s005				Neural Computing and Applications					Not related to leprosy
577	Abstracts from the 37th Annual Meeting of the Society of General Internal Medicine	Hilson Andrade	02/04/2025	unknown		2014	pringer Link					10.1007/s	http://link.springer.com/article/10.1007/s116				Journal of General Internal Medicine					Not related to AI
578	Twenty-second Meeting of the European Neurological Society 9–12 June, 2012 Prague, Czech Republic	Hilson Andrade	02/04/2025	unknown		2012	pringer Link					10.1007/s	http://link.springer.com/article/10.1007/s004				Journal of Neurology					Not related to AI
579	Abstracts from the 2016 Society of General Internal Medicine Annual Meeting	Hilson Andrade	02/04/2025	unknown		2016	pringer Link					10.1007/s	http://link.springer.com/article/10.1007/s116				Journal of General Internal Medicine					Not related to AI
580	A lightweight attention-based deep learning facial recognition system for multiple genetic syndromes	Hilson Andrade	02/04/2025	Tawqeer Ul IslamTawseef Ayoub Shaikh		2024	pringer Link					10.1007/s	http://link.springer.com/article/10.1007/s410				International Journal of Data Science and Analytics					Not related to leprosy
581	Digital Transformation in the Control of Neglected Tropical Diseases: A Scoping Review	Hilson Andrade	02/04/2025	Douglas de Souza RodriguesBruna de Paula		2024	pringer Link					10.1007/s	http://link.springer.com/article/10.1007/s404				Current Tropical Medicine Reports					Surveys
582	Abstracts	Hilson Andrade	02/04/2025	unknown		2019	pringer Link					10.1007/s	http://link.springer.com/article/10.1007/s004				Virchows Archiv					Duplicated
583	Joint Congress of European Neurology 31 May–3 June 2014 Istanbul, Turkey	Hilson Andrade	02/04/2025	unknown		2014	pringer Link					10.1007/s	http://link.springer.com/article/10.1007/s004				Journal of Neurology					Not related to AI
584	Abstracts from the 57th European Society of Human Genetics (ESHG) Conference: Hybrid Posters	Hilson Andrade	02/04/2025	unknown		2024	pringer Link					10.1038/s	http://link.springer.com/article/10.1038/s414				European Journal of Human Genetics					Not related to AI
585	World Congress on Osteoporosis, Osteoarthritis and Musculoskeletal Diseases (WCO-IOF-ESCEO 2023)	Hilson Andrade	02/04/2025	unknown		2023	pringer Link					10.1007/s	http://link.springer.com/article/10.1007/s405				Aging Clinical and Experimental Research					Not related to AI
586	World Congress on Osteoporosis, Osteoarthritis and Musculoskeletal Diseases (WCO-IOF-ESCEO 2024)	Hilson Andrade	02/04/2025	unknown		2024	pringer Link					10.1007/s	http://link.springer.com/article/10.1007/s405				Aging Clinical and Experimental Research					Not related to AI
587	Abstracts of HAEMATOCON 2024	Hilson Andrade	02/04/2025	unknown		2024	pringer Link					10.1007/s	http://link.springer.com/article/10.1007/s122				Indian Journal of Hematology and Blood Transfusion					Not related to leprosy
588	Abstracts from the 2024 Annual Meeting of the Society of General Internal Medicine	Hilson Andrade	02/04/2025	unknown		2024	pringer Link					10.1007/s	http://link.springer.com/article/10.1007/s116				Journal of General Internal Medicine					Not related to AI
589	Machine Learning and Deep Learning Based Time Series Prediction and Forecasting of Ten Nations' COVID-19 Pandemic	Hilson Andrade	02/04/2025	Yogesh KumarApeksha KoulSukhpreet Kaur		2022	pringer Link					10.1007/s	http://link.springer.com/article/10.1007/s429				SN Computer Science					Not related to leprosy
590	Artificial Intelligence in Drug Discovery: A Comprehensive Review of Data-driven and Machine Learning Approaches	Hilson Andrade	02/04/2025	Hyunho KimEunyoung KimIngoo LeeBongsu		2020	pringer Link					10.1007/s	http://link.springer.com/article/10.1007/s122				Biotechnology and Bioprocess Engineering					Surveys
591	Deep Semantic Segmentation Assisted Region-of-Interest Sensitive Deep Spatio-Textural Feature Learning Framework for Leprosy Detect	Hilson Andrade	02/04/2025	R. JitendraJay B. SimhaShinu AbhiVineet K.		2024	pringer Link					10.1007/s	http://link.springer.com/article/10.1007/s429				SN Computer Science					Excluded by QA3
592	Heuristic Segmentation Assisted Deep-Spatial Feature Learning Model for Leprosy Detection	Hilson Andrade	02/04/2025	R JitendraJay B SimhaShinu AbhiVineet K Cl		2024	pringer Link					10.1007/s	http://link.springer.com/article/10.1007/s429				SN Computer Science					Excluded by QA3
593	ECR 2019: Book of Abstracts	Hilson Andrade	02/04/2025	unknown		2019	pringer Link					10.1186/s	http://link.springer.com/article/10.1186/s132				Insights into Imaging					Not related to AI
594	35th European Congress of Pathology – Abstracts	Hilson Andrade	02/04/2025	unknown		2023	pringer Link					10.1007/s	http://link.springer.com/article/10.1007/s004				Virchows Archiv					Not related to AI
595	Identification of psychosocial problems in patients with Hansen's disease by analysis of computerized resources	Hilson Andrade	02/04/2025	Helene, LM and Rocha, M	Revista da Escola	1998	PubMed	l99--207	32													Not related to AI
596	Pathogen-specific epitopes as epidemiological tools for defining the magnitude of Mycobacterium leprae transmission in areas endemic for	Hilson Andrade	02/04/2025	Martins, Marcia VSB and	PLoS neglected tr	2012	PubMed	e1616	6								Public Library of Science San Francisco, USA					Included
597	Bi-PSSM: Position specific scoring matrix based intelligent computational model for identification of mycobacterial membrane proteins	Hilson Andrade	02/04/2025	Khan, Muslim and Hayat,	Journal of Theoret	2017	PubMed	l16--124	435								Elsevier					Duplicated
598	Clinical performance of AI-integrated risk assessment pooling reveals cost savings even at high prevalence of COVID-19	Hilson Andrade	02/04/2025	Kamari, Farzin and Eller, I	Scientific Reports	2024	PubMed	8853	14								Nature Publishing Group UK London					Not related to leprosy
599	The application of digital health as a nursing solution for leprosy patients during the COVID-19 pandemic: A systematic review.	Hilson Andrade	02/04/2025	Dahoklory, Dorsina Fransi	JPMA. The Journa	2023	PubMed	l70--S17	73													Surveys

Id	title	data extractor	date of data extraction	author	journal	year	source	pages	volume	abstract	document_type	doi	url	affiliation	or_keywo	keywords	publisher	issn	languag	note	lection_criter	Selection
600	A novel integrated molecular and serological analysis method to predict new cases of leprosy amongst household contacts	Hilson Andrade	02/04/2025	Gama, Rafael Silva and S	PLoS neglected tr	2019	PubMed	000740	13								Public Library of Science San Francisco, CA USA					Included
601	Proceedings from the CIHLMU occupational safety and health symposium 2019 “Protecting workers’ health: global challenges and opportu	Hilson Andrade	02/04/2025	Gidi, Netsanet Workneh and Suraya, Anna ar		2020	PubMed	14	14													Not related to leprosy
602	Network or regression-based methods for disease discrimination: a comparison study	Hilson Andrade	02/04/2025	Zhang, Xiaoshuai and Yua	BMC medical rese	2016	PubMed	1--9	16								Springer					Included
603	Performance Evaluation of No-Code Artificial Intelligence Models for the Detection of Acid-Fast Bacilli: A Comparative Analysis of Three Mc	Hilson Andrade	02/04/2025	Arya, Yash and Konduru,	Cureus	2024	PubMed		16								Cureus Inc.					Excluded by QA3
604	Fuzzy spectral clustering for automated delineation of chronic wound region using digital images	Hilson Andrade	02/04/2025	Dhane, Dhiraj Manohar ar	Computers in biolc	2017	PubMed	51--560	89								Elsevier					Not related to AI
605	Algorithm design for a cytokine release assay of antigen-specific in vitro stimuli of circulating leukocytes to classify leprosy patients and hou	Hilson Andrade	02/04/2025	Mar{\c{c}}al, Pedro Henrique	Ferreira and de	2022	PubMed	ofac036	9													Included
606	In vivo partial reprogramming by bacteria promotes adult liver organ growth without fibrosis and tumorigenesis	Hilson Andrade	02/04/2025	Hess, Samuel and Kendal	Cell Reports Medi	2022	PubMed		3								Elsevier					Not related to AI
607	Comparison of the Diagnostic Accuracy of Teledermoscopy, Face-to-Face Examinations and Artificial Intelligence in the Diagnosis of Melan	Hilson Andrade	02/04/2025	Yazdanparast, Taraneh ar	Indian Journal of D	2024	PubMed	296--300	69								Medknow					Not related to leprosy
608	Mapping suitability for Buruli ulcer at fine spatial scales across Africa: a modelling study	Hilson Andrade	02/04/2025	Simpson, Hope and Tabal	PLoS neglected tr	2021	PubMed	000915	15								Public Library of Science San Francisco, CA USA					Not related to leprosy
609	Risk predictions of hospital-acquired pressure injury in the intensive care unit based on a machine learning algorithm	Hilson Andrade	02/04/2025	Tehrany, Pooya M and Za	International Wour	2023	PubMed	768--377	20								Wiley Online Library					Not related to leprosy
610	MycoBASE: expanding the functional annotation coverage of mycobacterial genomes	Hilson Andrade	02/04/2025	Garcia, Benjamin J and D	BMC genomics	2015	PubMed	1--9	16								Springer					Not related to AI
611	The burden of subclinical TB in Nigeria	Hilson Andrade	02/04/2025	Odume, B and Ogbudebe	Public Health Actic	2024	PubMed	181--185	14								International Union Against Tuberculosis and Lung Disea					Not related to leprosy
612	The application of biomedical engineering techniques to the diagnosis and management of tropical diseases: a review	Hilson Andrade	02/04/2025	Ibrahim, Fatimah and Thic	Sensors	2015	PubMed	947--699	15								MDPI					Surveys
613	Optimization of rifamycin B fermentation in shake flasks via a machine-learning-based approach	Hilson Andrade	02/04/2025	Bapat, Prashant M and W	Biotechnology and	2004	PubMed	201--208	86								Wiley Online Library					Excluded by QA1
614	Investigating bacterial volatilome for the classification and identification of mycobacterial species by HS-SPME-GC-MS and machine learni	Hilson Andrade	02/04/2025	Beccaria, Marco and Fran	Molecules	2021	PubMed	4600	26								MDPI					Included
615	Prediction of the occurrence of leprosy reactions based on Bayesian networks	Hilson Andrade	02/04/2025	de Andrade Rodrigues, Ra	Frontiers in Medici	2023	PubMed	1233220	10								Frontiers Media SA					Not related to AI
616	Deep learning for AI-based diagnosis of skin-related neglected tropical diseases: A pilot study	Hilson Andrade	02/04/2025	Yotsu, Rie R and Ding, Zh	PLOS Neglected T	2023	PubMed	001123	17								Public Library of Science San Francisco, CA USA					Included
617	Therapeutic application of machine learning in psoriasis: A Prisma systematic review	Hilson Andrade	02/04/2025	Lunge, Snehal Balvant an	Journal of cosmeti	2023	PubMed	378--382	22								Wiley Online Library					Surveys
618	Strategies for drug target identification in Mycobacterium leprae	Hilson Andrade	02/04/2025	Acebr{\'o}n-Garc{\'\i}a-de-l	Drug Discovery To	2021	PubMed	569--157	26								Elsevier					Not related to AI
619	Artificial intelligence and digital health in improving primary health care service delivery in LMICs: A systematic review	Hilson Andrade	02/04/2025	Saif-Ur-Rahman, KM and	Journal of Evidenc	2023	PubMed	303--320	16								Wiley Online Library					Surveys
620	Guidelines for the use and interpretation of assays for monitoring autophagy	Hilson Andrade	02/04/2025	Klionsky, Daniel J and Abc	autophagy	2021	PubMed	1--382	17								Taylor \& Francis					Not related to leprosy
621	Laboratory perspectives for Leprosy: Diagnostic, prognostic and predictive tools	Hilson Andrade	02/04/2025	Malhotra, Kiran Preet and	Indian Journal of F	2022	PubMed	300--S30	65								Medknow					Surveys
622	Evaluating Advanced Machine Learning Models for Histopathological Diagnosis of Hansen Disease	Hilson Andrade	02/04/2025	Vargas-Clavijo, Mariana a	The American Jou	2022	PubMed	10--1097									LWW					Excluded by QA3
623	Prediction of rifampicin resistance beyond the RRDR using structure-based machine learning approaches	Hilson Andrade	02/04/2025	Portelli, Stephanie and My	Scientific Reports	2020	PubMed	18120	10								Nature Publishing Group UK London					Included
624	Effectiveness of using AI-driven hotspot mapping for active case finding of tuberculosis in Southwestern Nigeria	Hilson Andrade	02/04/2025	Alege, Abiola and Hashmi	Tropical Medicine	2024	PubMed	99	9								MDPI					Not related to leprosy
625	The potential role of artificial intelligence in the clinical management of Hansen’s disease (leprosy)	Hilson Andrade	02/04/2025	Deps, Patr{\'\i}cia D and Y	Frontiers in Medici	2024	PubMed	1338598	11								Frontiers Media SA					Surveys
626	Machine learning and its application in skin cancer	Hilson Andrade	02/04/2025	Das, Kinnor and Cockerel	International Journ	2021	PubMed	13409	18								MDPI					Not related to leprosy
627	Artificial intelligence in dermatology and healthcare: An overview	Hilson Andrade	02/04/2025	Pai, Varadraj Vasant and I	Indian Journal of D	2021	PubMed	457--467	87								Scientific Scholar					Surveys
628	Artificial intelligence: Its role in dermatopathology	Hilson Andrade	02/04/2025	Jartarkar, Shishira R	Indian Journal of D	2023	PubMed	549--552	89								Scientific Scholar					Surveys
629	Artificial intelligence on diagnostic aid of leprosy: a systematic literature review	Hilson Andrade	02/04/2025	Fernandes, Jacks Renan	Journal of Clinical	2023	PubMed	180	13								MDPI					Surveys
630	Harnessing the Power of Artificial Intelligence in Dermatology: A Comprehensive Commentary	Hilson Andrade	02/04/2025	Kololgi, Shreyas P and La	Indian Journal of D	2023	PubMed	678--681	68								Medknow					Surveys
631	Adapting motorbikes for independent use by people with disability	Hilson Andrade	02/04/2025	Owens, Jesse		2009	l Digital Library			In much of the world motorbikes		10.1145/1	https://doi.org/10.1145/1592700.1592704				Association for Computing Machinery					Not related to leprosy
632	The U in Crypto Stands for Usable: An Empirical Study of User Experience with Mobile Cryptocurrency Wallets	Hilson Andrade	02/04/2025	Voskobojnikov, Artemij and Wiese, Oliver and		2021	l Digital Library			In a corpus of 45,821 app reviev		10.1145/3	https://doi.org/10.1145/3411764.			thematic a	Association for Computing Machinery					Not related to leprosy
633	Combining lexical and syntactic features for detecting content-dense texts in news	Hilson Andrade	02/04/2025	Yang, Yinfei and Nenkova	J. Artif. Int. Res.	2017	l Digital Lit	179–219	60	Content-dense news report important factual information about an event in direct, succi							AI Access	1076-9757				Not related to leprosy
634	ICBET '23: Proceedings of the 2023 13th International Conference on Biomedical Engineering and Technology	Hilson Andrade	02/04/2025			2023	l Digital Library										Association for Computing Machinery					Duplicated
635	PCI '23: Proceedings of the 27th Pan-Hellenic Conference on Progress in Computing and Informatics	Hilson Andrade	02/04/2025			2023	l Digital Library										Association for Computing Machinery					Not related to leprosy
636	Restless Bandits visiting Villages: A Preliminary Study on distributing Public Health Services	Hilson Andrade	02/04/2025	Bhattacharya, Biswarup		2018	l Digital Library			Distributing public health service		10.1145/3	https://doi.org/10.1145/3209811.			p-functiona	Association for Computing Machinery					Not related to leprosy
637	A passive controlled hand rehabilitation instrument	Hilson Andrade	02/04/2025	Xu, Hongzhen and Shen, Manlin and Duan,		2021	l Digital Lit	433–436		The number of people with uppe		10.1145/3	https://doi.org/10.1145/3436286.			speech co	Association for Computing Machinery					Not related to AI
638	Crowdsourcing Ground Truth for Medical Relation Extraction	Hilson Andrade	02/04/2025	Dumitrache, Anca and Arc	ACM Trans. Intera	2018	l Digital Library		8	Cognitive computing systems re		10.1145/3	https://doi.org/10.1145/3152889			relation ex	Associatio	2160-6455				Not related to leprosy
639	A hand biomechanics workstation	Hilson Andrade	02/04/2025	Thompson, David E. and I	SIGGRAPH Comp	1988	l Digital Lit	335–343	22	Interactive graphics for hand sui		10.1145/3	https://doi.org/10.1145/378456.3			orthopedic	Associatio	0097-8930				Not related to leprosy
640	The principles of traditional Chinese medicine in the therapy of Vitiligo, an investigation based on data mining and network pharmacology	Hilson Andrade	02/04/2025	Chen, Qian and Jia, Yuxi and Zhang, Xueying		2025	l Digital Lit	776–781		Objective: To explore the combi		10.1145/3	https://doi.org/10.1145/3706890.			Data minir	Association for Computing Machinery					Not related to leprosy
641	Recognizing Common Skin Diseases in the Philippines Using Image Processing and Machine Learning Classification	Hilson Andrade	02/04/2025	CASUAYAN DE GOMA, JOEL and DEVARA		2020	l Digital Lit	68–72		Skin disease is prevalent in trop		10.1145/3	https://doi.org/10.1145/3418688.			Skin disea	Association for Computing Machinery					Included
642	Transforming Dermatological Diagnosis: Deep Learning Approaches for Skin Disease Detection in the Digital Era	Hilson Andrade	02/04/2025	Yadav, Ankit and Sharma, Vinay and Raj, Ga		2024	l Digital Lit	254–262		Recent years have seen a signi		10.1145/3	https://doi.org/10.1145/3660853.3660924				Association for Computing Machinery					Not related to leprosy
643	ISAIMS '24: Proceedings of the 2024 5th International Symposium on Artificial Intelligence for Medicine Science	Hilson Andrade	02/04/2025			2024	l Digital Library										Association for Computing Machinery					Not related to leprosy
644	AICCONF '24: Proceedings of the Cognitive Models and Artificial Intelligence Conference	Hilson Andrade	02/04/2025			2024	l Digital Library										Association for Computing Machinery					Not related to leprosy
645	Measurement of retinal vessel widths from fundus images based on 2-D modeling	Hilson Andrade	02/04/2025	Lowell, J. and Hunter, A.	IEEE Transactions	2004	: Digital Lit	196-120	23	Changes in retinal vessel diame		10.1109/TMI.2004.830524				Retinal vessels;Diabe		1558-254X				Not related to leprosy
646	A knowledge based system using multiple expert modules for monitoring leprosy/spl minus/an endemic disease	Hilson Andrade	02/04/2025	Banerjee, A. and Majumd	IEEE Transactions	1994	: Digital Lit	173-186	24	An environment with multiple ex		10.1109/21.281418				Knowledge based sys		2168-2909				Not related to AI
647	AI Driven Edge Device for Screening Skin Lesion and Its Severity in Peripheral Communities	Hilson Andrade	02/04/2025	Jaikishore, Chathura.N. and Udutalapally, Ve		2021	: Digital Lit	1-6		It is vital to treat any skin disord		10.1109/INDICON52576.2021.9691666				Performance evaluati		2325-9418				Included
648	Enhancing Diagnosis of Infectious Skin Disease: A Professional Approach using Deep Learning for Leprosy Detection	Hilson Andrade	02/04/2025	Gawali, Chetan Jitendra and Subbulakshmi,		2024	: Digital Lit	1-7		Leprosy remains a significant pu		10.1109/ICCCNT61001.2024.10725616				Deep learning;Suppo		2473-7674				Excluded by QA3
649	Leprosy Diagnosis using Explainable Artificial Intelligence Techniques	Hilson Andrade	02/04/2025	Baweja, Asis Kaur and Aditya, S and Kancha		2023	: Digital Lit	551-556		LRprosy, also known as Hanser		10.1109/ICSCDS56580.2023.10104958				Sensitivity;Computational modeling;Flight recording;Computer arch						Included
650	Disease Data Modeling by Using Machine Learning Approach	Hilson Andrade	02/04/2025	Wang, Gunawan and Wardhana, Rakha Gus		2024	: Digital Lit	285-289		Since Covid outbreak in 2020, Ir		10.1109/ICITACEE62763.2024.10762785				Support vector machi		2996-1300				Excluded by QA3
651	Canine Skin-Disease Detection Using CNN	Hilson Andrade	02/04/2025	Harshitha, B. Sai and Pujitha, M. Vani and Cl		2024	: Digital Lit	1-6	1	Skin diseases in dogs often leac		10.1109/ICKECS61492.2024.10616528				Training;Microorganisms;Accuracy;Dogs;Predictive models;Propag						Not related to leprosy
652	Improved Skin Disease Classification Using Generative Adversarial Network	Hilson Andrade	02/04/2025	Mondal, Bisakh and Das, Nibaran and Santo		2020	: Digital Lit	520-525		Identifying skin diseases, such a		10.1109/CBMS49503.2020.00104				Skin;Diseases;Trainir		2372-9198				Included
653	Diagnosis of Leprosy through AI-based Mobile Application	Hilson Andrade	02/04/2025	Nikam, Atharva and Ranade, Pranita and Go		2023	: Digital Lit	1-6		No digital service is available to		10.1109/OTCON56053.2023.10114031				Skin;Mobile applications;Lesions;Sustainable development;Disease						Excluded by QA1
654	Applying Artificial Intelligence and Deep Learning to Identify Neglected Tropical Skin Disorders	Hilson Andrade	02/04/2025	Pattnayak, Parthasarathi and Mohanty, Arpee		2024	: Digital Lit	1-6		Visual inspection plays a signific		10.1109/INOCON60754.2024.10511323				Deep learning;Training;Visualization;Technological innovation;Neur						Included
655	Leprosy lesion recognition using convolutional neural networks	Hilson Andrade	02/04/2025	Baweja, Harjatin Singh and Parhar, Tanvir		2016	: Digital Lit	141-145	1	Leprosy, also known as Hansen		10.1109/ICMLC.2016.7860891				Lesions;Training;Con		2160-1348				Included
656	Predicting Severity from Electronic Health Records of Leprosy Patients using Ensemble Learning	Hilson Andrade	02/04/2025	Mehta, Jalpa and Kalla, Mukesh		2023	: Digital Lit	1-6		Electronic Health Records (EHR		10.1109/WCONF58270.2023.10235056				Data preprocessing;Medical services;Forestry;Delays;Ensemble lea						Excluded by QA3
657	Deep Facial Diagnosis: Deep Transfer Learning From Face Recognition to Facial Diagnosis	Hilson Andrade	02/04/2025	Jin, Bo and Cruz, Leandro	IEEE Access	2020	: Digital Lit	649-123	8	The relationship between face a		10.1109/ACCESS.2020.3005687				Face;Diseases;Face		2169-3536				Included
658	Skin Disease Detection And Classifcation	Hilson Andrade	02/04/2025	Pugazhenthi, V and Naik,	International Journ	2019	Snowballing	396-400	6			10.22161/i	https://ijaers.com/uploads/issue_files/53-IJAERS-MAY-2019-35-SkinDisease.pdf									Excluded by QA1
659	Enhancing the Diagnosis of Skin Neglected Tropical Diseases by Artificial Neural Networks Using Evolutionary Algorithms: Implementation	Hilson Andrade	02/04/2025	Nyatte, Steyve and Perabi, Steve and Abess		2023	Snowballing	475--496					https://link.springer.com/chapter/10.1007/978-981-99-0248-4_32									Included
660	Application of Clustering Technique with Kohonen Self-organizing Maps for the Epidemiological Analysis of Leprosy	Hilson Andrade	02/04/2025	Da Silva, Ygor Eugênio Dutra and Salgado, (		2019	Snowballing	295--309					https://link.springer.com/chapter/10.1007/978-3-030-01057-7_24									Included
661	Data Mining Using Clustering Techniques as Leprosy Epidemiology Analyzing Model	Hilson Andrade	02/04/2025	Dutra Da Silva, Ygor Eugenio and Salgado, (		2018	Snowballing	284--293					https://link.springer.com/chapter/10.1007/978-3-642-45062-4_48									Included
662	A New Rotation Invariant Weber Local Descriptor for Recognition of Skin Diseases	Hilson Andrade	02/04/2025	Pal, A. and Das, N. and Sarkar, S. and Gang		2013	Snowballing	355-360					https://link.springer.com/chapter/10.1007/978-3-642-45062-4_48									Included
663	An SVM based skin disease identification using Local Binary Patterns	Hilson Andrade	02/04/2025	Das, N. and Pal, A. and Mazumder, S. and S		2013	Snowballing	208-211					https://ieeexplore.ieee.org/document/6686372									Included
664	Leprosy Skin Lesion Detection: An AI Approach Using Few Shot Learning in a Small Clinical Dataset	Hilson Andrade	02/04/2025	R Beesetty, SA Reddy, S Modali, G Sunkara,		2023	Snowballing	89-102					https://www.ijl.org.in/published-articles/29062023223603/1-R-Beesetty-et-al-Final.pdf									Included
665	Dermatological disease detection using image processing and artificial neural network	Hilson Andrade	02/04/2025	Yasir, Rahat and Rahman, Md. Ashiqur and A		2014	Snowballing	687-690					https://ieeexplore.ieee.org/document/7026918									Included

Id	title	data extractor	date of data extraction	author	journal	year	source	pages	volume	abstract	document_type	doi	url	affiliation	ıor_keywo	keywords	publisher	issn	ınguag	note	lection_criter	Selection

Id	title	data extractor	date of data extraction	author	journal	year	source	pages	volume	abstract	document_type	doi	url	affiliation	nor_keywo	keywords	publisher	issn	nguag	note	lection_criter	Selection

Id	title	data extractor	date of data extraction	author	journal	year	source	pages	volume	abstract	document_type	doi	url	affiliation	or_keywo	keywords	publisher	issn	nguag	note	lection_criter	Selection
