# Supplementary material for: On the usage of artificial intelligence in leprosy care: A systematic literature review
Source: PLoS Comput Biol. 2025 Jun 26;21(6):e1012550. doi: 10.1371/journal.pcbi.1012550 (PMC12225980; doi:10.1371/journal.pcbi.1012550)
Supplement: Attachment — Submitted filename: PLOS Computational Biology - review.pdf [file pcbi.1012550.s004.pdf]

PLOS Computational Biology
PCOMPBIOL-D-24-01734

Reviewer #1:
Hilson Gomes Vilar de Andrade et al collected and studied the publications using AI to study leprosy. The authors categorize all studies into 6 themes regarding different aspects of leprosy research. The authors followed PRISMA and screened for 395 publications and finally got 25 papers. The authors extracted information from the 25 papers and showed their thematic area, source of data, ML model types, problem types and metrics used to evaluate models. The manuscript could be better if the authors could address the comments below.
Thank you for your comments and suggestions to improve the quality of our work. We have addressed all of them, as described below.

Major comments:
1. (line 123) The search string is "((leprosy OR "hansen's disease") AND ("artificial intelligence" OR "deep learning" OR "machine learning") AND (prediction OR classification))." The results need to contain either "prediction" or "classification", but it looks like these two words could not cover all aspects of ML studies on leprosy and will have bias on more diagnostic studies. Other keyword like "regression", "treatment", "prognosis" or "monitor" are also supposed to be included.
Thank you for bringing this to our attention. To make our review more comprehensive, we decided to revise and redefine the search string. The updated version was defined as follows: ((leprosy OR "hansen's disease") AND ("artificial intelligence" OR "deep learning" OR "machine learning"), in order to cover all other aspects of ML, and minimize possible biases. With this new string, new studies were added to our systematic review and a whole new analysis was made.

2. (Line 139) When the two independent authors screened 376 papers, only 35 remains. What are the numbers of papers excluded by each of the 5 criteria (E1 to E5)? In E2, it excludes papers "less than six pages", does this "six pages" include supplementary information or references or additional information or it only refers to main text? Why is it "six" pages?
We have updated all this information, as we have changed the search string.

3. (Figure 6) Supervised learning models are usually used to solve two major types of problems, classification and regression. It looks like the studies collected in this manuscript are only about "classification" but none of them are about "regression", is it because the search string did not include "regression", or is it because the authors view regression problems also as classification problems, or is it due to there is no research using any regression models? It is expected that regression models could play essential roles in leprosy studies in like disease severity evaluation.
We do agree with your comment. As said previously, we have decided to change the search string, and now it is more comprehensive and it was able to include other types of problems.

Minor comments:

1. It is recommended to explain the meaning of “SLR” (although the reviewer could guess it’s “systematic literature review”) when it appears in the text for the first time. This is important since this abbreviation appears 42 times in the manuscript.

Thank you for pointing it out. We have revised the entire manuscript in order to fix all these minor issues.

Reviewer #2:
In this work, authors presented a systematic literature review about artificial intelligence in leprosy care. This review is well organized. The authors showed different types of data and the leprosy areas that AI researches mainly focused on. Authors also introduced fundamental concepts about individual AI techniques and metrics used in current AI models in leprosy. Authors claimed that this review can help researchers to explore the use of AI models in a more systematic way for leprosy care. However, there are some issues needed to be addressed to improve the quality of this review and make it distinguished from existing study.
Thank you for your comments and suggestions to improve our work. We have carefully read and addressed all these issues, as commented next.

Major:
1, In line 39, authors mentioned the limitation of another review of AI models in leprosy (Fernandes et al. PMID: 38202187), which is limited to the use of AI to diagnose leprosy. However, in this review, at least 18 out 25 AI models had been discussed in another review. Fernandes et al. also briefly mentioned the datasets and metrics used for these AI models. Therefore, this manuscript needs to be improved to make it distinguished from existing study.
Thank you for pointing it out. We have clarified our contribution by including this content in Introduction: "Differently from Fernandes et al. and Zinsou et al., which predominantly discuss the application of AI in diagnosing leprosy, our study advances the discourse by focusing on underexplored dimensions. Specifically, we expand the analysis to include the integration of AI technologies within clinical workflows for leprosy care, emphasizing real-world applications and the operational challenges involved."

Although authors introduce the fundamental concepts of AI techniques and metrics more comprehensively, authors should focus more on individual AI models. For instance, in line 520 "Seven articles in the SLR sample considered the use of CNN - [24,29,31,34,36,43,46,49].", authors simply summarized them into one sentence. But in fact, these methods use different CNN architecture which are worthy to mention and discuss. (descriptions of other models also have this issue such as SVM, NN and etc.)
Thank you for pointing this out. The section addressing the analysis of models based on Convolutional Neural Networks (CNN) has been expanded (lines 475 to 493, highlighted in blue), now providing detailed information on the architectures used by each author, the context in which they were applied, and the results obtained. The same adjustments were made in the sections addressing studies based on NN (lines 517 to 529), SVM (lines 540 to 556), and RF (lines 563 to 570). Additionally, a new section titled "Other Models and Approaches" was included, providing further details on studies that employed techniques such as Elastic-net Regression, XGBoost, ensemble and linear classifiers, as well as models for unsupervised clustering.

2, This review can be improved to better present to the audience a detailed picture of AI models in leprosy care in terms of their capability, pros and cons. Beyond simply listing the individual methods and techniques, authors should also mention the underlying rationale of individual methods to resolve the challenges in leprosy care.

Authors should also report the achievement or results (like accuracy, F1-score, recall, precision, …) of individual methods.
We have added a table at the end of RQ4, in order to report and summarize the models performance.

3, There are some missing genetic population groups discussion and accessibility to public data in RQ2. According to PMID: 34136455, Leprosy is directly related to the social factors in the population groups such as race, ethnicity, or skin color. In line 273, authors mentioned some datasets are public. The public data is always valuable for the entire community. To improve the accessibility and impacts of work, authors should provide a link to these public source data.
We have added a new table to complement information of Fig. 4, containing details of public datasets and their respective sources. Please, see Table 2.

4, In line 162, authors described the cut-off used to separate literatures into different quality levels (QA1). Why these cut-offs (>3, 1.2~3, <1.2) are reasonable?
The criteria employed were based on the Guidelines for Performing Systematic Literature Reviews in Software Engineering, proposed by Kitchenham and Charters (2007) – cited as reference number 18. This approach was also followed by Duarte et al. (2021) and Souza et al. (2019), who likewise adopted the exclusion criterion E6 to ensure the quality of the studies selected for the systematic literature review.

5, The number of studies in Fig 3 is 25. But the total number in Fig 5 and Fig 6 is different. Authors should clarify this point.
We have fixed it. Thank you.

Minor:
1, The authors should spell out the SLR when it firstly mentioned.
Thank you for pointing it out. We have revised the entire manuscript in order to fix all these issues.

2, Authors should also mention the AI in drug discovery for leprosy as a future direction.
Thank you for suggesting this. Our SLR suggests the use of AI for drug discovery in the context of leprosy is limited but offers substantial potential with respect to future research. We have added an additional paragraph and text in the manuscript to address this comment and link it to other potential areas for future research.

3, Authors mentioned the imbalanced dataset is an issue for accuracy. However, this issue also exists in other metrics (PMID: 25574450), author should better describe it to guide the community to use proper metrics.
We have added a new subsection to discuss data balancing.

4, All figures should have figure captions.
We have revised the entire manuscript in order to fix all these issues.

Reviewer #4:
This manuscript presents a systematic literature review (SLR) analyzing artificial intelligence (AI) applications in leprosy care, covering diagnosis, surveillance, treatment, and epidemiology. The authors identify that most AI applications in leprosy focus on image-based diagnosis, revealing significant gaps in other care phases.

Strengths:
The manuscript follows PRISMA guidelines. The manuscript's research questions (RQ1–RQ4) align well with clinical and technological needs. The paper presents detailed comparative tables and figures across studies.
Thank you for your comments and suggestions to improve our work. We have carefully read and addressed all these issues, as commented next.

Major concerns:
- The manuscript follows an empirical research format rather than a computational review format. For example, as a review paper, there are typically no methodological details about the review process itself. The evaluation metrics section should be trimmed. Sections like "Data extraction and coding" detract from the core computational analysis, while the algorithmic analysis should be expanded as the main focus.
Thank you for pointing it out. We have rewritten the metrics section, including, in lines 696 to 720 (highlighted in blue), a detailed analysis of the results presented by the proposed models, rather than merely describing the metrics used. This analysis includes Table 4, which provides an overview of the results of the models proposed by the selected studies, as well as the limitations of each.

- The authors provide only basic descriptions of ML techniques without analyzing their computational characteristics (in contrast, there are detailed formulas for evaluation metrics, which are less important). There is no analysis of algorithm details or future applications of specific methods.
Thank you for pointing it out. Regarding the algorithmic analysis, we agree with your observations and have rewritten the entire RQ3 section ("Which AI models are being used in leprosy research?") - lines 391 to 580. In this revised section, we categorize all AI-based models utilized in leprosy-related research (see Figure 5), their application across various thematic areas related to the disease (see Figure 6), and provide a comprehensive description of the techniques employed, including their advantages and disadvantages.

- The scope of the manuscript is unclear. While the authors attempt a systematic review of leprosy care, they fail to establish the necessity and uniqueness of the problem. There is no analysis of how this problem differs from other tasks and why solving and understanding it is important (for example, why leprosy image analysis is computationally unique). Although the authors make general points, these aren't specific to the problem setting. For example, from lines 97 to 101, the authors mention that AI could improve leprosy management but don't provide specifics.
We have clarified our contribution by including this content in Introduction: "Differently from Fernandes et al. and Zinsou et al., which predominantly discuss the application of AI in diagnosing leprosy, our study advances the discourse by focusing on underexplored dimensions. Specifically, we expand the analysis to include the

integration of AI technologies within clinical workflows for leprosy care, emphasizing real-world applications and the operational challenges involved."

When analysing works from the literature, we discuss how AI is being used for leprosy management (please see line 822, where we have included a new paragraph highlighting the lack of AI-based work for drug discovery for the treatment of leprosy, for example).

- The authors simply describe previous research without critical analysis and miss discussing why certain methods succeed or fail. Additionally, there is no synthesis of emerging methodological trends. These factors make it more like a catalog of papers than a critical review. For example, at line 201, the authors describe a gene signature identified by a paper but don't explain what it is, its implications, or its relevance to the topic.

Thank you for pointing it out. Based on your observation, we expanded the Conclusions section (lines 811 to 849, highlighted in blue), emphasizing the potential and implications of using AI for leprosy eradication, such as the discovery of new drugs (as highlighted by the work of Portelli et al.) and the subclinical diagnosis of leprosy (see the study proposed by Khan et al.), among other examples. Additionally, we provided a more detailed explanation of certain aspects of the study mentioned in this section, where the Random Forest (RF) technique is utilized for the subclinical diagnosis of household contacts (HHC). To this end, we rewrote the section addressing the RF technique (see lines 563 to 570) and included Table 4, which provides an overview of the reported results and observed limitations of all thirty selected studies.

Minor issues:

- Line 160. Is there a typo? It says "where S weight three times more than S"

Has been fixed, line 214.

- Line 246, what's the difference between the three data types, specifically in this setting?

The section RQ2: "What datasets are being used in AI-based leprosy research?" has been rewritten to clearly distinguish the three subsections it comprises: a) Type of Data (image, tabular, or hybrid - see Figure 4); b) Source of Public Datasets(see Table 2); and c) Data Records and Balancing (see Table 3).

- Line 407, most ML methods model the probability of an event, then what is the conceptual difference?

To highlight the conceptual differences between the models, we included Figure 5, which presents a categorization of AI models used in the literature, organized at different levels. Vertical categories represent additional subdivisions, while horizontal categories indicate the final categorization.

- Line 391, 340, for such interpretability, how to read the results?

To enhance the interpretability of the results presented in the RQ3 section ("Which AI models are being used in leprosy research?"), we revised the charts "Number of AI Models per Leprosy Thematic Area" (Figure 6) and "Number of AI Models per Data Type" (Figure 7).

Recommendations:

Due to these concerns, I cannot recommend this manuscript for publication in PCB. I suggest the authors: 1. Review other PCB publications to correct the format. 2. Discuss the problem setting more thoroughly. 3. Extend the scope, reduce descriptive content, and expand high-level discussion.

Considering the adjustments made based on your comments, which range from expanding the scope of the RSL through the redefinition of the search string, to refining the Research Questions (RQ) and culminating in a more detailed presentation of the findings, leading to a high-level discussion, we kindly request your reevaluation.
