# Supplementary material for: On the usage of artificial intelligence in leprosy care: A systematic literature review
Source: PLoS Comput Biol. 2025 Jun 26;21(6):e1012550. doi: 10.1371/journal.pcbi.1012550 (PMC12225980; doi:10.1371/journal.pcbi.1012550)
Supplement: Attachment — Submitted filename: RSL_minor review.pdf [file pcbi.1012550.s005.pdf]

PLOS Computational Biology
PCOMPBIOL-D-24-01734

Reviewers' comments:
Reviewer #1: The authors have addressed all my concerns in details. There are no more questions before I recommend to accept this manuscript.
Thank you for your comments to improve our work.

Reviewer #2: Thank authors for addressing my concerns. This version is more comprehensive and would serve as a good start point for researchers interested in studying leprosy care + AI.
Thank you for your comments to improve our work.

Reviewer #3: Authors have addressed the previous reviewers questions and made adjustments, recommending for acception.
Thank you for your comments to improve our work.

Reviewer #4:
Thank you for your comments to improve our work. We have addressed all your additional suggestions, and commented below:

1. I would like to first thank the author for providing extra introductions about how their work different from others. However, for my 3rd major concern, I would suggest the author add some context to discuss the specificity of leprosy care, possibly about why AI is important in leprosy care (compared to other disease) and what characteristics of leprosy made it suitable to be studied with AI.
Between lines 120 and 129, we have included a paragraph highlighting the importance of using AI to assist in leprosy treatment, taking into account certain specific aspects of the disease's clinical management, such as the occurrence of inflammatory reactions during treatment and antimicrobial resistance. We appreciate your contribution.

2. The author has mentioned specific preprocessing challenges for leprosy image data or tabular data. If possible, the author could also discuss how inconsistent preprocessing affects comparison across studies.
At the end of the section *AI Models Used in Leprosy Research*, a paragraph was added to highlight the impact of preprocessing techniques on the models' performance and generalization capacity. We sincerely appreciate your contribution.

3. The “RQ1: …”, “RQ2: …” headings still follow an empirical-study format. PCB reviews typically use descriptive subheadings (e.g. “Leprosy thematic areas addressed”), without labeling them “RQ#.” Please adjust to match the journal’s review-article style.
The section titles related to the RQs have been adjusted as follows: see lines 249 (previously: RQ1: Which leprosy thematic areas are being the AI research focus?; now: Leprosy thematic areas addressed), 306 (previously: RQ2: What datasets are being used in AI-based leprosy research?; now: Leprosy thematic areas addressed), 404 (previously: RQ3: Which AI models are being used in leprosy research?; now: AI models used in leprosy research), and 603 (previously: RQ4: How is the performance of AI models used in leprosy

research?; now: Performance evaluation of AI models used in leprosy research). We appreciate your valuable contribution.

4. A few citations in the text (e.g. [37], [40], [41]) don’t appear in the reference list; please ensure every in-text reference is included in the bibliography and vice versa.
The cited references were used in Table 1 (and not in the text) and have been included in the bibliography section on page 30. We appreciate your observation and confirm that a review has been conducted to ensure that all references in the text (and tables) are included in the bibliography and vice versa.

5. Other tips that may help increase the overall quality of the manuscipt:
a)“MDT” appears in the Abstract without definition. Please spell out “multidrug therapy (MDT)” at first use.
Thank you for your contribution. We have adjusted the Abstract section.

b)Several captions (e.g. for Figures 3–7) are too concise. They should be fully self-contained, describing what each axis represents, how categories are defined, sample sizes, and any color/shading conventions.
We also appreciate your contribution on this point. We have corrected all captions for Figures 3 to 8, adding descriptions of what each axis represents as well as definitions of the categories represented by the colors in the charts.
